# National trends in nine key minerals intake (quantity and source) among U.S. adults, 1999 to march 2020

**DOI:** 10.1186/s12937-024-00950-4

**Published:** 2024-05-17

**Authors:** Xuemin Yan, Xuanyang Wang, Jia Zhang, Zhu Ming, Can Zhang, Pingnan Ma, Qianmin Liu, Yuanyuan Xu, Licheng Cheng, Xibo Pang, Ying Li

**Affiliations:** Department of Nutrition and Food Hygiene, School of Public Health, the National Key Discipline, Harbin Medical University, 157 Baojian Road Harbin, P. R. 150081, Harbin, China

**Keywords:** Minerals, Sources, Trends, NHANES

## Abstract

**Background:**

Changes in economy and dietary guidelines brought a great shock to diet quality and meal behaviors, but if these transformations have extended to minerals intake and their sources was still poorly understood. It is essential to evaluate time trends in minerals intake and their sources to inform policy makers.

**Objective:**

To investigate trends in minerals intake and their sources among U.S. adults.

**Methods:**

This analysis used dietary data collected by 24-h recalls from U.S. adults (≥ 20 years) in NHANES (1999-March 2020). Minerals intake, age-adjusted percentage of participants meeting recommendations, and minerals sources were calculated among all participants and by population subgroups in each NHANES survey cycle. Weighted linear or logistic regression models were used to examine the statistical significance of time trends.

**Results:**

A total of 48223 U.S. adults were included in this analysis. From 1999 to March 2020, intake of calcium (from 0.94 to 1.02 g/day), magnesium (from 308.07 to 321.85 mg/day), phosphorus (from 1.24 to 1.30 g/day), and sodium (from 3.24 to 3.26 mg/day) from food and beverages (FB) and dietary supplements (DSs) significantly increased, and intake of iron (from 19.17 to 16.38 mg/day), zinc (from 16.45 to 14.19 mg/day), copper (from 1.79 to 1.38 mg/day), and potassium (from 2.65 to 2.50 g/day) from FB + DSs decreased (all *FDR* < 0.05). Additionally, age-adjusted percentage of participants meeting recommendations for calcium, phosphorus, sodium, and selenium significantly increased, that for iron, potassium, zinc, and copper decreased (all *FDR* < 0.05). Minerals intake and time trends in minerals intake were highly variable depending on age, gender, race/ethnicity, education, and income. For example, white, higher socioeconomic status participants had a higher minerals intake (e.g. iron, zinc, and copper), but had a greater decrease in minerals intake. Furthermore, the percentage of minerals from milks and DSs decreased, and that from beverages increased.

**Conclusion:**

From 1999 to March 2020, both minerals intake and their sources experienced a significant alteration among U.S. adults. Many differences in minerals intake and their food sources across sociodemographic characteristics appeared to narrow over time. Although some improvements were observed, important challenges, such as overconsumption of sodium and underconsumption of potassium, calcium, and magnesium, still remained among U.S. adults.

**Supplementary Information:**

The online version contains supplementary material available at 10.1186/s12937-024-00950-4.

## Background

Over the past two decades, suboptimal diet was still considered to be a key risk factor for chronic diseases [1, 2], the leading cause of death, and the third leading cause of disability-adjusted life-year loss [3] in the United States (U.S.). Paralleling with health impact, poor diet also possessed a substantial economic burden [4]. Improvement in diet quality could potentially result in broad and far-reaching health and economic benefits globally. Recent data indicated that the chronic diseases morbidity and mortality rate have stalled or deteriorated among U.S. adults [5–7], although macronutrient composition and overall dietary quality improved in the past decade [8–10]. Some other dietary factors linking with flattened or declined progress in chronic diseases prevention and control need to be taken into account.

Minerals, constituting human tissue [11] and biologically active substances [12], play a critical role in multiple functions (e.g., cognition, development, immune response, and thyroid function) [12–15]. Given their essential role in maintaining health, suboptimal minerals intake may increase the risk of chronic diseases [16–18]. Minerals were presented in a great variety of plant and animal foods, as well as in beverages and dietary supplements (DSs) [19–21]. Due to complexity of food components, the bioavailability of minerals varies greatly depending on their food sources [22]. Current evidences indicated that in addition to the quantity, food sources and forms of minerals were also of great importance in clarifying their associations with the risks of chronic diseases [23, 24].

Recently, U.S. adults experienced a significant alteration in dietary composition, diet quality, meal behaviors, and DSs use [25–28], and these alterations may have caused alterations in minerals intake and sources. To timely reflect the current level of minerals intake and sources among U.S. adults is an important basis to identify challenges and opportunities to improve Americans’ diets and reduce diet-related diseases costs. In the past decade, researchers have begun to pay attention to trends in energy, macronutrients, micronutrients, or a few selected items (e.g., sugar-sweetened beverages, ultra-processed food) of U.S. adults [8, 29, 30]. However, previous studies about trends in minerals intake focused on only a few minerals [31–33], or used older data [26, 32], or neglected sources [26]. Furthermore, potential differences among population subgroups have not been evaluated. Thus, it is essential to evaluate time trends in minerals intake and their sources in overall population and population subgroups among U.S. adults to discover prevalent, worsening, or potentially improving dietary problems.

In this context, we used data from 10 continuous cycles of the National Health and Nutrition Examination Survey (NHANES) from 1999–2000 to 2017-March 2020 to examine temporal trends in minerals intake and sources among U.S. adults. We also described these trends by sociodemographic characteristics.

## Methods

### Study population

NHANES is a cross-sectional, nationally representative study, providing data on demographic information, dietary intakes, and multiple health indicators of noninstitutionalized U.S. civilian population. The study design and methods about NHANES were described elsewhere. This study used data from 10 continuous cycles of NHANES (1999–2000 to 2017-March 2020). Data from 10 cycles of the NHANES included 58,744 participants aged 20 years or older. NHANES protocols were approved by the National Center for Health Statistics research ethics review board, and all participants provided informed consent [34]. A total of 48,223 participants were eligible for this study, after excluding participants who had extreme energy intake (< 500 or > 3,500 kcal/day for women, and < 800 or > 4,200 kcal/day for men), and who were pregnant at the time of examination (Supplementary Fig. 1).

### Minerals intake from foods and beverages and from DSs

In NHANES, dietary information was gathered by 24-hour recalls conducted by trained interviewers for two nonconsecutive days. During the interview, the participants were asked to report all foods and beverages (FB) consumed during the past 24-hour (midnight to midnight). Nutrients were estimated based on cycle-specific versions of the United States Department of Agriculture (USDA) Food and Nutrition Database for Dietary Studies. The estimated minerals intake from FB were the mean of minerals intake obtained through two 24-hour recalls.

30-Day Dietary Supplements Data was used to estimated minerals from DSs. In NHANES, participants were asked whether they used any prescription or non-prescription supplements in the past 30 days. Those reporting use were asked to provide the bottles of each supplement product or name, and the frequency, duration, as well as the serving form. The total daily dose of each supplemental minerals was estimated based on cycle-specific versions of dietary supplement database. The details of calculation method were described elsewhere [35].

The most recent dietary reference intakes (DRIs) issued by the National Academy of Sciences [36, 37] was used to estimate the percentage of the participants meeting Recommended Dietary Allowances (RDAs)/ Adequate Intakes (AIs) in each NHANES cycle.

### Classification of food groups

The major food sources included nine food groups defined by the USDA: Milk and milk products (Milks); Meat, poultry, fish and mixtures (Meats); Eggs (Eggs); Legumes, nuts and seeds (Nuts); Grain products (Grains); Fruits (Fruits); Vegetables (Vegetables); Fats, oils, and salad dressings (Oils); and Sugar, sweeteners, and beverages (Beverages). The food groups remained the same across NHANES survey cycles, allowing for an analysis of trends over time.

### Chronic diseases definitions

Cardiovascular disease (CVD) was defined by self-reported physician diagnoses, including coronary heart disease, angina/angina pectoris, heart attack, heart failure, and stroke. Diabetes was defined by self-reported doctor diagnosis of diabetes, taking diabetic medication, fasting blood glucose ≥ 126 mg/dL (7.0 mmol/L), or hemoglobin A1 ≥ 6.5% (48 mmol/mol). Chronic kidney disease (CKD) was defined by estimated glomerular filtration rate (eGFR) ≥ 60 ml/min/1.73m^2^ or a one-time urine albumin-to-creatinine ratio ≥ 30 mg/g. eGFR was calculated by the Chronic Kidney Disease-Epidemiology Collaboration equation [38]. We did not attempt to define persistent kidney dysfunction (at least 3 months), given that is impossible using a cross-sectional data set.

### Outcomes

The primary outcome of interest was trends in minerals intake from FB + DSs among all participants and by population subgroups (age, gender, race/ethnicity, education, and income). The second outcome was trends in minerals intake from FB + DSs by major sources among all participants and by population subgroups.

### Statistical analysis

Sampling weights were incorporated in all analyses to ensure nationally representative estimates. To minimize measurement error in dietary estimates, the absolute intake of minerals per day were adjusted for total energy intake to 2000 kcal/d using the residual method. The mean intake of minerals and age-adjusted percentages of the participants meeting RDAs/AIs were calculated for each NHANES cycle. Age-adjusted percentage of the population meeting RDAs/AIs was determined by direct standardization using civilian noninstitutionalized population in 2017-March 2020 NHANES cycle as a reference (aged 20–39 years, 40–59 years, and ≥ 60 years). Subgroup analyses were performed by age (20-34y, 35-49y, 50-64y, ≥ 65y), gender (male, female), race/ethnicity (White, Black, and Hispanic), education level (less than high school graduate, high school or equivalent, and college or above), and family income (ratio of family income to poverty: < 1.30, 1.30–3.49, and ≥ 3.50). NHANES oversampled Mexican persons before 2007 and oversampled all Hispanic persons from 2007 onward [39]. Thus, the Hispanic ethnic group was only analytically assessed from 2007 onward. Besides, trends of other races/ethnicities were not evaluated, because it was hard to calculate reliable estimates for the group across all NHANES cycles [39]. Logistic regression model was used for proportions and linear regression model for means to examine the statistical significance of trends by assigning 2-year survey cycle as a continuous variable. Differences in estimated intake were calculated between 1999–2000 and 2017-March 2020 cycles with adjustment of gender, age, race/ethnicity, education level, and family income. To evaluate potential differences in trends by population subgroups, a survey-weighted Wald F statistic was used to test for an interaction between survey cycle and demographic factors. To minimize the impact caused by demographic shifts, we adjusted gender, age, race/ethnicity, education level, and family income in estimating the trends. Participants with missing data on education (*n* = 56) and family income (*n* = 4230) were excluded in the corresponding subgroup analyses and multivariable analysis.

Three sensitivity analyses were conducted in this study. Firstly, we excluded participants in 1999–2000 and 2001–2002 survey cycle to ensure consistency in methods across all cycles, because only one 24-hour dietary recall has been collected in NHANES 1999–2000 and 2001–2002 survey cycle. Secondly, we excluded participants with CVD, diabetes, or CKD, because these diseases may affect dietary behaviors. Thirdly, we estimated trends in minerals intake from FB and minerals intake by major food sources.

All statistical analyses were performed by R 4.2.1. *P* values were adjusted by the method of Benjamini–Hochberg false discovery rate (FDR) correction, and a two-sided *FDR* < 0.05 is considered to be statistically significant.

## Results

### Participant characteristics

A total of 48,223 U.S. adults older than 20 years were included in this study. Of these, 23,498 men (47.9%), 21,504 White participants (68.9%), and the weighted mean (SE) age was 47.56 (0.19) years (Table 1). From 1999–2000 to 2017-March 2020, the proportion of older adults (aged ≥ 65 years) increased from 17.6% to 22.4%, while the proportion of younger adults (aged 20–34 years) decreased from 29.5% to 27.0%. The proportion of White participants decreased from 71.4% to 62.9%, while the proportion of other races/ethnicities participants increased from 4.6% to 10.0%. The proportion of participants with college or above education increased from 50.0% to 62.4%, while the proportion of participants with less than high school education decreased from 23.8% to 10.1% (all *P* < 0.001 for trend).

**Table 1:** Sociodemographic Characteristics of U.S. Adults ≥ 20 Years by NHANES Survey Cycle, 1999-March 2020 (*N* = 52,726)

	No. of Participants (Weighted%) ^a^										
Characteristics	1999–2000(*n* = 3745)	2001–2002(*n* = 4171)	2003–2004(*n* = 4085)	2005–2006(*n* = 4059)	2007–2008(*n* = 5155)	2009–2010(*n* = 5505)	2011–2012(*n* = 4584)	2013–2014(*n* = 4789)	2015–2016(*n* = 4798)	2017-March 2020 (*n* = 7332)	*P *for Trend
**Gender**											0.911
Male	1808(47.6%)	2046(47.9%)	2037(48.5%)	2057(48.3%)	2513(46.9%)	2640(47.5%)	2280(48.4%)	2281(48.1%)	2310(48.2%)	3526(47.7%)	
Female	1937(52.4%)	2125(52.1%)	2048(51.5%)	2002(51.7%)	2642(53.1%)	2865(52.5%)	2304(51.6%)	2508(51.9%)	2488(51.8%)	3806(52.3%)	
**Age group, y**											< 0.001
20–34	828(29.5%)	967(27.1%)	964(28.0%)	1027(25.8%)	1145(27.0%)	1324(26.7%)	1206(26.6%)	1200(26.7%)	1173(26.9%)	1638(27.0%)	
35–49	935(31.7%)	1135(33.5%)	969(28.6%)	1066(30.3%)	1293(29.8%)	1446(28.7%)	1116(26.3%)	1261(26.3%)	1189(25.0%)	1711(24.3%)	
50–64	882(21.3%)	963(22.6%)	881(24.9%)	938(25.1%)	1353(26.1%)	1377(26.5%)	1249(29.0%)	1257(28.0%)	1256(26.7%)	2123(26.4%)	
≥ 65	1100(17.6%)	1106(16.7%)	1271(18.4%)	1028(18.8%)	1364(17.1%)	1358(18.1%)	1013(18.1%)	1071(19.0%)	1180(21.5%)	1860(22.4%)	
**Race/ethnicity**											< 0.001
Hispanic ^b^	1240(13.6%)	1050(12.6%)	928(10.7%)	901(10.3%)	1442(13.1%)	1575(13.5%)	894(14.2%)	1077(14.8%)	1474(14.9%)	1579(15.7%)	
White	1691(71.4%)	2221(73.1%)	2208(73.3%)	2070(73.4%)	2456(70.8%)	2676(68.9%)	1764(66.9%)	2128(65.8%)	1648(64.8%)	2642(62.9%)	
Black	705(10.5%)	771(10.3%)	792(11.0%)	929(11.2%)	1061(11.0%)	964(11.2%)	1201(11.3%)	931(11.0%)	1002(10.7%)	1943(11.4%)	
Other ^c^	109(4.6%)	129(4.0%)	157(5.0%)	159(5.1%)	196(5.2%)	290(6.4%)	725(7.7%)	653(8.4%)	674(9.5%)	1168(10.0%)	
**Education level **^**d**^											< 0.001
Less than high school graduate	1454(23.8%)	1236(18.6%)	1184(18.1%)	1097(16.6%)	1571(19.9%)	1548(18.6%)	1050(16.2%)	952(14.5%)	1080(13.5%)	1274(10.1%)	
High school or equivalent	843(26.2%)	976(24.1%)	1029(26.5%)	987(25.6%)	1265(25.7%)	1262(22.5%)	961(19.8%)	1081(22.2%)	1059(20.9%)	1766(27.5%)	
College or above	1436(50.0%)	1954(57.3%)	1866(55.4%)	1973(57.8%)	2316(54.4%)	2682(58.9%)	2570(64.0%)	2754(63.3%)	2657(65.7%)	4284(62.4%)	
**Family income**											0.541
Low	955(23.0%)	1022(21.1%)	1080(20.8%)	969(17.0%)	1402(20.9%)	1650(22.0%)	1485(24.7%)	1477(24.4%)	1347(20.5%)	1764(19.1%)	
Intermediate	1235(34.8%)	1522(34.9%)	1572(37.8%)	1534(37.1%)	1832(34.3%)	1891(35.8%)	1461(33.9%)	1536(34.2%)	1752(36.5%)	2507(34.0%)	
High	1018(42.2%)	1343(44.1%)	1215(41.4%)	1381(45.9%)	1458(44.8%)	1458(42.2%)	1297(41.4%)	1432(41.3%)	1248(43.0%)	2147(46.9%)	
**Use of Supplements**											< 0.001
Users	1879(54.7%)	2108(53.6%)	2267(58.3%)	2170(59.4%)	2705(57.0%)	2882(56.6%)	2466(57.3%)	2645(59.7%)	2792(62.6%)	4520(64.8%)	
Nonusers	1866(45.3%)	2063(46.4%)	1818(41.7%)	1889(40.6%)	2450(43.0%)	2623(43.4%)	2118(42.7%)	2144(40.3%)	2006(37.4%)	2812(35.2%)	

*Abbreviations:* *NHANES,* National Health and Nutrition Examination Survey

a Percentages were adjusted for NHANES survey weights

b “Hispanic” includes Mexican American Other Hispanic

c “Other” includes Non-Hispanic Asian and Other Race, including multiracial

d Numbers may not sum to the total number of participants because of missing data (*n* = 56 without education data; *n* = 4233 without family income data)

### Trends in minerals intake from FB + DSs

**Fig. 1:**
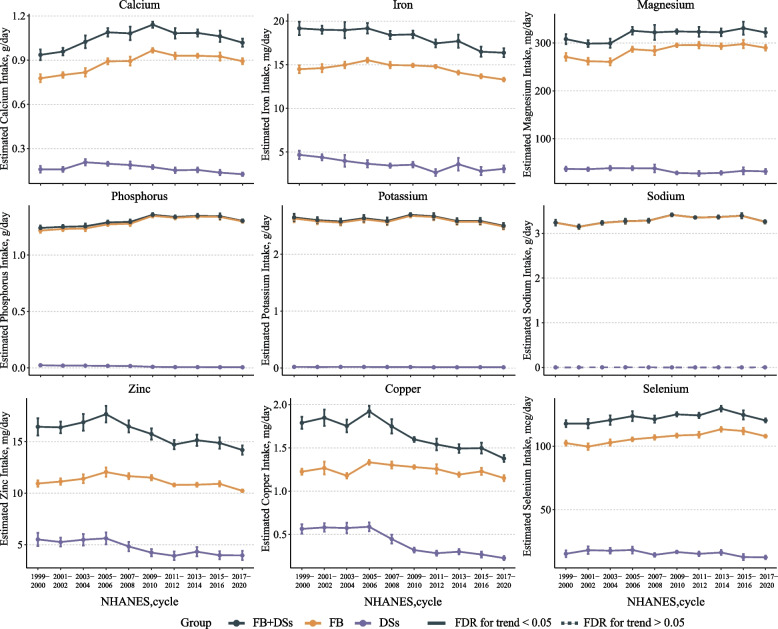
Trends in estimated intake of nine minerals from FB, DSs, and FB + DSs among U.S. adults by NHANES survey cycle, 1999-March 2020. Data were adjusted for NHANES survey weights to be nationally representative. Analyses are based on energy-adjusted values to 2000 kcal/d using the residual method. All estimates were weighted, and error bars indicate 95% CIs. Results were adjusted for gender, age, race/ethnicity, education level, and family income when appropriate. Abbreviations: NHANES, National Health and Nutrition Examination Survey; FB, foods and beverages; DSs, Dietary supplements

From 1999 to March 2020, the estimated intake of calcium (from 0.94 g/day to 1.02 g/day), magnesium (from 308.07 mg/day to 321.85 mg/day), phosphorus (from 1.24 g/day to 1.30 g/day), sodium (from 3.24 g/day to 3.26 g/day), and selenium (from 102.45 mcg/day to 107.87 mcg/day) from FB + DSs significantly increased, while that of iron (from 19.17 mg/day to 16.38 mg/day), potassium (from 2.65 g/day to 2.50 g/day), zinc (from 16.45 mg/day to 14.19 mg/day), and copper (from 1.79 mg/day to 1.38 mg/day) significantly decreased in all participants (all *FDR* < 0.05 for trend). Similar trends were also observed for estimated intake from FB (Fig. 1).

From 1999 to March 2020, the estimated age-adjusted percentage of participants meeting RDAs/AIs for calcium (from 34.37% to 40.87%), phosphorus (from 95.20% to 98.72%), sodium (from 96.06% to 98.81%), and selenium (from 94.01% to 97.27%) significantly increased, that for iron (from 76.45% to 74.90%), potassium (from 36.72% to 25.84%), zinc (from 70.20% to 68.34%), and copper (81.80% to 75.73%) significantly decreased in total participants (Supplementary Fig. 2).

In sensitivity analyses, similar trends were observed when trends were examined from 2003 to March 2020 (Supplementary Table 1), and when participants with diabetes, CVD, or CKD were excluded (Supplementary Figs. 3, 4).

### Trends in minerals intake by source

**Fig. 2:**
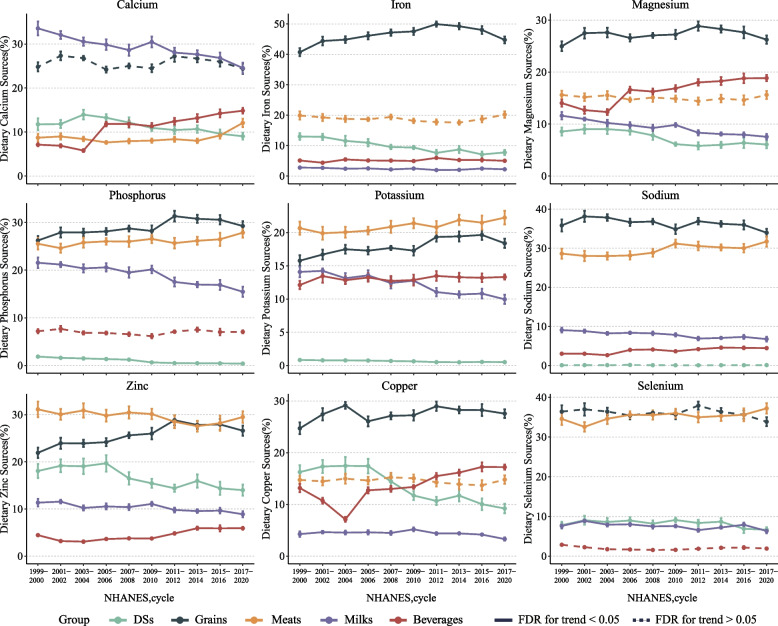
Trends in estimated percentage of nine minerals (from FB + DSs) from selected four food sources and DSs among U.S. adults by NHANES survey cycle, 1999-March 2020. Data were adjusted for NHANES survey weights to be nationally representative. Analyses are based on energy-adjusted values to 2000 kcal/d using the residual method. All estimates were weighted, and error bars indicate 95% CIs. Results were adjusted for gender, age, race/ethnicity, education level, and family income when appropriate. Abbreviations: NHANES, National Health and Nutrition Examination Survey; DSs, Dietary supplements; Milks, Milk and milk products; Meats, Meat, poultry, fish, and mixtures; Grains, Grain products; Beverages, Sugars, sweets, and beverages

From 1999 to March 2020, meats, grains, milks, beverages, and DSs were major sources of minerals (Fig. 2, Supplementary Tables 2, 3). With adjustment of changes in the sociodemographic characteristics in study periods, we found remarkable alterations in dietary components among U.S. adults (Supplementary Fig. 5). Moreover, remarkable alterations were also observed in minerals intake and their sources (Supplementary Tables 2, 3).

From 1999 to March 2020, DSs provided a large proportion of minerals, while DSs-based minerals decreased (Fig. 2, Supplementary Table 2). The estimated percentage of DSs-based zinc decreased from 18.06% to 14.00% (difference, − 5.90%; 95% CI, − 8.03% to − 3.76%; *FDR* < 0.001 for trend), DSs-based copper decreased from 16.27% to 9.22% (difference, − 8.39%; 95% CI, − 10.22% to − 6.56%; *FDR* < 0.001 for trend), DSs-based iron decreased from 13.01% to 7.75% (difference, -5.88%; 95% CI, -7.44% to -4.32%; *FDR* < 0.001 for trend). Similar results were observed among calcium (from 11.75% to 9.04%), magnesium (from 8.57% to 6.07%), phosphorus (from 1.86% to 0.40%), potassium (from 0.83% to 0.51%), and selenium (from 7.81% to 6.67%) (all *FDR* < 0.001 for trend).

In line with decline in milks consumption, the milks-based minerals significantly decreased (Fig. 2, Supplementary Table 2). The estimated percentage of calcium (from 33.56% to 24.55%; difference, -8.92%; 95% CI, -11.24% to -6.59%), phosphorus (from 21.55% to 15.46%; difference, -5.96%; 95% CI, -7.66% to -4.26%), potassium (from 14.08% to 9.96%; difference, -3.92%; 95% CI, -5.06% to -2.77%) from milks significantly decreased (all *FDR* < 0.001 for trend). Similar trends were observed among iron (from 2.77% to 2.22%), magnesium (from 11.61% to 7.55%), sodium (from 9.05% to 6.75%), zinc (from 11.35% to 8.89%), selenium (from 7.62% to 6.34%), and copper (from 4.27% to 3.31%) (all *FDR* < 0.001 for trend).

Though the meat consumption remained stable (Fig. 2, Supplementary Table 2), the estimated percentage of calcium (from 8.72% to 12.07%), phosphorus (from 25.54% to 27.87%), potassium (from 20.67% to 22.25%), sodium (from 28.62% to 31.72%), and selenium (from 34.57% to 37.21%) from meats significantly increased (all *FDR* < 0.05 for trend). In line with increase in whole grain consumption, the estimated percentage of iron (from 40.74% to 44.77%), magnesium (from 24.97% to 26.25%), phosphorus (from 26.22% to 29.26%), potassium (from 15.77% to 18.39%), sodium (from 35.83% to 33.97%), zinc (from 21.92% to 26.60%), and copper (from 24.72% to 27.57%) from grains significantly increased (all *FDR* < 0.05 for trend) (Fig. 2, Supplementary Table 2).

Interestingly, the beverages-based minerals significantly increased though beverages consumption decreased (Fig. 2, Supplementary Table 2). The estimated percentage of calcium (from 7.13% to 14.84%; difference, 8.02%; 95% CI, 7.31% to 8.72%), magnesium (from 14.03% to 18.85%; difference, 5.55%; 95% CI, 4.73% to 6.36%), and copper (from 13.18% to 17.22%; difference, 4.93%; 95% CI, 4.03% to 5.83%) significantly increased (all *FDR* < 0.05 for trend). Similar trends were observed among potassium (from 12.11% to 13.32%), sodium (from 3.01% to 4.45%), and zinc (from 4.46% to 5.92%) (all *FDR* < 0.05 for trend).

Additionally, the minerals from vegetables and fruits decreased over time (Supplementary Fig. 6, Supplementary Table 2). The estimated percentage of magnesium from vegetables (from 12.94% to 10.96%) and fruits (from 6.04% to 5.33%) significantly decreased. The estimated percentage of potassium from vegetables (from 20.55% to 18.62%) and fruits (from 10.68% to 9.31%) significantly decreased. The estimated percentage of copper from vegetables decreased from 14.51% to 11.22%. Similar trends were observed among other minerals. Besides, the estimated percentage of minerals from nuts increased (e.g., from 5.00% to 7.42% for calcium, from 5.21% to 8.45% for copper).

In sensitivity analyses, observed results did not alter materially when trends were examined from 2003 to March 2020 (Supplementary Table 2), and when trends were examined in minerals intake from FB (Supplementary Table 2 and Supplementary Fig. 7), and when participants with diabetes, CVD, and CKD were excluded (Supplementary Fig. 8), and when trends were examined in estimated absolute minerals intake from FB + DSs (Supplementary Table 3).

### Trends in population subgroups

**Fig. 3:**
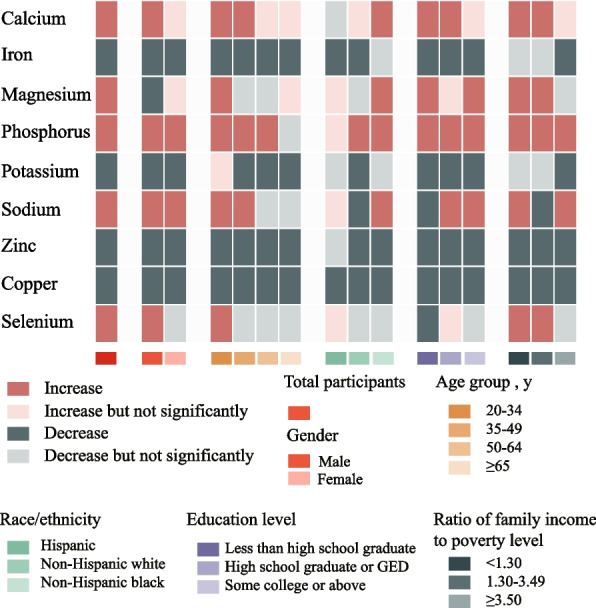
Trends in estimated absolute intake of nine minerals (from FB + DSs) by sociodemographic characteristics, 1999 to March 2020. Data were adjusted for NHANES survey weights to be nationally representative. Analyses are based on energy-adjusted values to 2000 kcal/d using the residual method. Results were adjusted for gender, age, race/ethnicity, education level, and family income when appropriate. Abbreviations: NHANES, National Health and Nutrition Examination Survey; FB, foods and beverages; DSs, Dietary supplements

Divergent trends in minerals intake were observed among population subgroups from 1999 to March 2020 (Fig. 3 and Supplementary Figs. 9–13). Many differences in minerals intake by age, gender, race/ethnicity, education, and income appeared to narrow but did not eliminate over time, such as calcium, iron, zinc, copper, and selenium. For example, time trends in calcium intake were highly variable depending on race/ethnicity (*P*-interaction < 0.001): no change was evident among White and Hispanic participants, and significant increase was seen among Black participants; findings were similar by age, gender, education, and income. Some differences in intake by age, gender, race/ethnicity, education, and income were relatively stable over time, such as magnesium and potassium. For example, the estimated intake of magnesium and potassium were consistently higher among White vs. Black participants, and among higher vs. lower socioeconomic status participants during most of study periods. For sodium and phosphorus, the estimated intake was consistently similar among all population subgroups during the study periods.

**Fig. 4:**
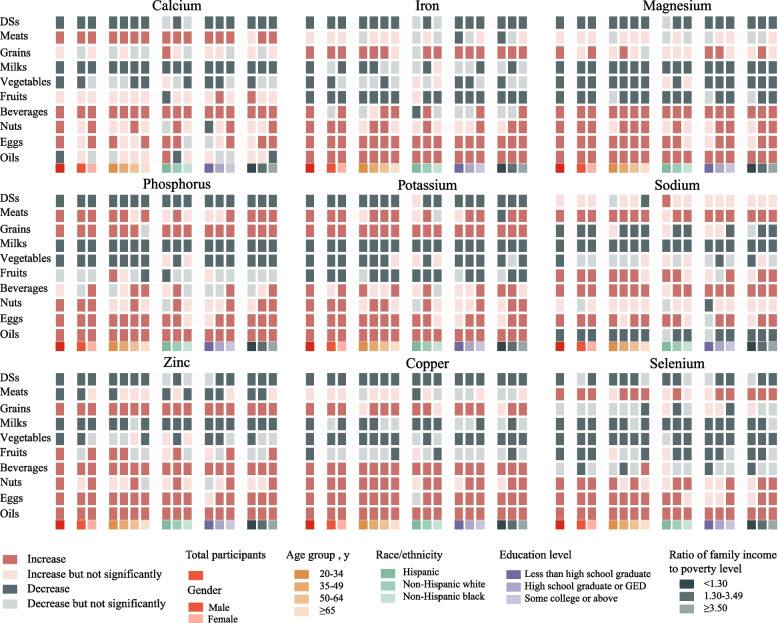
Trends in estimated percentage of nine minerals (from FB + DSs) from nine food groups and DSs by sociodemographic characteristics, 1999 to March 2020. Data were adjusted for NHANES survey weights to be nationally representative. Analyses are based on energy-adjusted values to 2000 kcal/d using the residual method. Results were adjusted for gender, age, race/ethnicity, education level, and family income when appropriate. Abbreviations: NHANES, National Health and Nutrition ExaminationSurvey; DSs, Dietary supplements; Milks, Milk and milk products; Meats, Meat, poultry, fish, and mixtures; Eggs, Eggs; Nuts, Legumes, nuts, and seeds; Grains, Grain products; Fruits, Fruits; Vegetables, Vegetables; Oils, Fats, oils, and salad dressings; Beverages, Sugar, sweeteners, and beverages

Secular trends in minerals intake by food sources by age, gender, race/ethnicity, education level, and income were similar with those in the overall population (Fig. 4, Supplementary Tables 5–13). However, differences by age, gender, race/ethnicity, education, and income appeared to narrow or remained stable over time. For example, the estimated percentage of minerals from DSs were consistently higher among White vs. Black participants during most of study periods, but the decreases in estimated percentage of minerals from DSs were greater among White vs. Black participants. Findings were similar by age, gender, education, and income. The estimated percentage of minerals from meats were consistently higher among White vs. Black, among higher vs. lower socioeconomic status participants during most of the study periods.

Comparative differences in sources and trends among population groups from 2003–2004 to 2017-March 2020 remained similar (Supplementary Tables 4–13). Moreover, results did not alter materially when trends were examined on minerals intake from FB or estimated absolute minerals intake from FB + DSs (Supplementary Tables 4–13).

## Discussion

In this large, nationally representative survey of U.S. adults, both minerals intake and their sources altered significantly among U.S. adults, with increases in calcium, magnesium, phosphorus, and sodium, and decreases in iron, zinc, copper, and potassium. Sociodemographic differences in minerals intake have persisted, but several differences possibly narrowed over time. Disconcertingly, overall minerals intake remained irrational, with high sodium intake greatly exceeding AIs and low magnesium and potassium intake under RDAs/AIs. Moreover, the proportion of adults meeting RDAs for iron, potassium, zinc, and copper significantly decreased.

Freedman MR et al. [40] observed that usual mean intakes of iron, copper, and zinc decreased, that of magnesium and calcium increased, and that of sodium and potassium decreased slightly (male) or remained stable (female). Due to the difference in study periods and methods for estimating minerals intake, it is hard to directly compare our results with previous studies. But our results confirmed previous findings that intake of iron, copper, and zinc decreased, and intake of magnesium and calcium increased in 1999-March 2020 [40]. Moreover, we also observed the percentage of participants meeting RADs of calcium and magnesium increased during the study periods. However, some important challenges in minerals intake remained. Firstly, sodium intake remained increasing or stable across all subgroups in past 21.2 years, however, that greatly exceeded the AIs and chronic diseases Risk Reduction intake [41]. Some commonly consumed processed, packaged, and prepared foods (e.g. deli-meat sandwiches, pizza, pasta dishes, and burgers) were known to contribute significantly to sodium intake. Thus, reducing sodium in these foods may be useful in reducing sodium intake. Secondly, consistent underconsumption of potassium was common among U.S. adults from 1999 to March 2020. Furthermore, potassium intake and the percentage of adults meeting AIs of potassium significantly decreased. Fresh fruits and vegetables provided a range of minerals and vitamins. Unfortunately, there was a huge gap between fruits and vegetables consumption and recommendations for fruits and vegetables among U.S. adults, and the gap appeared to be narrowed hardly during 1999-March 2020 [8, 42]. Therefore, great effort should be taken in encouraging adults to take more fresh fruits and vegetables. Thirdly, the percentage of adults meeting RADs of iron, zinc, and copper decreased, while intake of iron, zinc, and copper was consistently higher than RADs in all participants and subgroups during the study periods. Additionally, intake of phosphorus and selenium increased or stabilized in study periods, however, that was consistently higher than RADs during the study periods. Epidemiologic and experimental studies indicated both nutrients deficiency and excess may associate with an increased risk of adverse health outcomes [43–46]. In addition to minerals deficiency, supranutritional minerals intake also need to pay great cautious.

Socioeconomic status and cultural differences were strongly associated with dietary quality including macronutrients and micronutrients intake. In this study, we found persistent differences in minerals intake among major sociodemographic population groups, whereas these differences were diminishing in the past two decades. White, higher socioeconomic status participants had a higher iron intake, but had a greater decrease in iron intake. Inversely, Black, lower socioeconomic status participants had a lower calcium intake, but had a greater increase in calcium intake. Previous studies reported closing gaps in dietary habits or dietary quality across different socioeconomic, racial or chronic diseases subpopulations, which were consistent with our findings to some extent [42, 47]. There were several potential explanations for the diminishing disparities across different socioeconomic and racial subpopulations. Firstly, some federally-funded food assistance program (such as Supplemental Nutrition Assistance Program) for lower income family may be effective in closing the income-related disparity in minerals intake [48, 49]. Secondly, economic growth and cultural integration over time may also contribute to similar dietary habits or dietary quality across different subpopulations.

Despite observed alterations in minerals intake, significant changes in sources of minerals were also identified over the past two decades. This study found milk consumption and minerals from milk and milk products significantly decreased though the consumption of cheese and yogurt increased. The fact that decreasing milk consumption paralleled with increasing consumption of cheese means greater intake of cholesterol and calories [50]. Previous studies suggested that diagnosis or perception of lactose intolerance was an important reason for decreasing milk consumption. In fact, large misunderstandings of lactose intolerance may exist among a significant percentage of individuals [50, 51]. Milk, widely available and rich in many micronutrients, was a low-cost source of minerals. Thus, necessary and effective measure should be taken to alleviate unnecessary avoidance of milk and excessive consumption of cheese. Besides, consumption of vegetables and vegetables-based minerals also decreased during the periods. Vegetables were excellent sources of potassium, magnesium, and vitamins, though oxalic acid in vegetables may weaken the absorption of dietary calcium and iron. Dietary calcium, magnesium, zinc, and copper based on beverages significantly increased though beverages consumption has declined [52], indicating the consumption pattern of beverages may undergone a butterfly change. Beyond the alteration in their food sources, DSs-based minerals decreased dramatically, which was consistent with declines in DSs use in America [28, 29]. Evolving evidence about limited effect of supplements in preventing chronic diseases in mid-to-late 2000s may have contributed to this trend [53–55]. Additionally, several expert bodies also declared there was insufficient evidence to prove the presence or absence of benefits of DSs in preventing cancer and chronic diseases [56, 57]. Lastly, the economic downturn in the late 20th and early twenty-first centuries may have also impact on these trends.

Of note, differences between daily nutrients intake and recommended intake of DRIs were approximatively negligible, yet such puny differences in daily intake may sum to earthshaking differences in weekly, monthly, or annual consumption [58]. In addition, small mean changes among entire population or specific subgroups may cause substantial impacts on their overall exposure distribution and corresponding risk [59]. Consistent with this, the modest changes in individual minerals intake may lead to meaningful changes in the prevalence of certain diseases. However, the prevalence of certain diseases paralleled with trends in dietary minerals intake have been barely noticed, despite the paralleled trends in specific categories of food have been focused (e.g., the association between consumption trend of sugar-sweetened beverages and ultra-processed foods and prevalence of childhood overweight and obesity). Given the importance of minerals for health, more efforts must be devoted to improving minerals intake.

This study has several strengths. The study was conducted using the most recent data available in NHANES, providing generalizability to U.S. adults. Moreover, the study thoroughly investigated the trend of minerals intake based on quantity and sources in general population and multiple subgroups, providing guidance for a rational and scientific diet. However, some limitations existing in this study need to be considered. Firstly, the minerals intake and sources were calculated based on 24-h recalls, which could be susceptible to random and systematic error. Secondly, the study used nine USDA food groups, which may miss out on the unique nutritional benefits of each individual food. Thirdly, the study focused on total mineral and food group-based minerals intake, and ignored the effect of processing methods and food combination on mineral absorption and bioavailability. Fourthly, because only 9 minerals were provided in The Total Nutrient Intakes files of NHANES, we only evaluate trends in the nine minerals intake and sources among U.S. adults. Thus, further studies need to investigate trends in other minerals intake and sources among U.S. adults. Lastly, we analyzed trends using linear regression, though the data may not be linear.

## Conclusion

From 1999 to March 2020, both minerals intake and their sources experienced a significant alteration among U.S. adults. Additionally, trends in minerals intake and their sources varied by population subgroups, and many differences appeared to narrow over time. Although some improvements were observed, important challenges, such as overconsumption of sodium and underconsumption of potassium, calcium, and magnesium, still remained among U.S. adults.

### Supplementary Information

**Additional file 1:** **Supplementary Table 1.** Changes in Estimate Intake of Nine Minerals Among U.S. Adults by NHANES Survey Cycle, 1999-2020. **Supplementary Table 2.** Changes in Estimated Percentage of Nine Minerals Intake from Nine Food Groups and Dietary. Supplements Among U.S. Adults by NHANES Survey Cycle, 1999-2020. **Supplementary Table 3.** Trends in Estimated Absolute Intake of Nine Minerals from Nine Food Groups and Dietary Supplements by NHANES Survey Cycle, 1999-2020. **Supplementary Table 4.** Changes in Estimate Intake of Nine Minerals by Sociodemographic Characteristics by NHANES Survey Cycle, 1999-2020. **Supplementary Figure 1.** Participant flow chart. **Supplementary Figure 2.** Trends in Age-adjusted Percentage of U.S. Adults Meeting RDAs/AIs by NHANES Survey Cycle, 1999-2020. **Supplementary Figure 3. **Trends in Estimated Intake of Nine Minerals From FB, DSs, and FB + DSs After Excluding Participants with Diabetes, CVD, and CKD Among U.S. Adults, 1999-2020. **Supplementary Figure 4.** Trends in Age-adjusted Percentage of U.S. Adults Meeting RDAs/AIs After Excluding Participants with Diabetes, CVD, and CKD, 1999-2020. **Supplementary Figure 5.** Changes in Estimated Consumption of Dietary Components Among U.S. Adults, 1999-2020. **Supplementary Figure 6.** Trends in Estimated Percentage of Nine Minerals (from FB + DSs) From Selected Five Food Sources Among U.S. Adults, 1999-2020. **Supplementary Figure 7.** Trends in Estimated Percentage of Nine Minerals (from FB) From Nine Food Sources Among U.S. Adults, 1999-2020. **Supplementary Figure 8.** Trends in Estimated Percentage of Nine Minerals (from FB + DSs) From Nine Food Sources and DSs Among U.S. Adults after excluding participants with diabetes, CVD, and CKD, 1999-2020. **Supplementary Figure 9.** Trends in Estimated Absolute Intake of Nine Minerals from FB + DSs by Gender, 1999-2020. **Supplementary Figure 10.** Trends in Estimated Absolute Intake of Nine Minerals from FB + DSs by Age Group, 1999-2020. **Supplementary Figure 11.** Trends in Estimated Absolute Intake of Nine Minerals from FB + DSs by Race/ethnicity, 1999-2020. **Supplementary Figure 12.** Trends in Estimated Absolute Intake of Nine Minerals from FB + DSs by Education Level, 1999-2020. **Supplementary Figure 13.** Trends in Estimated Absolute Intake of Nine Minerals from FB + DSs by Family Income, 1999-2020

**Additional file 2:** **Supplementary Table 5.** Trends in Estimated Percent/Absolute Intake of Calcium From Nine Food Groups and Dietary Supplements by Sociodemographic Characteristics, 1999-2020. **Supplementary Table 6.** Trends in Estimated Percent/Absolute Intake of Iron From Nine Food Groups and Dietary Supplements by Sociodemographic Characteristics, 1999-2020. **Supplementary Table 7.** Trends in Estimated Percent/Absolute Intake of Magnesium From Nine Food Groups and Dietary Supplements by Sociodemographic Characteristics, 1999-2020. **Supplementary Table 8.** Trends in Estimated Percent/Absolute Intake of Phosphorus From Nine Food Groups and Dietary Supplements by Sociodemographic Characteristics, 1999-2020. **Supplementary Table 9.** Trends in Estimated Percent/Absolute Intake of Potassium From Nine Food Groups and Dietary Supplements by Sociodemographic Characteristics, 1999-2020. **Supplementary Table 10.** Trends in Estimated Percent/Absolute Intake of Sodium From Nine Food Groups and Dietary Supplements by Sociodemographic Characteristics, 1999-2020. **Supplementary Table 11.** Trends in Estimated Percent/Absolute Intake of Zinc From Nine Food Groups and Dietary Supplements by Sociodemographic Characteristics, 1999-2020. **Supplementary Table 12.** Trends in Estimated Percent/Absolute Intake of Copper From Nine Food Groups and Dietary Supplements by Sociodemographic Characteristics, 1999-2020. **Supplementary Table 13.** Trends in Estimated Percent/Absolute Intake of Selenium From Nine Food Groups and Dietary Supplements by Sociodemographic Characteristics, 1999-2020.

## Data Availability

Publicly available datasets were analyzed in this study. This data can be found here: https://wwwn.cdc.gov/nchs/nhanes/Default.aspx

## Abbreviations

AIs: Adequate Intakes
CKD: Chronic kidney disease
CVD: Cardiovascular disease
DSs: Dietary supplements
DRIs: Dietary Reference Intakes
eGFR: Estimated glomerular filtration rate
FB: Foods and beverages
FDR: False discovery rate
NHANES: National Health and Nutrition Examination Survey
RDAs: Recommended Dietary Allowances
U.S.: The United States
USDA: The United States Department of Agriculture

## References

[1] Micha R, Penalvo JL, Cudhea F, Imamura F, Rehm CD, Mozaffarian D (2017). Association Between Dietary Factors and Mortality From Heart Disease, Stroke, and Type 2 Diabetes in the United States. JAMA.

[2] Collaborators GBDD (2019). Health effects of dietary risks in 195 countries, 1990–2017: a systematic analysis for the Global Burden of Disease Study 2017. Lancet.

[3] Collaborators USBoD, Mokdad AH, Ballestros K, Echko M, Glenn S, Olsen HE, Mullany E, Lee A, Khan AR, Ahmadi A, et al: The State of US Health, 1990–2016: Burden of Diseases, Injuries, and Risk Factors Among US States. JAMA 2018, 319:1444–1472 10.1001/jama.2018.0158 10.1001/jama.2018.0158 PMC5933332 29634829

[4] Jardim TV, Mozaffarian D, Abrahams-Gessel S, Sy S, Lee Y, Liu J, Huang Y, Rehm C, Wilde P, Micha R, Gaziano TA (2019). Cardiometabolic disease costs associated with suboptimal diet in the United States: A cost analysis based on a microsimulation model. PLoS Med.

[5] Virani SS, Alonso A, Aparicio HJ, Benjamin EJ, Bittencourt MS, Callaway CW, Carson AP, Chamberlain AM, Cheng S, Delling FN (2021). Heart Disease and Stroke Statistics-2021 Update: A Report From the American Heart Association. Circulation.

[6] Shah NS, Lloyd-Jones DM, O'Flaherty M, Capewell S, Kershaw KN, Carnethon M, Khan SS (2019). Trends in Cardiometabolic Mortality in the United States, 1999–2017. JAMA.

[7] Wang L, Li X, Wang Z, Bancks MP, Carnethon MR, Greenland P, Feng YQ, Wang H, Zhong VW (2021). Trends in Prevalence of Diabetes and Control of Risk Factors in Diabetes Among US Adults, 1999–2018. JAMA.

[8] Shan Z, Rehm CD, Rogers G, Ruan M, Wang DD, Hu FB, Mozaffarian D, Zhang FF, Bhupathiraju SN (2019). Trends in Dietary Carbohydrate, Protein, and Fat Intake and Diet Quality Among US Adults, 1999–2016. JAMA.

[9] Wang DD, Leung CW, Li Y, Ding EL, Chiuve SE, Hu FB, Willett WC (2014). Trends in dietary quality among adults in the United States, 1999 through 2010. JAMA Intern Med.

[10] Liu J, Micha R, Li Y, Mozaffarian D (2021). Trends in Food Sources and Diet Quality Among US Children and Adults, 2003–2018. JAMA Netw Open.

[11] Sale C, Elliott-Sale KJ (2019). Nutrition and Athlete Bone Health. Sports Med.

[12] Yao Y, Chen Z, Zhang H, Chen C, Zeng M, Yunis J, Wei Y, Wan Y, Wang N, Zhou M (2021). Selenium-GPX4 axis protects follicular helper T cells from ferroptosis. Nat Immunol.

[13] Orlich MJ, Mashchak AD, Jaceldo-Siegl K, Utt JT, Knutsen SF, Sveen LE, Fraser GE (2022). Dairy foods, calcium intakes, and risk of incident prostate cancer in Adventist Health Study-2. Am J Clin Nutr.

[14] Roemhild K, von Maltzahn F, Weiskirchen R, Knuchel R, von Stillfried S, Lammers T (2021). Iron metabolism: pathophysiology and pharmacology. Trends Pharmacol Sci.

[15] Hubner C, Haase H (2021). Interactions of zinc- and redox-signaling pathways. Redox Biol.

[16] Pasricha SR, Tye-Din J, Muckenthaler MU, Swinkels DW (2021). Iron deficiency. Lancet.

[17] Wessels I, Fischer HJ, Rink L (2021). Dietary and Physiological Effects of Zinc on the Immune System. Annu Rev Nutr.

[18] Vinceti M, Filippini T, Rothman KJ (2018). Selenium exposure and the risk of type 2 diabetes: a systematic review and meta-analysis. Eur J Epidemiol.

[19] Walker S, Baum JI (2022). Eggs as an affordable source of nutrients for adults and children living in food-insecure environments. Nutr Rev.

[20] Hossain MAM, Uddin SMK, Sultana S, Wahab YA, Sagadevan S, Johan MR, Ali ME (2022). Authentication of Halal and Kosher meat and meat products: Analytical approaches, current progresses and future prospects. Crit Rev Food Sci Nutr.

[21] Filho AM, Pirozi MR, Borges JT, Pinheiro Sant'Ana HM, Chaves JB, Coimbra JS (2017). Quinoa: Nutritional, functional, and antinutritional aspects. Crit Rev Food Sci Nutr.

[22] McCormick R, Sim M, Dawson B, Peeling P (2020). Refining Treatment Strategies for Iron Deficient Athletes. Sports Med.

[23] Narasaki Y, You AS, Malik S, Moore LW, Bross R, Cervantes MK, Daza A, Kovesdy CP, Nguyen DV, Kalantar-Zadeh K, Rhee CM (2022). Dietary potassium intake, kidney function, and survival in a nationally representative cohort. Am J Clin Nutr.

[24] Woo HW, Lim YH, Kim MK, Shin J, Lee YH, Shin DH, Shin MH, Choi BY (2020). Prospective associations between total, animal, and vegetable calcium intake and metabolic syndrome in adults aged 40 years and older. Clin Nutr.

[25] Jo G, Park D, Lee J, Kim R, Subramanian SV, Oh H, Shin MJ (2022). Trends in Diet Quality and Cardiometabolic Risk Factors Among Korean Adults, 2007–2018. JAMA Netw Open.

[26] Rehm CD, Penalvo JL, Afshin A, Mozaffarian D (2016). Dietary Intake Among US Adults, 1999–2012. JAMA.

[27] Cowan AE, Tooze JA, Gahche JJ, Eicher-Miller HA, Guenther PM, Dwyer JT, Potischman N, Bhadra A, Carroll RJ, Bailey RL (2022). Trends in Overall and Micronutrient-Containing Dietary Supplement Use in US Adults and Children, NHANES 2007–2018. J Nutr.

[28] Kantor ED, Rehm CD, Du M, White E, Giovannucci EL (2016). Trends in Dietary Supplement Use Among US Adults From 1999–2012. JAMA.

[29] Wang L, Martinez Steele E, Du M, Pomeranz JL, O'Connor LE, Herrick KA, Luo H, Zhang X, Mozaffarian D, Zhang FF (2021). Trends in Consumption of Ultraprocessed Foods Among US Youths Aged 2–19 Years, 1999–2018. JAMA.

[30] Kit BK, Fakhouri TH, Park S, Nielsen SJ, Ogden CL (2013). Trends in sugar-sweetened beverage consumption among youth and adults in the United States: 1999–2010. Am J Clin Nutr.

[31] McClure ST, Chang AR, Selvin E, Rebholz CM, Appel LJ: Dietary Sources of Phosphorus among Adults in the United States: Results from NHANES 2001–2014. Nutrients 2017, 9,10.3390/nu9020095 10.3390/nu9020095 PMC5331526 28146091

[32] Liu J, Huang Y, Dai Q, Fulda KG, Chen S, Tao MH: Trends in Magnesium Intake among Hispanic Adults, the National Health and Nutrition Examination Survey (NHANES) 1999–2014. Nutrients 2019, 11,10.3390/nu11122867 10.3390/nu11122867 PMC6950381 31766698

[33] Clarke LS, Overwyk K, Bates M, Park S, Gillespie C, Cogswell ME: Temporal Trends in Dietary Sodium Intake Among Adults Aged >/=19 Years - United States, 2003–2016. MMWR Morb Mortal Wkly Rep 2021, 70:1478–1482,10.15585/mmwr.mm7042a4 10.15585/mmwr.mm7042a4 PMC9361836 34673747

[34] CDC. National Health and Nutrition Survey—overview. Hyattsville, MD: CDC, National Center for Health Statistics (NCHS), last updated 2017

[35] Chen F, Du M, Blumberg JB, Ho Chui KK, Ruan M, Rogers G, Shan Z, Zeng L, Zhang FF: Association Among Dietary Supplement Use, Nutrient Intake, and Mortality Among U.S. Adults: A Cohort Study. Ann Intern Med 2019, 170:604–613,10.7326/M18-2478 10.7326/M18-2478 PMC6736694 30959527

[36] Trumbo P, Yates AA, Schlicker S, Poos M (2001). Dietary reference intakes: vitamin A, vitamin K, arsenic, boron, chromium, copper, iodine, iron, manganese, molybdenum, nickel, silicon, vanadium, and zinc. J Am Diet Assoc.

[37] National Academies of Sciences, Engineering, and Medicine; Health and Medicine Division; Food and Nutrition Board; Committee to Review the Dietary Reference Intakes for Sodium and Potassium, Oria M, Harrison M, Stallings VA, eds. Dietary Reference Intakes for Sodium and Potassium. Washington (DC): National Academies Press (US); March 5, 2019. 30844154

[38] Levey AS, Stevens LA, Schmid CH, Zhang YL, Castro AF, Feldman HI, Kusek JW, Eggers P, Van Lente F, Greene T, Coresh J (2009). A new equation to estimate glomerular filtration rate. Ann Intern Med.

[39] Johnson CL, Paulose-Ram R, Ogden CL, Carroll MD, Kruszon-Moran D, Dohrmann SM (2013). National Health and Nutrition Examination Survey: analytic guidelines, 1999–2010. Vital Health Stat.

[40] Freedman MR, Fulgoni VL, Lieberman HR: Temporal changes in micronutrient intake among United States Adults, NHANES 2003 through 2018: A cross-sectional study. Am J Clin Nutr 2024,10.1016/j.ajcnut.2024.02.007 10.1016/j.ajcnut.2024.02.007 38373695

[41] In Dietary Reference Intakes for Sodium and Potassium. Edited by Oria M, Harrison M, Stallings VA. Washington (DC)2019: The National Academies Collection: Reports funded by National Institutes of Health]. 30844154

[42] Yin J, Huang Y, Liu G, Wang L, Shan Z, Liu L (2022). Trends in dietary macronutrient composition and diet quality among US adults with diagnosed and undiagnosed elevated glycemic status: a serial cross-sectional study. Am J Clin Nutr.

[43] Wang X, Fang X, Zheng W, Zhou J, Song Z, Xu M, Min J, Wang F (2021). Genetic Support of A Causal Relationship Between Iron Status and Type 2 Diabetes: A Mendelian Randomization Study. J Clin Endocrinol Metab.

[44] Wang W, Jing X, Du T, Ren J, Liu X, Chen F, Shao Y, Sun S, Yang G, Cui X (2022). Iron overload promotes intervertebral disc degeneration via inducing oxidative stress and ferroptosis in endplate chondrocytes. Free Radic Biol Med.

[45] Bleys J, Navas-Acien A, Guallar E (2008). Serum selenium levels and all-cause, cancer, and cardiovascular mortality among US adults. Arch Intern Med.

[46] Xu ZJ, Liu M, Niu QJ, Huang YX, Zhao L, Lei XG, Sun LH (2023). Both selenium deficiency and excess impair male reproductive system via inducing oxidative stress-activated PI3K/AKT-mediated apoptosis and cell proliferation signaling in testis of mice. Free Radic Biol Med.

[47] Sijtsma FP, Meyer KA, Steffen LM, Shikany JM, Van Horn L, Harnack L, Kromhout D, Jacobs DR (2012). Longitudinal trends in diet and effects of sex, race, and education on dietary quality score change: the Coronary Artery Risk Development in Young Adults study. Am J Clin Nutr.

[48] U.S. Department of Agriculture National Institute of Food and Agriculture Gus Schumacher Nutrition Incentive Program. [(accessed on 26 July 2021)]; Available from: https://nifa.usda.gov/program/gus-schumacher-nutrition-incentive-grant-program.

[49] Qin Y, Cowan AE, Bailey RL, Jun S, Eicher-Miller HA (2023). Usual nutrient intakes and diet quality among United States older adults participating in the Supplemental Nutrition Assistance Program compared with income-eligible nonparticipants. Am J Clin Nutr.

[50] Zingone F, Bucci C, Iovino P, Ciacci C (2017). Consumption of milk and dairy products: Facts and figures. Nutrition.

[51] Vernia P, Di Camillo M, Foglietta T, Avallone VE, De Carolis A (2010). Diagnosis of lactose intolerance and the "nocebo" effect: the role of negative expectations. Dig Liver Dis.

[52] Dai J, Soto MJ, Dunn CG, Bleich SN (2021). Trends and patterns in sugar-sweetened beverage consumption among children and adults by race and/or ethnicity, 2003–2018. Public Health Nutr.

[53] Goodman GE, Thornquist MD, Balmes J, Cullen MR, Meyskens FL, Omenn GS, Valanis B, Williams JH (2004). The Beta-Carotene and Retinol Efficacy Trial: incidence of lung cancer and cardiovascular disease mortality during 6-year follow-up after stopping beta-carotene and retinol supplements. J Natl Cancer Inst.

[54] Bérubé S, Diorio C, Brisson J (2008). Multivitamin-multimineral supplement use and mammographic breast density. Am J Clin Nutr.

[55] Lippman SM, Klein EA, Goodman PJ, Lucia MS, Thompson IM, Ford LG, Parnes HL, Minasian LM, Gaziano JM, Hartline JA (2009). Effect of selenium and vitamin E on risk of prostate cancer and other cancers: the Selenium and Vitamin E Cancer Prevention Trial (SELECT). JAMA.

[56] Huang HY, Caballero B, Chang S, Alberg AJ, Semba RD, Schneyer CR, Wilson RF, Cheng TY, Vassy J, Prokopowicz G (2006). The efficacy and safety of multivitamin and mineral supplement use to prevent cancer and chronic disease in adults: a systematic review for a National Institutes of Health state-of-the-science conference. Ann Intern Med.

[57] Marra MV, Boyar AP (2009). Position of the American Dietetic Association: nutrient supplementation. J Am Diet Assoc.

[58] Liu J, Rehm CD, Onopa J, Mozaffarian D (2020). Trends in Diet Quality Among Youth in the United States, 1999–2016. JAMA.

[59] Rose G (1985). Sick individuals and sick populations. Int J Epidemiol.

