# Supplementary material for: National trends in nine key minerals intake (quantity and source) among U.S. adults, 1999 to march 2020
Source: Nutr J. 2024 May 17;23:52. doi: 10.1186/s12937-024-00950-4 (PMC11100034; doi:10.1186/s12937-024-00950-4)

**Supplementary Information for**

National Trends in Nine Key Minerals Intake (Quantity and Source) Among U.S. Adults, 1999 to March 2020

Xuemin Yan^a^, Xuanyang Wang^a^, Jia Zhang^a^, Zhu Ming^a^, Can Zhang^a^, Pingnan Ma^a^, Qianmin Liu^a^, Yuanyuan Xu^a^, Licheng Cheng^a^, Xibo Pang^a^, Ying Li^a^

^a^Department of Nutrition and Food Hygiene, the National Key Discipline, School of Public Health, Harbin Medical University, Harbin, P. R. China.

**^*^**Correspondence: Ying Li, Department of Nutrition and Food Hygiene, the National Key Discipline, School of Public Health, Harbin Medical University, 157 Baojian Road, Harbin, P. R. China 150081, Phone: 86-451-87502681, E-mail: [liying_helen@163.com](mailto:liying_helen@163.com)

**Supplementary Table 1.** Changes in Estimate Intake of Nine Minerals Among U.S. Adults by NHANES Survey Cycle, 1999-2020

|  | Survey-Weighted Mean (95% CI) ^a^ | | 1999-2020 | | 2003-2020 | |
| --- | --- | --- | --- | --- | --- | --- |
|  | 1999-2000(n=3745) | 2017-2020 (n=7332) | FDR for Trend ^b^ | Difference (95% CI) ^c^ | FDR for Trend ^b^ | Difference (95% CI) ^d^ |
| **FB ^e^ + DSs** | |  |  |  |  |  |
| Calcium (g) | 0.94(0.90, 0.97) | 1.02(0.99, 1.05) | <0.001 | 0.07(0.03, 0.12) | 0.003 | -0.01(-0.05, 0.03) |
| Iron (mg) | 19.17(18.41, 19.94) | 16.38(15.88, 16.87) | <0.001 | -3.29(-4.31, -2.28) | <0.001 | -2.91(-3.97, -1.85) |
| Magnesium (mg) | 308.07(297.71, 318.44) | 321.85(313.18, 330.53) | 0.001 | 2.74(-10.07, 15.55) | 0.856 | 15.03(4.46, 25.59) |
| Phosphorus (g) | 1.24(1.22, 1.26) | 1.30(1.29, 1.32) | <0.001 | 0.05(0.03, 0.08) | <0.001 | 0.04(0.02, 0.07) |
| Potassium (g) | 2.65(2.58, 2.71) | 2.50(2.45, 2.55) | <0.001 | -0.21(-0.28, -0.13) | <0.001 | -0.11(-0.17, -0.05) |
| Sodium (g) | 3.24(3.18, 3.30) | 3.26(3.22, 3.30) | <0.001 | 0.01(-0.08, 0.10) | 0.915 | 0.01(-0.05, 0.07) |
| Zinc (mg) | 16.45(15.61, 17.28) | 14.19(13.74, 14.64) | <0.001 | -2.78(-3.76, -1.81) | <0.001 | -3.04(-3.83, -2.25) |
| Copper (mg) | 1.79(1.72, 1.86) | 1.38(1.34, 1.42) | <0.001 | -0.49(-0.56, -0.41) | <0.001 | -0.43(-0.50, -0.35) |
| Selenium (mcg) | 117.73(115.03, 120.43) | 120.31(118.76, 121.86) | 0.034 | -0.18(-3.65, 3.28) | 0.915 | -2.05(-5.48, 1.38) |
| **FB ^e^** | |  |  |  |  |  |
| Calcium (g) | 0.78(0.75, 0.80) | 0.89(0.87, 0.91) | <0.001 | 0.12(0.09, 0.15) | <0.001 | 0.09(0.05, 0.12) |
| Iron (mg) | 14.50(14.04, 14.95) | 13.30(13.10, 13.51) | <0.001 | -1.35(-1.87, -0.84) | <0.001 | -1.76(-2.17, -1.34) |
| Magnesium (mg) | 270.94(262.95, 278.94) | 289.88(284.39, 295.37) | <0.001 | 12.34(3.13, 21.55) | 0.012 | 23.52(16.07, 30.97) |
| Phosphorus (g) | 1.22(1.20, 1.24) | 1.30(1.29, 1.31) | <0.001 | 0.07(0.05, 0.10) | <0.001 | 0.06(0.03, 0.08) |
| Potassium (g) | 2.63(2.56, 2.69) | 2.49(2.44, 2.54) | <0.001 | -0.20(-0.27, -0.12) | <0.001 | -0.10(-0.16, -0.04) |
| Sodium (g) | 3.24(3.17, 3.30) | 3.26(3.22, 3.29) | <0.001 | 0.01(-0.08, 0.10) | 0.833 | 0.01(-0.05, 0.07) |
| Zinc (mg) | 10.94(10.64, 11.23) | 10.23(10.12, 10.34) | <0.001 | -0.70(-1.05, -0.35) | 0.001 | -1.23(-1.68, -0.78) |
| Copper (mg) | 1.23(1.19, 1.26) | 1.15(1.12, 1.18) | <0.001 | -0.10(-0.15, -0.06) | <0.001 | -0.06(-0.10, -0.01) |
| Selenium (mcg) | 102.45(100.47, 104.43) | 107.87(106.75, 108.99) | <0.001 | 4.30(1.84, 6.76) | 0.002 | 3.92(1.55, 6.30) |

Abbreviations: NHANES, National Health and Nutrition Examination Survey; DSs, Dietary supplements; FB, foods and beverages.

a Data were adjusted for NHANES survey weights.

b Results were adjusted for gender, age, race/ethnicity, education level, and family income.

c 1999-2000 vs. 2017-2020, and differences were adjusted for gender, age, race/ethnicity, education level, and family income.

d 2003-2004 vs. 2017-2020, and differences were adjusted for gender, age, race/ethnicity, education level, and family income.

e Data were adjusted for total energy intake to 2000 kcal/d using the residual method.

**Supplementary Table 2.** Changes in Estimated Percentage of Nine Minerals Intake from Nine Food Groups and Dietary Supplements Among U.S. Adults by NHANES Survey Cycle, 1999-2020

|  | Survey-Weighted Mean (95% CI) ^a^ | | 1999-2020 | | 2003-2020 | |
| --- | --- | --- | --- | --- | --- | --- |
|  | 1999-2000 (n=3745) | 2017-2020 (n=7332) | FDR for Trend ^b^ | Difference (95% CI) ^c^ | FDR for Trend ^b^ | Difference (95% CI) ^d^ |
| **FB + DSs** |  |  |  |  |  |  |
| **Calcium** |  |  |  |  |  |  |
| DS (%) | 11.75(10.42, 13.09) | 9.04(8.27, 9.82) | < 0.001 | -3.85(-5.41, -2.29) | < 0.001 | -5.75(-6.92, -4.58) |
| Meats (%) | 8.72(7.90, 9.54) | 12.07(11.12, 13.03) | < 0.001 | 4.02(2.69, 5.35) | < 0.001 | 4.20(3.04, 5.36) |
| Grains (%) | 24.82(23.81, 25.84) | 24.39(23.20, 25.59) | 0.687 | 0.17(-1.51, 1.84) | 0.599 | -2.01(-3.47, -0.54) |
| Milks (%) | 33.56(31.99, 35.13) | 24.55(23.35, 25.75) | < 0.001 | -8.92(-11.24, -6.59) | < 0.001 | -5.66(-7.29, -4.03) |
| Vegetables (%) | 7.03(6.47, 7.60) | 6.09(5.63, 6.56) | 0.006 | -1.22(-2.13, -0.31) | 0.316 | 0.17(-0.51, 0.85) |
| Fruits (%) | 2.18(1.86, 2.50) | 3.05(2.82, 3.28) | 0.179 | 0.70(0.28, 1.13) | < 0.001 | -0.99(-1.48, -0.49) |
| Beverages (%) | 7.13(6.80, 7.45) | 14.84(14.21, 15.47) | < 0.001 | 8.02(7.31, 8.72) | < 0.001 | 9.07(8.37, 9.77) |
| Nuts (%) | 2.02(1.78, 2.26) | 2.60(2.31, 2.89) | 0.002 | 0.48(0.14, 0.83) | 0.001 | 0.54(0.22, 0.87) |
| Eggs (%) | 2.35(2.03, 2.67) | 2.96(2.69, 3.23) | 0.001 | 0.63(0.14, 1.12) | < 0.001 | 0.39(0.02, 0.76) |
| Oils (%) | 0.43(0.36, 0.49) | 0.41(0.39, 0.43) | 0.047 | -0.02(-0.11, 0.06) | 0.014 | 0.03(-0.02, 0.09) |
| **Iron** |  |  |  |  |  |  |
| DS (%) | 13.01(11.95, 14.06) | 7.75(6.93, 8.57) | < 0.001 | -5.88(-7.44, -4.32) | < 0.001 | -4.40(-6.31, -2.49) |
| Meats (%) | 19.91(18.58, 21.24) | 20.20(19.14, 21.25) | 0.548 | 0.99(-0.83, 2.82) | 0.031 | 2.00(0.54, 3.45) |
| Grains (%) | 40.74(39.45, 42.03) | 44.77(43.63, 45.90) | < 0.001 | 4.44(2.52, 6.37) | 0.087 | 0.50(-1.10, 2.11) |
| Milks (%) | 2.77(2.42, 3.12) | 2.22(2.03, 2.40) | < 0.001 | -0.57(-1.02, -0.12) | 0.237 | -0.20(-0.53, 0.14) |
| Vegetables (%) | 9.34(8.75, 9.92) | 8.33(7.97, 8.69) | 0.046 | -1.23(-2.06, -0.40) | 0.081 | 0.59(0.04, 1.15) |
| Fruits (%) | 3.19(2.77, 3.60) | 2.23(2.04, 2.41) | < 0.001 | -1.23(-1.71, -0.75) | < 0.001 | -0.81(-1.22, -0.40) |
| Beverages (%) | 5.10(4.83, 5.37) | 5.01(4.73, 5.30) | 0.001 | 0.07(-0.35, 0.49) | 0.532 | -0.32(-0.87, 0.23) |
| Nuts (%) | 3.61(3.22, 4.00) | 5.00(4.52, 5.48) | < 0.001 | 1.30(0.70, 1.91) | < 0.001 | 1.05(0.46, 1.64) |
| Eggs (%) | 2.10(1.90, 2.31) | 4.02(3.64, 4.40) | < 0.001 | 1.87(1.33, 2.40) | < 0.001 | 1.38(0.87, 1.88) |
| Oils (%) | 0.25(0.22, 0.28) | 0.49(0.45, 0.54) | < 0.001 | 0.23(0.17, 0.29) | < 0.001 | 0.22(0.16, 0.27) |
| **Magnesium** |  |  |  |  |  |  |
| DS (%) | 8.57(7.79, 9.34) | 6.07(5.36, 6.78) | < 0.001 | -3.35(-4.49, -2.22) | < 0.001 | -3.42(-4.48, -2.35) |
| Meats (%) | 15.60(14.82, 16.39) | 15.61(14.85, 16.37) | 0.859 | 0.47(-0.74, 1.69) | 0.400 | 0.41(-0.61, 1.44) |
| Grains (%) | 24.97(24.02, 25.93) | 26.25(25.45, 27.04) | 0.015 | 1.43(0.22, 2.64) | 0.850 | -1.30(-2.54, -0.07) |
| Milks (%) | 11.61(10.89, 12.33) | 7.55(7.01, 8.10) | < 0.001 | -3.77(-4.72, -2.82) | < 0.001 | -2.37(-3.11, -1.63) |
| Vegetables (%) | 12.94(12.21, 13.67) | 10.96(10.51, 11.42) | < 0.001 | -2.06(-3.11, -1.00) | 0.006 | -0.89(-1.56, -0.23) |
| Fruits (%) | 6.04(5.37, 6.71) | 5.33(5.04, 5.61) | < 0.001 | -1.15(-1.89, -0.40) | < 0.001 | -0.89(-1.61, -0.16) |
| Beverages (%) | 14.03(13.39, 14.68) | 18.85(18.25, 19.45) | < 0.001 | 5.55(4.73, 6.36) | < 0.001 | 6.93(6.11, 7.74) |
| Nuts (%) | 5.00(4.62, 5.39) | 7.42(6.91, 7.93) | < 0.001 | 2.13(1.46, 2.79) | < 0.001 | 0.95(0.23, 1.67) |
| Eggs (%) | 1.09(0.98, 1.21) | 1.54(1.39, 1.68) | < 0.001 | 0.46(0.24, 0.68) | < 0.001 | 0.27(0.09, 0.45) |
| Oils (%) | 0.13(0.10, 0.16) | 0.44(0.41, 0.48) | < 0.001 | 0.30(0.25, 0.35) | < 0.001 | 0.32(0.28, 0.36) |
| **Phosphorus** |  |  |  |  |  |  |
| DS (%) | 1.86(1.68, 2.05) | 0.40(0.32, 0.48) | < 0.001 | -1.60(-1.83, -1.36) | < 0.001 | -1.14(-1.37, -0.90) |
| Meats (%) | 25.54(24.34, 26.74) | 27.87(26.84, 28.90) | < 0.001 | 2.52(0.85, 4.20) | 0.003 | 2.25(0.90, 3.59) |
| Grains (%) | 26.22(25.31, 27.14) | 29.26(28.25, 30.26) | < 0.001 | 2.94(1.50, 4.38) | < 0.001 | 1.31(-0.05, 2.67) |
| Milks (%) | 21.55(20.43, 22.67) | 15.46(14.41, 16.52) | < 0.001 | -5.96(-7.66, -4.26) | < 0.001 | -4.70(-5.95, -3.45) |
| Vegetables (%) | 8.31(7.80, 8.83) | 7.17(6.75, 7.59) | < 0.001 | -1.25(-2.08, -0.42) | 0.034 | -0.31(-0.85, 0.24) |
| Fruits (%) | 1.97(1.72, 2.22) | 2.10(1.96, 2.24) | 0.850 | -0.07(-0.37, 0.23) | 0.408 | -0.01(-0.29, 0.28) |
| Beverages (%) | 7.19(6.75, 7.62) | 7.06(6.74, 7.38) | 0.077 | 0.29(-0.28, 0.87) | < 0.001 | 0.47(-0.02, 0.96) |
| Nuts (%) | 3.79(3.34, 4.24) | 5.26(4.74, 5.77) | < 0.001 | 1.25(0.63, 1.88) | 0.009 | 0.77(0.17, 1.36) |
| Eggs (%) | 3.30(2.95, 3.64) | 4.69(4.32, 5.06) | < 0.001 | 1.39(0.77, 2.02) | < 0.001 | 0.86(0.29, 1.43) |
| Oils (%) | 0.27(0.23, 0.31) | 0.75(0.68, 0.81) | < 0.001 | 0.49(0.40, 0.58) | < 0.001 | 0.50(0.42, 0.58) |
| **Potassium** |  |  |  |  |  |  |
| DS (%) | 0.83(0.73, 0.94) | 0.51(0.43, 0.59) | < 0.001 | -0.41(-0.57, -0.24) | < 0.001 | -0.31(-0.46, -0.17) |
| Meats (%) | 20.67(19.69, 21.64) | 22.25(21.23, 23.26) | < 0.001 | 2.08(0.56, 3.59) | < 0.001 | 2.42(1.14, 3.69) |
| Grains (%) | 15.77(14.96, 16.58) | 18.39(17.69, 19.10) | < 0.001 | 2.83(1.77, 3.90) | < 0.001 | 1.02(0.04, 2.00) |
| Milks (%) | 14.08(13.28, 14.88) | 9.96(9.26, 10.67) | < 0.001 | -3.92(-5.06, -2.77) | < 0.001 | -2.88(-3.77, -1.99) |
| Vegetables (%) | 20.55(19.53, 21.58) | 18.62(17.89, 19.34) | < 0.001 | -2.03(-3.57, -0.48) | 0.004 | -0.81(-1.76, 0.15) |
| Fruits (%) | 10.68(9.40, 11.95) | 9.31(8.77, 9.86) | < 0.001 | -2.23(-3.65, -0.82) | < 0.001 | -1.55(-2.86, -0.25) |
| Beverages (%) | 12.11(11.51, 12.72) | 13.32(12.93, 13.71) | < 0.001 | 1.52(0.85, 2.19) | 0.002 | 0.74(0.09, 1.39) |
| Nuts (%) | 3.66(3.34, 3.97) | 4.77(4.34, 5.20) | < 0.001 | 0.94(0.42, 1.46) | 0.020 | 0.43(-0.14, 1.00) |
| Eggs (%) | 1.40(1.25, 1.54) | 2.05(1.85, 2.25) | < 0.001 | 0.65(0.38, 0.93) | < 0.001 | 0.36(0.09, 0.63) |
| Oils (%) | 0.26(0.22, 0.29) | 0.84(0.77, 0.91) | < 0.001 | 0.58(0.49, 0.67) | < 0.001 | 0.61(0.53, 0.69) |
| **Sodium** |  |  |  |  |  |  |
| DS (%) | 0.07(0.05, 0.09) | 0.10(0.07, 0.12) | 0.126 | 0.02(-0.02, 0.06) | 0.119 | -0.01(-0.07, 0.05) |
| Meats (%) | 28.62(27.34, 29.90) | 31.72(30.42, 33.03) | < 0.001 | 3.59(1.58, 5.59) | < 0.001 | 4.07(2.47, 5.68) |
| Grains (%) | 35.83(34.29, 37.38) | 33.97(32.98, 34.97) | < 0.001 | -2.03(-3.99, -0.07) | < 0.001 | -3.84(-5.29, -2.39) |
| Milks (%) | 9.05(8.43, 9.68) | 6.75(6.16, 7.35) | < 0.001 | -2.18(-3.07, -1.29) | < 0.001 | -1.35(-2.06, -0.64) |
| Vegetables (%) | 12.95(12.08, 13.81) | 12.12(11.63, 12.61) | 0.029 | -1.03(-2.17, 0.12) | 0.025 | -0.40(-1.15, 0.36) |
| Fruits (%) | 0.19(0.15, 0.22) | 0.34(0.28, 0.40) | < 0.001 | 0.13(0.05, 0.21) | < 0.001 | 0.16(0.08, 0.23) |
| Beverages (%) | 3.01(2.81, 3.21) | 4.45(4.21, 4.69) | < 0.001 | 1.70(1.38, 2.01) | < 0.001 | 1.95(1.63, 2.26) |
| Nuts (%) | 3.34(2.88, 3.80) | 3.87(3.54, 4.20) | 0.447 | 0.34(-0.14, 0.81) | 0.462 | 0.28(-0.16, 0.72) |
| Eggs (%) | 2.70(2.42, 2.97) | 3.25(3.02, 3.48) | < 0.001 | 0.52(0.06, 0.99) | < 0.001 | 0.09(-0.37, 0.55) |
| Oils (%) | 4.24(3.76, 4.73) | 3.45(3.24, 3.66) | < 0.001 | -1.03(-1.58, -0.49) | < 0.001 | -0.94(-1.32, -0.56) |
| **Zinc** |  |  |  |  |  |  |
| DS (%) | 18.06(16.58, 19.54) | 14.00(12.83, 15.16) | < 0.001 | -5.90(-8.03, -3.76) | < 0.001 | -6.22(-7.98, -4.45) |
| Meats (%) | 31.13(29.48, 32.77) | 29.50(28.29, 30.71) | 0.017 | -0.61(-2.82, 1.60) | 0.048 | -0.77(-2.50, 0.95) |
| Grains (%) | 21.92(20.81, 23.02) | 26.60(25.53, 27.68) | < 0.001 | 5.15(3.60, 6.70) | < 0.001 | 3.12(1.68, 4.55) |
| Milks (%) | 11.35(10.54, 12.16) | 8.89(8.27, 9.51) | < 0.001 | -2.19(-3.32, -1.07) | < 0.001 | -1.05(-1.88, -0.21) |
| Vegetables (%) | 5.76(5.34, 6.19) | 5.23(4.91, 5.55) | 0.012 | -0.62(-1.28, 0.05) | 0.055 | -0.20(-0.69, 0.29) |
| Fruits (%) | 1.32(1.13, 1.51) | 1.50(1.38, 1.61) | 0.009 | 0.06(-0.17, 0.29) | < 0.001 | 0.29(0.09, 0.49) |
| Beverages (%) | 4.46(4.25, 4.67) | 5.92(5.64, 6.20) | < 0.001 | 1.80(1.37, 2.23) | < 0.001 | 3.01(2.58, 3.44) |
| Nuts (%) | 3.63(3.20, 4.07) | 4.49(4.05, 4.93) | 0.006 | 0.81(0.16, 1.46) | 0.145 | 0.33(-0.28, 0.95) |
| Eggs (%) | 2.24(1.99, 2.49) | 3.49(3.18, 3.80) | < 0.001 | 1.25(0.75, 1.75) | < 0.001 | 1.24(0.83, 1.65) |
| Oils (%) | 0.14(0.12, 0.16) | 0.39(0.35, 0.42) | < 0.001 | 0.24(0.20, 0.29) | < 0.001 | 0.25(0.20, 0.29) |
| **Copper** |  |  |  |  |  |  |
| DS (%) | 16.27(14.93, 17.60) | 9.22(8.29, 10.14) | < 0.001 | -8.39(-10.22, -6.56) | < 0.001 | -9.12(-11.04, -7.19) |
| Meats (%) | 14.74(13.94, 15.54) | 14.80(14.00, 15.61) | 0.715 | 0.61(-0.68, 1.89) | 0.298 | 0.13(-1.17, 1.43) |
| Grains (%) | 24.72(23.57, 25.86) | 27.57(26.74, 28.39) | < 0.001 | 3.24(1.97, 4.52) | 0.093 | -1.04(-2.21, 0.13) |
| Milks (%) | 4.27(3.75, 4.79) | 3.31(2.99, 3.63) | < 0.001 | -0.82(-1.46, -0.17) | < 0.001 | -1.08(-1.56, -0.59) |
| Vegetables (%) | 14.51(13.70, 15.32) | 11.22(10.66, 11.78) | < 0.001 | -3.21(-4.47, -1.95) | < 0.001 | -1.46(-2.28, -0.64) |
| Fruits (%) | 6.44(5.67, 7.22) | 6.19(5.75, 6.62) | 0.031 | -0.75(-1.66, 0.15) | 0.838 | -0.09(-0.95, 0.78) |
| Beverages (%) | 13.18(12.36, 14.00) | 17.22(16.72, 17.71) | < 0.001 | 4.93(4.03, 5.83) | < 0.001 | 10.43(9.70, 11.15) |
| Nuts (%) | 5.21(4.60, 5.82) | 8.45(7.64, 9.27) | < 0.001 | 3.02(1.97, 4.08) | < 0.001 | 1.81(0.79, 2.83) |
| Eggs (%) | 0.44(0.39, 0.49) | 1.55(1.35, 1.76) | < 0.001 | 1.12(0.88, 1.36) | 0.100 | 0.02(-0.23, 0.27) |
| Oils (%) | 0.11(0.05, 0.17) | 0.48(0.43, 0.53) | < 0.001 | 0.38(0.29, 0.46) | < 0.001 | 0.40(0.34, 0.46) |
| **Selenium** |  |  |  |  |  |  |
| DS (%) | 7.81(6.96, 8.65) | 6.67(6.06, 7.28) | < 0.001 | -2.03(-3.17, -0.88) | < 0.001 | -2.41(-3.44, -1.38) |
| Meats (%) | 34.57(33.06, 36.07) | 37.21(35.89, 38.54) | < 0.001 | 3.02(0.91, 5.12) | 0.009 | 2.99(1.20, 4.77) |
| Grains (%) | 36.40(34.84, 37.95) | 33.85(32.72, 34.98) | 0.072 | -2.02(-4.19, 0.16) | 0.059 | -2.29(-4.10, -0.47) |
| Milks (%) | 7.62(7.03, 8.21) | 6.34(5.78, 6.90) | < 0.001 | -5.96(-7.66, -4.26) | < 0.001 | -4.70(-5.95, -3.45) |
| Vegetables (%) | 2.83(2.59, 3.06) | 2.32(2.15, 2.48) | < 0.001 | -0.58(-0.92, -0.24) | < 0.001 | -0.25(-0.48, -0.02) |
| Fruits (%) | 0.64(0.55, 0.72) | 0.50(0.44, 0.56) | 0.004 | -0.18(-0.29, -0.07) | 0.560 | 0.04(-0.03, 0.12) |
| Beverages (%) | 2.87(2.68, 3.06) | 1.91(1.72, 2.11) | 0.139 | -0.84(-1.13, -0.55) | < 0.001 | 0.20(-0.03, 0.43) |
| Nuts (%) | 2.06(1.73, 2.40) | 3.57(3.10, 4.05) | < 0.001 | 1.37(0.65, 2.08) | < 0.001 | 1.30(0.62, 1.98) |
| Eggs (%) | 5.03(4.49, 5.56) | 7.20(6.70, 7.70) | < 0.001 | 2.14(1.18, 3.09) | < 0.001 | 1.55(0.76, 2.34) |
| Oils (%) | 0.19(0.16, 0.22) | 0.43(0.38, 0.48) | < 0.001 | 0.24(0.18, 0.31) | < 0.001 | 0.26(0.21, 0.32) |
| **FB** |  |  |  |  |  |  |
| **Calcium** |  |  |  |  |  |  |
| Meats (%) | 9.89(9.00, 10.78) | 13.11(12.14, 14.08) | < 0.001 | 3.76(2.37, 5.14) | < 0.001 | 3.92(2.73, 5.11) |
| Grains (%) | 28.05(26.94, 29.16) | 26.73(25.60, 27.86) | 0.029 | -0.93(-2.68, 0.83) | 0.025 | -3.83(-5.37, -2.28) |
| Milks (%) | 37.60(36.03, 39.16) | 27.15(25.70, 28.60) | < 0.001 | -10.79(-13.23, -8.35) | < 0.001 | -8.45(-10.22, -6.68) |
| Vegetables (%) | 8.31(7.58, 9.05) | 6.80(6.29, 7.32) | < 0.001 | -1.91(-3.05, -0.77) | 0.318 | -0.39(-1.12, 0.35) |
| Fruits (%) | 2.59(2.26, 2.92) | 3.34(3.09, 3.60) | 0.809 | 0.52(0.07, 0.96) | < 0.001 | -1.44(-2.00, -0.87) |
| Beverages (%) | 8.08(7.66, 8.51) | 16.32(15.70, 16.95) | < 0.001 | 8.42(7.62, 9.22) | < 0.001 | 9.58(8.85, 10.31) |
| Nuts (%) | 2.32(2.07, 2.57) | 2.87(2.55, 3.18) | 0.065 | 0.40(0.07, 0.73) | 0.091 | 0.41(0.07, 0.75) |
| Eggs (%) | 2.66(2.31, 3.00) | 3.24(2.97, 3.51) | 0.008 | 0.60(0.07, 1.13) | 0.006 | 0.21(-0.22, 0.63) |
| Oils (%) | 0.50(0.41, 0.58) | 0.46(0.43, 0.48) | 0.001 | -0.05(-0.16, 0.05) | 0.518 | 0.00(-0.07, 0.07) |
| **Iron** |  |  |  |  |  |  |
| Meats (%) | 22.65(21.29, 24.01) | 21.71(20.62, 22.80) | 0.028 | -0.35(-2.15, 1.45) | 0.959 | 1.06(-0.32, 2.44) |
| Grains (%) | 46.71(45.12, 48.31) | 48.35(47.30, 49.41) | 0.018 | 1.74(-0.47, 3.96) | 0.071 | -1.99(-3.69, -0.29) |
| Milks (%) | 3.25(2.90, 3.61) | 2.46(2.25, 2.67) | < 0.001 | -0.85(-1.33, -0.36) | 0.016 | -0.39(-0.75, -0.02) |
| Vegetables (%) | 10.90(10.20, 11.59) | 9.12(8.72, 9.52) | < 0.001 | -1.99(-2.90, -1.07) | 0.943 | 0.27(-0.25, 0.78) |
| Fruits (%) | 3.71(3.23, 4.20) | 2.43(2.22, 2.63) | < 0.001 | -1.62(-2.16, -1.07) | < 0.001 | -1.07(-1.53, -0.61) |
| Beverages (%) | 5.95(5.70, 6.20) | 5.56(5.25, 5.86) | 0.050 | -0.29(-0.71, 0.14) | 0.947 | -0.45(-0.97, 0.07) |
| Nuts (%) | 4.15(3.76, 4.54) | 5.45(4.93, 5.97) | < 0.001 | 1.15(0.52, 1.78) | 0.001 | 0.96(0.37, 1.56) |
| Eggs (%) | 2.38(2.15, 2.62) | 4.40(4.01, 4.79) | < 0.001 | 1.97(1.41, 2.53) | < 0.001 | 1.41(0.88, 1.95) |
| Oils (%) | 0.30(0.26, 0.34) | 0.54(0.49, 0.59) | < 0.001 | 0.22(0.16, 0.28) | < 0.001 | 0.21(0.16, 0.27) |
| **Magnesium** |  |  |  |  |  |  |
| Meats (%) | 16.95(16.11, 17.79) | 16.53(15.79, 17.27) | 0.079 | -0.08(-1.33, 1.17) | 0.496 | -0.18(-1.19, 0.83) |
| Grains (%) | 27.42(26.38, 28.46) | 27.86(27.12, 28.59) | 0.568 | 0.34(-0.99, 1.67) | 0.013 | -2.56(-3.92, -1.19) |
| Milks (%) | 12.71(11.97, 13.45) | 8.08(7.48, 8.69) | < 0.001 | -4.41(-5.40, -3.41) | < 0.001 | -2.91(-3.69, -2.13) |
| Vegetables (%) | 14.13(13.36, 14.90) | 11.74(11.23, 12.26) | < 0.001 | -2.56(-3.70, -1.43) | < 0.001 | -1.39(-2.09, -0.68) |
| Fruits (%) | 6.68(5.91, 7.45) | 5.70(5.37, 6.03) | < 0.001 | -1.53(-2.39, -0.67) | < 0.001 | -1.17(-1.96, -0.38) |
| Beverages (%) | 15.32(14.63, 16.01) | 20.04(19.47, 20.62) | < 0.001 | 5.37(4.57, 6.17) | < 0.001 | 6.85(6.05, 7.66) |
| Nuts (%) | 5.47(5.03, 5.90) | 7.95(7.39, 8.51) | < 0.001 | 2.11(1.38, 2.84) | 0.002 | 0.81(0.04, 1.58) |
| Eggs (%) | 1.18(1.05, 1.31) | 1.64(1.49, 1.79) | < 0.001 | 0.47(0.24, 0.70) | < 0.001 | 0.22(0.01, 0.43) |
| Oils (%) | 0.15(0.12, 0.18) | 0.47(0.43, 0.51) | < 0.001 | 0.31(0.26, 0.36) | < 0.001 | 0.34(0.30, 0.38) |
| **Phosphorus** |  |  |  |  |  |  |
| Meats (%) | 26.00(24.80, 27.20) | 27.97(26.94, 28.99) | 0.001 | 2.12(0.43, 3.81) | 0.018 | 1.95(0.61, 3.29) |
| Grains (%) | 26.75(25.80, 27.70) | 29.37(28.36, 30.39) | < 0.001 | 2.48(1.00, 3.97) | < 0.001 | 1.00(-0.41, 2.41) |
| Milks (%) | 21.94(20.80, 23.09) | 15.53(14.47, 16.59) | < 0.001 | -6.31(-8.03, -4.59) | < 0.001 | -4.97(-6.20, -3.73) |
| Vegetables (%) | 8.48(7.95, 9.00) | 7.21(6.78, 7.63) | < 0.001 | -1.38(-2.22, -0.55) | 0.008 | -0.38(-0.94, 0.17) |
| Fruits (%) | 2.02(1.76, 2.27) | 2.11(1.96, 2.25) | 0.885 | -0.11(-0.42, 0.19) | 0.319 | -0.03(-0.32, 0.26) |
| Beverages (%) | 7.34(6.88, 7.79) | 7.09(6.76, 7.41) | 0.243 | 0.16(-0.43, 0.75) | < 0.001 | 0.40(-0.09, 0.89) |
| Nuts (%) | 3.85(3.39, 4.31) | 5.28(4.76, 5.80) | < 0.001 | 1.21(0.58, 1.84) | 0.018 | 0.73(0.13, 1.33) |
| Eggs (%) | 3.35(2.99, 3.70) | 4.71(4.34, 5.09) | < 0.001 | 1.36(0.73, 2.00) | < 0.001 | 0.82(0.25, 1.40) |
| Oils (%) | 0.27(0.24, 0.31) | 0.75(0.69, 0.81) | < 0.001 | 0.49(0.40, 0.57) | < 0.001 | 0.50(0.42, 0.58) |
| **Potassium** |  |  |  |  |  |  |
| Meats (%) | 20.83(19.84, 21.81) | 22.34(21.33, 23.36) | < 0.001 | 2.00(0.47, 3.53) | < 0.001 | 2.36(1.08, 3.63) |
| Grains (%) | 15.92(15.10, 16.75) | 18.48(17.77, 19.19) | < 0.001 | 2.75(1.66, 3.84) | < 0.001 | 0.97(-0.03, 1.96) |
| Milks (%) | 14.20(13.40, 15.00) | 10.02(9.31, 10.73) | < 0.001 | -3.99(-5.13, -2.84) | < 0.001 | -2.94(-3.83, -2.05) |
| Vegetables (%) | 20.72(19.69, 21.75) | 18.72(17.99, 19.45) | < 0.001 | -2.10(-3.66, -0.54) | 0.002 | -0.86(-1.82, 0.10) |
| Fruits (%) | 10.76(9.47, 12.06) | 9.36(8.82, 9.91) | < 0.001 | -2.28(-3.71, -0.85) | < 0.001 | -1.59(-2.91, -0.28) |
| Beverages (%) | 12.22(11.61, 12.83) | 13.39(13.01, 13.78) | < 0.001 | 1.47(0.80, 2.14) | 0.004 | 0.70(0.05, 1.35) |
| Nuts (%) | 3.68(3.36, 4.01) | 4.80(4.36, 5.23) | < 0.001 | 0.93(0.41, 1.46) | 0.025 | 0.42(-0.15, 1.00) |
| Eggs (%) | 1.41(1.25, 1.56) | 2.06(1.86, 2.26) | < 0.001 | 0.65(0.37, 0.93) | < 0.001 | 0.35(0.09, 0.62) |
| Oils (%) | 0.26(0.22, 0.30) | 0.84(0.77, 0.91) | < 0.001 | 0.58(0.49, 0.67) | < 0.001 | 0.61(0.53, 0.69) |
| **Sodium** |  |  |  |  |  |  |
| Meats (%) | 28.64(27.36, 29.92) | 31.75(30.45, 33.06) | < 0.001 | 3.60(1.59, 5.61) | < 0.001 | 4.08(2.48, 5.68) |
| Grains (%) | 35.86(34.32, 37.41) | 34.00(33.00, 34.99) | < 0.001 | -2.04(-4.00, -0.08) | < 0.001 | -3.85(-5.31, -2.39) |
| Milks (%) | 9.06(8.43, 9.69) | 6.76(6.16, 7.36) | < 0.001 | -2.18(-3.07, -1.29) | < 0.001 | -1.35(-2.06, -0.64) |
| Vegetables (%) | 12.95(12.08, 13.83) | 12.14(11.64, 12.63) | 0.029 | -1.02(-2.17, 0.13) | 0.023 | -0.40(-1.15, 0.36) |
| Fruits (%) | 0.19(0.15, 0.22) | 0.34(0.28, 0.40) | < 0.001 | 0.13(0.05, 0.21) | < 0.001 | 0.16(0.08, 0.23) |
| Beverages (%) | 3.01(2.81, 3.21) | 4.45(4.21, 4.70) | < 0.001 | 1.70(1.39, 2.02) | < 0.001 | 1.95(1.63, 2.27) |
| Nuts (%) | 3.34(2.88, 3.80) | 3.87(3.54, 4.20) | 0.495 | 0.34(-0.14, 0.81) | 0.527 | 0.28(-0.16, 0.72) |
| Eggs (%) | 2.70(2.42, 2.97) | 3.25(3.02, 3.49) | < 0.001 | 0.53(0.06, 0.99) | < 0.001 | 0.09(-0.38, 0.55) |
| Oils (%) | 4.25(3.76, 4.73) | 3.45(3.24, 3.66) | < 0.001 | -1.03(-1.58, -0.49) | < 0.001 | -0.94(-1.32, -0.57) |
| **Zinc** |  |  |  |  |  |  |
| Meats (%) | 37.30(35.39, 39.21) | 33.95(32.75, 35.14) | < 0.001 | -2.96(-5.28, -0.64) | < 0.001 | -3.02(-4.75, -1.28) |
| Grains (%) | 26.96(25.65, 28.27) | 30.63(29.63, 31.62) | < 0.001 | 3.62(1.83, 5.40) | 0.001 | 0.89(-0.73, 2.52) |
| Milks (%) | 14.09(13.21, 14.97) | 10.64(9.84, 11.45) | < 0.001 | -3.42(-4.69, -2.15) | < 0.001 | -2.26(-3.23, -1.28) |
| Vegetables (%) | 7.17(6.71, 7.63) | 6.20(5.80, 6.60) | < 0.001 | -1.17(-1.90, -0.44) | < 0.001 | -0.67(-1.18, -0.15) |
| Fruits (%) | 1.69(1.44, 1.95) | 1.78(1.64, 1.91) | 0.656 | -0.11(-0.42, 0.21) | 0.030 | 0.21(-0.02, 0.45) |
| Beverages (%) | 5.49(5.25, 5.73) | 6.92(6.63, 7.21) | < 0.001 | 1.66(1.22, 2.10) | < 0.001 | 3.27(2.83, 3.71) |
| Nuts (%) | 4.44(3.96, 4.92) | 5.35(4.87, 5.83) | 0.087 | 0.76(0.06, 1.46) | 0.934 | 0.08(-0.59, 0.75) |
| Eggs (%) | 2.68(2.39, 2.97) | 4.11(3.79, 4.42) | < 0.001 | 1.37(0.83, 1.90) | < 0.001 | 1.23(0.77, 1.69) |
| Oils (%) | 0.18(0.15, 0.21) | 0.45(0.41, 0.49) | < 0.001 | 0.26(0.20, 0.31) | < 0.001 | 0.26(0.22, 0.31) |
| **Copper** |  |  |  |  |  |  |
| Meats (%) | 17.41(16.48, 18.34) | 16.21(15.36, 17.06) | < 0.001 | -0.90(-2.30, 0.49) | < 0.001 | -1.50(-2.78, -0.21) |
| Grains (%) | 29.62(28.36, 30.87) | 30.27(29.51, 31.04) | 0.014 | 0.64(-0.89, 2.17) | < 0.001 | -4.70(-5.96, -3.43) |
| Milks (%) | 5.18(4.60, 5.76) | 3.71(3.35, 4.07) | < 0.001 | -1.37(-2.09, -0.65) | < 0.001 | -1.84(-2.35, -1.33) |
| Vegetables (%) | 17.34(16.48, 18.20) | 12.38(11.76, 13.00) | < 0.001 | -5.04(-6.34, -3.74) | < 0.001 | -3.18(-4.01, -2.36) |
| Fruits (%) | 7.89(6.95, 8.83) | 6.83(6.38, 7.28) | < 0.001 | -1.78(-2.87, -0.69) | 0.003 | -1.00(-1.98, -0.02) |
| Beverages (%) | 15.57(14.66, 16.47) | 19.09(18.57, 19.61) | < 0.001 | 4.27(3.36, 5.18) | < 0.001 | 10.82(10.10, 11.53) |
| Nuts (%) | 6.18(5.52, 6.85) | 9.27(8.42, 10.12) | < 0.001 | 2.77(1.64, 3.91) | 0.003 | 1.16(0.08, 2.25) |
| Eggs (%) | 0.52(0.46, 0.57) | 1.71(1.50, 1.93) | < 0.001 | 1.20(0.95, 1.45) | 0.878 | -0.18(-0.47, 0.11) |
| Oils (%) | 0.15(0.06, 0.24) | 0.54(0.49, 0.59) | < 0.001 | 0.39(0.27, 0.50) | < 0.001 | 0.43(0.37, 0.49) |
| **Selenium** |  |  |  |  |  |  |
| Meats (%) | 37.27(35.61, 38.94) | 39.75(38.31, 41.18) | 0.002 | 2.51(0.19, 4.84) | 0.235 | 2.26(0.31, 4.21) |
| Grains (%) | 39.58(37.98, 41.17) | 36.09(34.93, 37.25) | 0.001 | -3.30(-5.65, -0.94) | 0.001 | -3.51(-5.39, -1.63) |
| Milks (%) | 8.43(7.83, 9.03) | 6.91(6.30, 7.53) | < 0.001 | -1.45(-2.32, -0.57) | < 0.001 | -1.71(-2.40, -1.01) |
| Vegetables (%) | 3.06(2.81, 3.31) | 2.54(2.33, 2.75) | < 0.001 | -0.61(-0.99, -0.23) | < 0.001 | -0.30(-0.57, -0.03) |
| Fruits (%) | 0.70(0.60, 0.80) | 0.55(0.48, 0.62) | 0.002 | -0.20(-0.33, -0.07) | 0.943 | 0.04(-0.04, 0.13) |
| Beverages (%) | 3.13(2.94, 3.31) | 2.12(1.89, 2.34) | 0.173 | -0.91(-1.22, -0.61) | < 0.001 | 0.24(-0.01, 0.49) |
| Nuts (%) | 2.24(1.87, 2.60) | 3.89(3.39, 4.38) | < 0.001 | 1.48(0.75, 2.21) | < 0.001 | 1.29(0.60, 1.99) |
| Eggs (%) | 5.39(4.82, 5.96) | 7.71(7.18, 8.24) | < 0.001 | 2.23(1.23, 3.24) | < 0.001 | 1.41(0.54, 2.29) |
| Oils (%) | 0.21(0.18, 0.24) | 0.46(0.41, 0.51) | < 0.001 | 0.25(0.19, 0.32) | < 0.001 | 0.28(0.22, 0.34) |

Abbreviations: NHANES, National Health and Nutrition Examination Survey; DSs, Dietary supplements; FB, foods and beverages; Milks, Milk and milk products; Meats, Meat, poultry, fish and mixtures; Eggs, Eggs; Nuts, Legumes, nuts and seeds; Grains, Grain products; Fruits, Fruits; Vegetables, Vegetables; Oils, Fats, oils and salad dressings; Beverages, Sugar, sweeteners and, beverages.

a Data were adjusted for NHANES survey weights.

b Results were adjusted for gender, age, race/ethnicity, education level, and family income.

c 1999-2000 vs. 2017-2020, and differences were adjusted for gender, age, race/ethnicity, education level, and family income.

d 2003-2004 vs. 2017-2020, and differences were adjusted for gender, age, race/ethnicity, education level, and family income.

**Supplementary Table 3.** Trends in Estimated Absolute Intake of Nine Minerals from Nine Food Groups and Dietary Supplements by NHANES Survey Cycle, 1999-2020.

|  | Survey-Weighted Mean (95% CI) ^a^ | | | | | | | | | | 1999-2020 | | 2003-2020 | |
| --- | --- | --- | --- | --- | --- | --- | --- | --- | --- | --- | --- | --- | --- | --- |
|  | 1999-2000(n=3745) | 2001-2002(n=4171) | 2003-2004(n=4085) | 2005-2006(n=4059) | 2007-2008(n=5155) | 2009-2010(n=5505) | 2011-2012(n=4584) | 2013-2014(n=4789) | 2015-2016(n=4798) | 2017-2020(n=7332) | FDR for Trend ^b^ | Difference (95% CI) ^c^ | FDR for Trend ^b^ | Difference (95% CI) ^d^ |
| **Calcium** |  |  |  |  |  |  |  |  |  |  |  |  |  |  |
| DS, mg | 159.13(136.72, 181.54) | 158.79(141.99, 175.59) | 206.92(185.70, 228.13) | 196.87(183.66, 210.07) | 188.23(164.65, 211.82) | 174.05(161.28, 186.82) | 152.65(134.88, 170.41) | 155.04(138.74, 171.33) | 136.77(118.96, 154.58) | 125.82(114.19, 137.44) | < 0.001 | -48.26(-73.46, -23.06) | < 0.001 | -93.76(-114.84, -72.68) |
| Meats, mg | 55.97(51.08, 60.86) | 61.04(56.58, 65.51) | 64.65(60.94, 68.36) | 65.95(63.15, 68.74) | 66.68(63.49, 69.86) | 75.80(71.11, 80.48) | 72.71(68.27, 77.14) | 70.96(67.09, 74.83) | 80.30(75.45, 85.14) | 106.55(99.41, 113.68) | < 0.001 | 55.15(45.59, 64.70) | < 0.001 | 46.86(38.19, 55.54) |
| Grains, mg | 203.96(193.56, 214.36) | 228.75(217.70, 239.81) | 240.86(230.50, 251.22) | 237.96(230.41, 245.51) | 244.29(238.19, 250.39) | 253.80(245.47, 262.12) | 278.26(266.28, 290.24) | 269.98(260.25, 279.70) | 260.71(249.95, 271.48) | 234.21(223.92, 244.49) | < 0.001 | 33.96(17.85, 50.06) | 0.015 | -3.49(-19.77, 12.78) |
| Milks, mg | 366.72(343.42, 390.02) | 361.91(350.10, 373.73) | 338.22(314.00, 362.45) | 352.32(333.65, 370.99) | 331.95(308.59, 355.32) | 370.95(350.67, 391.23) | 329.16(310.21, 348.10) | 324.19(311.90, 336.49) | 316.35(295.14, 337.55) | 277.18(257.77, 296.59) | < 0.001 | -90.30(-122.47, -58.13) | < 0.001 | -56.43(-84.97, -27.90) |
| Vegetables, mg | 50.38(46.44, 54.32) | 49.58(44.86, 54.31) | 50.36(47.59, 53.12) | 51.26(48.22, 54.29) | 48.80(45.89, 51.72) | 53.48(50.58, 56.38) | 54.96(49.52, 60.41) | 57.11(53.62, 60.60) | 52.16(48.52, 55.80) | 55.88(50.85, 60.91) | 0.039 | 4.11(-3.19, 11.40) | 0.042 | 4.76(-1.70, 11.22) |
| Fruits, mg | 15.95(13.13, 18.78) | 18.58(15.18, 21.98) | 38.83(33.35, 44.30) | 37.73(33.57, 41.89) | 51.76(41.32, 62.19) | 51.83(47.04, 56.61) | 36.75(31.01, 42.49) | 36.48(34.13, 38.83) | 33.50(31.14, 35.86) | 30.45(27.34, 33.55) | 0.001 | 12.61(7.91, 17.31) | < 0.001 | -8.93(-15.06, -2.79) |
| Beverages, mg | 46.79(43.90, 49.68) | 45.64(42.16, 49.12) | 45.22(42.83, 47.61) | 107.89(103.41, 112.37) | 104.21(97.28, 111.14) | 108.66(102.08, 115.24) | 117.23(110.28, 124.17) | 127.93(119.78, 136.08) | 134.67(123.30, 146.04) | 135.37(127.36, 143.39) | < 0.001 | 90.88(80.63, 101.12) | < 0.001 | 90.99(81.13, 100.86) |
| Nuts, mg | 17.65(14.89, 20.42) | 15.33(13.08, 17.57) | 16.45(14.23, 18.67) | 17.44(14.86, 20.02) | 24.21(20.49, 27.93) | 27.65(25.02, 30.28) | 17.82(16.06, 19.59) | 18.03(16.36, 19.69) | 22.91(19.37, 26.44) | 23.03(20.77, 25.28) | < 0.001 | 4.86(1.25, 8.47) | 0.134 | 5.06(1.99, 8.13) |
| Eggs, mg | 16.38(14.42, 18.33) | 15.63(14.34, 16.92) | 19.61(17.45, 21.77) | 19.03(17.14, 20.91) | 19.45(17.96, 20.95) | 22.32(20.06, 24.59) | 20.56(18.75, 22.37) | 22.50(20.09, 24.91) | 22.47(20.74, 24.20) | 26.73(24.74, 28.72) | < 0.001 | 10.75(7.55, 13.94) | < 0.001 | 6.33(3.00, 9.65) |
| Oils, mg | 3.08(2.46, 3.70) | 2.88(2.40, 3.35) | 3.25(2.81, 3.69) | 2.73(2.39, 3.08) | 2.24(1.98, 2.51) | 2.36(2.16, 2.55) | 2.64(2.15, 3.12) | 2.53(2.26, 2.80) | 2.18(1.91, 2.45) | 3.67(3.41, 3.92) | 0.959 | 0.52(-0.31, 1.35) | 0.188 | 0.30(-0.35, 0.96) |
| **Iron** |  |  |  |  |  |  |  |  |  |  |  |  |  |  |
| DS, mg | 4.68(4.20, 5.15) | 4.39(4.04, 4.74) | 3.99(3.31, 4.66) | 3.66(3.23, 4.08) | 3.45(3.21, 3.69) | 3.55(3.23, 3.86) | 2.65(2.26, 3.04) | 3.61(2.89, 4.32) | 2.82(2.36, 3.28) | 3.07(2.70, 3.45) | < 0.001 | -1.94(-2.63, -1.25) | 0.001 | -1.15(-1.97, -0.33) |
| Meats, mg | 3.03(2.85, 3.20) | 2.93(2.77, 3.10) | 2.93(2.82, 3.03) | 3.03(2.92, 3.14) | 2.97(2.87, 3.06) | 2.75(2.63, 2.88) | 2.56(2.46, 2.66) | 2.46(2.38, 2.55) | 2.48(2.35, 2.60) | 2.69(2.56, 2.81) | < 0.001 | -0.29(-0.52, -0.05) | < 0.001 | -0.20(-0.37, -0.02) |
| Grains, mg | 7.40(6.96, 7.85) | 8.03(7.57, 8.50) | 8.12(7.70, 8.54) | 8.52(8.32, 8.72) | 8.33(8.01, 8.66) | 8.34(8.10, 8.57) | 8.52(8.30, 8.75) | 8.11(7.87, 8.35) | 7.56(7.32, 7.80) | 6.87(6.67, 7.07) | < 0.001 | -0.61(-1.16, -0.06) | < 0.001 | -1.26(-1.71, -0.80) |
| Milks, mg | 0.51(0.39, 0.63) | 0.46(0.36, 0.55) | 0.42(0.36, 0.47) | 0.44(0.38, 0.50) | 0.35(0.32, 0.38) | 0.42(0.37, 0.46) | 0.29(0.27, 0.31) | 0.29(0.26, 0.31) | 0.32(0.30, 0.34) | 0.30(0.28, 0.32) | < 0.001 | -0.25(-0.42, -0.08) | < 0.001 | -0.13(-0.19, -0.07) |
| Vegetables, mg | 1.39(1.31, 1.46) | 1.16(1.06, 1.25) | 1.20(1.16, 1.24) | 1.18(1.11, 1.24) | 1.13(1.09, 1.18) | 1.15(1.10, 1.20) | 1.13(1.02, 1.24) | 1.08(1.02, 1.15) | 1.01(0.96, 1.06) | 1.12(1.05, 1.18) | < 0.001 | -0.30(-0.41, -0.19) | < 0.001 | -0.10(-0.18, -0.02) |
| Fruits, mg | 0.49(0.41, 0.56) | 0.50(0.46, 0.54) | 0.47(0.41, 0.53) | 0.49(0.45, 0.52) | 0.34(0.30, 0.37) | 0.36(0.35, 0.38) | 0.34(0.31, 0.38) | 0.33(0.31, 0.36) | 0.33(0.30, 0.36) | 0.29(0.26, 0.32) | < 0.001 | -0.24(-0.32, -0.16) | < 0.001 | -0.20(-0.26, -0.13) |
| Beverages, mg | 0.71(0.67, 0.74) | 0.56(0.53, 0.59) | 0.80(0.76, 0.84) | 0.76(0.74, 0.79) | 0.73(0.69, 0.77) | 0.71(0.68, 0.75) | 0.87(0.82, 0.93) | 0.76(0.70, 0.82) | 0.78(0.66, 0.89) | 0.71(0.65, 0.76) | 0.004 | 0.01(-0.07, 0.08) | 0.720 | -0.08(-0.16, 0.00) |
| Nuts, mg | 0.66(0.57, 0.75) | 0.61(0.53, 0.68) | 0.64(0.56, 0.72) | 0.72(0.61, 0.82) | 0.71(0.63, 0.79) | 0.80(0.75, 0.85) | 0.66(0.59, 0.73) | 0.62(0.58, 0.66) | 0.74(0.64, 0.83) | 0.75(0.66, 0.83) | 0.442 | 0.06(-0.05, 0.17) | 0.623 | 0.05(-0.05, 0.16) |
| Eggs, mg | 0.29(0.26, 0.31) | 0.32(0.30, 0.35) | 0.37(0.33, 0.41) | 0.35(0.32, 0.38) | 0.37(0.34, 0.39) | 0.35(0.32, 0.39) | 0.38(0.36, 0.41) | 0.41(0.38, 0.45) | 0.44(0.41, 0.46) | 0.53(0.48, 0.57) | < 0.001 | 0.24(0.18, 0.30) | < 0.001 | 0.14(0.07, 0.20) |
| Oils, mg | 0.04(0.03, 0.04) | 0.05(0.04, 0.05) | 0.04(0.04, 0.05) | 0.04(0.03, 0.04) | 0.04(0.03, 0.05) | 0.05(0.04, 0.05) | 0.03(0.03, 0.04) | 0.03(0.03, 0.04) | 0.03(0.03, 0.04) | 0.06(0.06, 0.07) | 0.007 | 0.03(0.02, 0.03) | 0.001 | 0.02(0.01, 0.03) |
| **Magnesium** |  |  |  |  |  |  |  |  |  |  |  |  |  |  |
| DS, mg | 37.13(32.56, 41.70) | 36.59(33.00, 40.18) | 38.62(33.97, 43.28) | 38.54(34.38, 42.71) | 38.18(29.99, 46.36) | 28.71(25.83, 31.60) | 27.59(22.50, 32.69) | 28.99(25.40, 32.58) | 33.15(25.89, 40.41) | 31.98(26.78, 37.17) | < 0.001 | -9.61(-16.60, -2.61) | 0.001 | -8.49(-15.03, -1.95) |
| Meats, mg | 41.68(40.01, 43.36) | 39.99(38.15, 41.84) | 41.17(39.43, 42.90) | 43.06(41.81, 44.30) | 43.29(41.72, 44.87) | 43.90(42.13, 45.67) | 41.08(39.89, 42.27) | 42.92(41.14, 44.70) | 42.49(40.89, 44.08) | 43.75(41.93, 45.58) | 0.070 | 1.80(-1.26, 4.87) | 0.616 | 2.38(-0.23, 4.99) |
| Grains, mg | 74.89(70.26, 79.53) | 78.58(75.12, 82.04) | 79.21(74.13, 84.28) | 82.97(80.58, 85.36) | 83.06(79.40, 86.72) | 84.31(81.14, 87.49) | 90.28(87.68, 92.88) | 86.15(84.44, 87.86) | 86.24(83.12, 89.35) | 79.46(77.08, 81.84) | 0.004 | 2.06(-3.01, 7.13) | 0.917 | -1.98(-6.83, 2.87) |
| Milks, mg | 37.15(34.20, 40.10) | 34.03(32.23, 35.84) | 30.43(28.09, 32.77) | 31.68(29.93, 33.43) | 29.13(27.13, 31.13) | 31.76(30.29, 33.22) | 26.12(24.41, 27.83) | 25.28(24.07, 26.48) | 25.40(23.66, 27.14) | 23.14(21.50, 24.77) | < 0.001 | -14.56(-18.15, -10.97) | < 0.001 | -7.01(-9.53, -4.49) |
| Vegetables, mg | 37.00(35.47, 38.52) | 34.60(32.25, 36.95) | 33.90(32.83, 34.98) | 33.95(31.94, 35.96) | 33.20(32.01, 34.38) | 33.31(31.89, 34.73) | 33.90(31.58, 36.23) | 32.59(31.11, 34.08) | 31.84(30.43, 33.25) | 34.73(32.46, 37.00) | 0.003 | -3.18(-6.32, -0.03) | 0.424 | 0.53(-2.24, 3.29) |
| Fruits, mg | 18.56(15.92, 21.20) | 18.85(17.54, 20.17) | 17.44(15.36, 19.52) | 18.11(16.70, 19.52) | 18.61(16.70, 20.53) | 19.84(18.87, 20.82) | 19.10(17.52, 20.68) | 18.16(16.84, 19.47) | 17.86(16.26, 19.47) | 17.01(15.80, 18.22) | 0.001 | -3.35(-6.20, -0.51) | 0.008 | -1.60(-3.93, 0.73) |
| Beverages, mg | 39.78(38.15, 41.41) | 33.68(31.08, 36.28) | 34.66(33.31, 36.02) | 51.28(48.97, 53.60) | 49.22(46.99, 51.44) | 52.55(50.25, 54.85) | 55.93(53.01, 58.85) | 57.23(54.44, 60.03) | 60.95(56.42, 65.47) | 57.97(55.48, 60.46) | < 0.001 | 19.07(15.76, 22.37) | < 0.001 | 23.52(20.41, 26.62) |
| Nuts, mg | 18.94(16.78, 21.10) | 19.14(16.38, 21.90) | 20.37(18.40, 22.35) | 22.38(20.24, 24.51) | 23.85(20.94, 26.76) | 25.94(24.33, 27.54) | 25.40(22.90, 27.90) | 26.86(24.47, 29.25) | 28.74(24.64, 32.84) | 28.24(25.73, 30.75) | < 0.001 | 7.90(4.43, 11.37) | < 0.001 | 5.83(2.57, 9.09) |
| Eggs, mg | 2.59(2.36, 2.83) | 2.79(2.58, 2.99) | 3.22(2.87, 3.57) | 3.16(2.90, 3.42) | 3.26(3.05, 3.47) | 3.35(3.01, 3.70) | 3.43(3.19, 3.67) | 3.71(3.36, 4.05) | 3.85(3.58, 4.12) | 4.28(3.96, 4.61) | < 0.001 | 1.68(1.22, 2.15) | < 0.001 | 0.90(0.40, 1.41) |
| Oils, mg | 0.35(0.27, 0.44) | 0.35(0.29, 0.42) | 0.32(0.28, 0.35) | 0.32(0.27, 0.37) | 0.36(0.31, 0.41) | 0.35(0.31, 0.39) | 0.47(0.35, 0.58) | 0.44(0.36, 0.52) | 0.38(0.33, 0.44) | 1.32(1.20, 1.43) | < 0.001 | 0.93(0.78, 1.08) | < 0.001 | 0.97(0.84, 1.10) |
| **Phosphorus** |  |  |  |  |  |  |  |  |  |  |  |  |  |  |
| DS, mg | 23.21(20.12, 26.30) | 19.75(17.66, 21.85) | 19.37(16.66, 22.09) | 17.51(14.66, 20.36) | 16.24(13.66, 18.81) | 8.93(7.58, 10.29) | 6.90(4.79, 9.01) | 6.75(4.97, 8.54) | 5.69(4.79, 6.58) | 5.21(4.00, 6.43) | < 0.001 | -19.84(-23.54, -16.15) | < 0.001 | -14.87(-17.60, -12.15) |
| Meats, mg | 303.92(290.54, 317.31) | 298.35(286.82, 309.89) | 312.87(299.59, 326.15) | 330.82(322.44, 339.21) | 328.93(318.06, 339.80) | 356.94(343.82, 370.07) | 333.69(321.91, 345.47) | 347.19(334.85, 359.54) | 347.27(332.77, 361.78) | 358.57(344.86, 372.27) | < 0.001 | 55.01(32.12, 77.89) | < 0.001 | 46.42(26.90, 65.93) |
| Grains, mg | 321.09(305.97, 336.20) | 336.79(322.53, 351.04) | 347.25(332.99, 361.51) | 357.16(346.40, 367.91) | 370.05(358.77, 381.33) | 376.71(362.82, 390.61) | 420.40(405.15, 435.64) | 406.98(395.96, 418.00) | 410.09(395.40, 424.79) | 376.14(362.24, 390.03) | < 0.001 | 50.22(28.99, 71.46) | < 0.001 | 25.38(5.65, 45.11) |
| Milks, mg | 300.79(280.90, 320.68) | 300.49(290.94, 310.05) | 278.91(259.46, 298.36) | 286.49(270.63, 302.34) | 269.71(250.76, 288.65) | 291.21(276.43, 305.99) | 247.91(232.64, 263.18) | 241.06(232.20, 249.93) | 239.02(221.74, 256.31) | 211.46(196.41, 226.51) | < 0.001 | -90.02(-116.59, -63.45) | < 0.001 | -63.76(-85.57, -41.94) |
| Vegetables, mg | 93.90(89.47, 98.33) | 89.42(84.70, 94.15) | 89.95(85.49, 94.40) | 88.65(83.62, 93.68) | 87.65(84.71, 90.59) | 90.35(86.76, 93.94) | 86.59(81.90, 91.27) | 88.69(84.50, 92.88) | 84.82(80.22, 89.43) | 90.78(84.55, 97.00) | 0.157 | -4.25(-13.22, 4.72) | 0.927 | 1.78(-6.22, 9.78) |
| Fruits, mg | 21.77(18.61, 24.92) | 25.34(23.64, 27.04) | 22.93(20.13, 25.74) | 24.06(22.20, 25.91) | 30.63(26.79, 34.46) | 32.64(30.84, 34.43) | 30.71(27.66, 33.75) | 30.28(28.02, 32.54) | 29.53(26.55, 32.51) | 26.05(23.87, 28.23) | 0.009 | 1.82(-2.14, 5.79) | 0.409 | 1.56(-2.00, 5.12) |
| Beverages, mg | 80.04(74.82, 85.27) | 86.87(79.15, 94.58) | 81.26(77.47, 85.06) | 82.40(78.82, 85.98) | 80.08(75.74, 84.42) | 80.24(74.25, 86.24) | 91.97(87.45, 96.49) | 105.20(95.00, 115.40) | 97.74(86.63, 108.84) | 93.75(87.53, 99.98) | < 0.001 | 16.58(8.15, 25.01) | < 0.001 | 13.96(6.32, 21.61) |
| Nuts, mg | 53.06(45.98, 60.13) | 51.80(43.82, 59.78) | 54.34(48.72, 59.96) | 56.27(51.40, 61.13) | 62.89(55.79, 69.98) | 67.54(63.32, 71.76) | 64.29(58.28, 70.30) | 62.85(57.41, 68.28) | 69.68(60.37, 78.98) | 71.36(63.80, 78.93) | < 0.001 | 16.07(6.04, 26.10) | 0.010 | 11.77(1.87, 21.68) |
| Eggs, mg | 39.64(36.17, 43.10) | 38.91(35.62, 42.20) | 45.24(40.68, 49.81) | 43.38(40.44, 46.33) | 45.73(42.19, 49.27) | 47.36(42.69, 52.04) | 49.39(46.06, 52.72) | 51.92(47.10, 56.73) | 54.39(50.45, 58.33) | 60.89(56.45, 65.32) | < 0.001 | 21.49(14.43, 28.56) | < 0.001 | 13.58(6.49, 20.66) |
| Oils, mg | 3.07(2.68, 3.45) | 2.99(2.58, 3.40) | 3.08(2.74, 3.42) | 2.96(2.64, 3.28) | 2.62(2.40, 2.84) | 4.93(4.53, 5.33) | 5.88(5.09, 6.67) | 6.52(5.85, 7.19) | 6.05(4.72, 7.39) | 9.35(8.59, 10.10) | < 0.001 | 6.42(5.39, 7.44) | < 0.001 | 6.37(5.43, 7.32) |
| **Potassium** |  |  |  |  |  |  |  |  |  |  |  |  |  |  |
| DS, mg | 19.97(17.36, 22.58) | 19.01(16.58, 21.45) | 19.97(17.15, 22.80) | 19.84(17.70, 21.98) | 18.20(15.12, 21.29) | 17.64(15.63, 19.64) | 13.48(11.17, 15.78) | 13.42(11.96, 14.87) | 14.86(12.06, 17.67) | 13.42(11.09, 15.75) | < 0.001 | -8.60(-12.63, -4.58) | < 0.001 | -7.94(-11.66, -4.21) |
| Meats, mg | 518.04(497.54, 538.54) | 489.40(466.41, 512.40) | 491.94(472.47, 511.40) | 516.02(500.73, 531.31) | 524.04(507.08, 541.00) | 562.26(540.88, 583.64) | 527.56(511.33, 543.79) | 554.29(534.94, 573.63) | 538.10(518.35, 557.84) | 534.20(513.43, 554.97) | < 0.001 | 13.83(-19.46, 47.11) | 0.006 | 38.86(10.04, 67.69) |
| Grains, mg | 379.11(357.83, 400.38) | 388.10(371.73, 404.47) | 429.87(407.90, 451.84) | 427.39(415.07, 439.71) | 433.31(418.27, 448.36) | 435.42(417.72, 453.12) | 488.22(473.15, 503.30) | 469.80(459.08, 480.52) | 479.26(461.02, 497.49) | 427.32(412.43, 442.22) | < 0.001 | 40.78(16.13, 65.42) | 0.008 | -9.41(-33.28, 14.46) |
| Milks, mg | 398.74(369.92, 427.56) | 399.54(386.92, 412.15) | 359.24(330.47, 388.02) | 372.67(347.74, 397.60) | 332.11(306.09, 358.14) | 356.49(342.06, 370.91) | 306.76(284.85, 328.66) | 286.04(272.71, 299.36) | 289.82(267.76, 311.88) | 255.86(237.02, 274.70) | < 0.001 | -146.36(-181.70, -111.01) | < 0.001 | -97.42(-127.01, -67.84) |
| Vegetables, mg | 560.16(539.21, 581.11) | 519.46(488.00, 550.92) | 515.90(500.56, 531.24) | 512.52(482.60, 542.44) | 503.64(488.02, 519.26) | 502.42(483.59, 521.24) | 511.34(480.40, 542.28) | 480.14(457.52, 502.76) | 471.28(450.64, 491.92) | 497.70(469.88, 525.52) | < 0.001 | -71.51(-113.23, -29.79) | 0.011 | -18.51(-51.88, 14.86) |
| Fruits, mg | 310.24(268.15, 352.33) | 311.54(289.15, 333.92) | 285.84(250.80, 320.88) | 293.44(270.96, 315.91) | 293.95(263.77, 324.12) | 311.28(295.82, 326.74) | 297.13(272.19, 322.08) | 276.39(257.05, 295.73) | 272.52(250.87, 294.17) | 249.29(231.38, 267.19) | < 0.001 | -90.66(-136.60, -44.72) | < 0.001 | -53.73(-92.07, -15.38) |
| Beverages, mg | 315.55(297.78, 333.33) | 328.39(300.95, 355.83) | 323.82(309.55, 338.08) | 345.11(325.78, 364.45) | 323.89(301.86, 345.92) | 341.79(320.02, 363.56) | 352.16(327.46, 376.87) | 334.18(317.16, 351.20) | 336.13(317.59, 354.66) | 326.74(315.54, 337.94) | 0.039 | 13.28(-8.21, 34.76) | 0.399 | 5.80(-11.91, 23.51) |
| Nuts, mg | 106.85(93.39, 120.31) | 103.71(88.44, 118.98) | 105.15(94.91, 115.38) | 103.72(94.08, 113.35) | 113.67(100.84, 126.49) | 121.38(113.04, 129.72) | 123.72(112.19, 135.25) | 118.51(110.71, 126.31) | 130.17(115.51, 144.83) | 129.20(115.52, 142.89) | 0.007 | 15.62(-3.20, 34.45) | 0.029 | 12.28(-4.36, 28.93) |
| Eggs, mg | 31.79(28.97, 34.60) | 33.62(31.26, 35.98) | 38.18(33.79, 42.57) | 37.26(34.24, 40.27) | 37.07(34.45, 39.69) | 38.05(33.60, 42.51) | 40.45(37.36, 43.55) | 44.01(39.90, 48.13) | 46.29(42.86, 49.71) | 46.11(42.68, 49.53) | < 0.001 | 13.62(8.27, 18.97) | < 0.001 | 5.72(-0.10, 11.54) |
| Oils, mg | 6.44(5.39, 7.49) | 6.07(4.99, 7.14) | 5.62(5.12, 6.12) | 5.39(4.76, 6.02) | 5.33(4.83, 5.82) | 5.66(5.15, 6.17) | 6.25(5.68, 6.81) | 6.82(6.11, 7.52) | 6.17(5.48, 6.86) | 20.38(18.76, 21.99) | < 0.001 | 13.85(11.69, 16.01) | < 0.001 | 14.61(12.74, 16.49) |
| **Sodium** |  |  |  |  |  |  |  |  |  |  |  |  |  |  |
| DS, mg | 2.31(1.57, 3.04) | 2.69(1.98, 3.40) | 2.98(1.29, 4.68) | 4.02(3.15, 4.88) | 2.76(1.78, 3.74) | 2.34(1.54, 3.15) | 1.75(1.05, 2.45) | 2.70(2.13, 3.28) | 2.34(1.71, 2.98) | 3.06(2.26, 3.86) | 0.336 | 0.57(-0.64, 1.78) | 0.204 | -0.12(-2.04, 1.80) |
| Meats, mg | 965.45(920.19, 1010.72) | 929.42(882.67, 976.17) | 921.35(882.65, 960.06) | 945.28(896.30, 994.26) | 977.89(945.86, 1009.92) | 1098.20(1056.86, 1139.54) | 1044.33(1013.43, 1075.23) | 1039.84(1011.97, 1067.70) | 1038.47(993.00, 1083.93) | 1057.38(1012.03, 1102.74) | < 0.001 | 102.55(20.35, 184.76) | < 0.001 | 145.21(80.69, 209.74) |
| Grains, mg | 1150.47(1086.61, 1214.34) | 1171.97(1118.71, 1225.24) | 1229.37(1191.33, 1267.41) | 1200.27(1173.50, 1227.03) | 1202.90(1176.81, 1228.99) | 1179.17(1136.18, 1222.16) | 1237.58(1206.47, 1268.69) | 1218.34(1185.44, 1251.24) | 1228.87(1184.65, 1273.10) | 1107.33(1067.80, 1146.86) | 0.247 | -52.89(-127.70, 21.93) | 0.001 | -126.38(-185.14, -67.62) |
| Milks, mg | 278.81(261.53, 296.10) | 261.57(250.68, 272.45) | 256.95(242.14, 271.75) | 262.43(250.30, 274.57) | 258.38(242.44, 274.32) | 260.63(243.48, 277.78) | 221.37(205.70, 237.05) | 227.81(219.28, 236.35) | 236.38(218.57, 254.18) | 209.79(193.14, 226.44) | < 0.001 | -67.63(-94.47, -40.78) | < 0.001 | -43.72(-66.17, -21.27) |
| Vegetables, mg | 417.48(389.69, 445.26) | 385.83(355.24, 416.41) | 401.11(380.34, 421.87) | 409.25(386.57, 431.93) | 399.82(383.26, 416.37) | 442.18(423.54, 460.82) | 397.43(373.93, 420.93) | 415.61(394.76, 436.47) | 402.79(379.26, 426.31) | 395.64(379.23, 412.05) | 0.481 | -23.65(-63.14, 15.85) | 0.256 | -6.50(-33.00, 20.00) |
| Fruits, mg | 4.60(3.91, 5.29) | 4.91(4.55, 5.28) | 4.65(3.97, 5.33) | 4.78(4.06, 5.51) | 5.59(4.80, 6.37) | 5.97(5.24, 6.71) | 6.00(5.38, 6.63) | 5.36(4.51, 6.21) | 8.96(6.88, 11.03) | 10.67(8.61, 12.73) | < 0.001 | 5.43(3.01, 7.85) | < 0.001 | 5.62(3.17, 8.07) |
| Beverages, mg | 84.48(78.92, 90.05) | 76.70(71.85, 81.55) | 78.85(74.45, 83.26) | 119.06(114.32, 123.80) | 120.73(114.91, 126.54) | 116.54(112.90, 120.19) | 130.10(122.04, 138.16) | 139.43(132.70, 146.17) | 141.96(131.48, 152.44) | 134.98(126.92, 143.04) | < 0.001 | 56.73(45.81, 67.64) | < 0.001 | 59.47(48.91, 70.04) |
| Nuts, mg | 120.27(96.48, 144.06) | 109.84(95.03, 124.65) | 108.00(92.49, 123.50) | 104.24(94.50, 113.99) | 115.45(103.51, 127.38) | 124.58(115.75, 133.41) | 117.75(107.31, 128.19) | 101.08(93.59, 108.57) | 123.98(105.93, 142.03) | 127.13(115.32, 138.93) | 0.856 | -0.88(-25.96, 24.20) | 0.884 | 4.50(-14.56, 23.56) |
| Eggs, mg | 84.67(76.06, 93.27) | 80.77(71.22, 90.32) | 97.55(86.87, 108.23) | 93.51(87.16, 99.87) | 82.02(76.48, 87.56) | 87.40(78.75, 96.05) | 112.00(103.23, 120.77) | 120.28(109.21, 131.35) | 126.17(116.37, 135.96) | 105.33(97.34, 113.32) | < 0.001 | 19.84(5.18, 34.51) | < 0.001 | 3.14(-11.72, 17.99) |
| Oils, mg | 132.03(116.18, 147.87) | 125.18(111.40, 138.95) | 135.91(125.87, 145.96) | 131.61(115.77, 147.45) | 121.03(105.50, 136.56) | 98.01(90.30, 105.72) | 88.12(82.04, 94.21) | 95.32(87.35, 103.30) | 85.56(76.39, 94.74) | 110.01(102.39, 117.63) | < 0.001 | -29.69(-48.58, -10.79) | < 0.001 | -30.09(-42.38, -17.79) |
| **Zinc** |  |  |  |  |  |  |  |  |  |  |  |  |  |  |
| DS, mg | 5.51(4.87, 6.15) | 5.26(4.82, 5.69) | 5.48(4.93, 6.03) | 5.62(5.04, 6.19) | 4.82(4.37, 5.28) | 4.23(3.87, 4.58) | 3.92(3.50, 4.33) | 4.32(3.88, 4.76) | 3.97(3.58, 4.37) | 3.96(3.52, 4.41) | < 0.001 | -2.08(-2.92, -1.25) | < 0.001 | -1.81(-2.40, -1.21) |
| Meats, mg | 4.42(4.14, 4.70) | 4.32(4.09, 4.56) | 4.44(4.12, 4.76) | 4.79(4.32, 5.26) | 4.40(4.14, 4.67) | 4.29(4.03, 4.55) | 3.65(3.48, 3.82) | 3.63(3.50, 3.76) | 3.69(3.46, 3.92) | 3.67(3.52, 3.82) | < 0.001 | -0.69(-1.05, -0.33) | < 0.001 | -0.79(-1.18, -0.39) |
| Grains, mg | 2.92(2.73, 3.10) | 3.30(3.09, 3.52) | 3.42(3.21, 3.63) | 3.57(3.44, 3.71) | 3.60(3.50, 3.70) | 3.51(3.39, 3.63) | 3.68(3.55, 3.82) | 3.59(3.44, 3.74) | 3.57(3.41, 3.74) | 3.10(2.98, 3.22) | 0.097 | 0.18(-0.05, 0.41) | 0.009 | -0.33(-0.56, -0.10) |
| Milks, mg | 1.52(1.41, 1.64) | 1.61(1.54, 1.69) | 1.45(1.35, 1.54) | 1.53(1.45, 1.61) | 1.44(1.35, 1.54) | 1.50(1.43, 1.58) | 1.23(1.15, 1.30) | 1.23(1.18, 1.28) | 1.22(1.13, 1.31) | 1.06(0.98, 1.14) | < 0.001 | -0.48(-0.64, -0.33) | < 0.001 | -0.37(-0.48, -0.25) |
| Vegetables, mg | 0.66(0.61, 0.70) | 0.62(0.58, 0.67) | 0.71(0.67, 0.75) | 0.71(0.66, 0.76) | 0.70(0.67, 0.73) | 0.64(0.61, 0.67) | 0.63(0.60, 0.67) | 0.62(0.58, 0.66) | 0.57(0.54, 0.60) | 0.58(0.54, 0.62) | < 0.001 | -0.1(-0.16, -0.03) | < 0.001 | -0.13(-0.19, -0.07) |
| Fruits, mg | 0.14(0.12, 0.16) | 0.15(0.14, 0.16) | 0.14(0.12, 0.16) | 0.15(0.14, 0.16) | 0.16(0.14, 0.17) | 0.17(0.16, 0.18) | 0.16(0.15, 0.18) | 0.16(0.15, 0.18) | 0.17(0.15, 0.19) | 0.16(0.15, 0.18) | 0.275 | 0.00(-0.02, 0.03) | 0.329 | 0.01(-0.01, 0.03) |
| Beverages, mg | 0.49(0.46, 0.51) | 0.34(0.31, 0.36) | 0.37(0.35, 0.39) | 0.46(0.45, 0.48) | 0.46(0.44, 0.48) | 0.46(0.43, 0.48) | 0.58(0.54, 0.62) | 0.74(0.67, 0.81) | 0.76(0.66, 0.87) | 0.69(0.64, 0.73) | < 0.001 | 0.21(0.15, 0.28) | < 0.001 | 0.32(0.27, 0.38) |
| Nuts, mg | 0.52(0.46, 0.59) | 0.53(0.46, 0.60) | 0.59(0.51, 0.66) | 0.56(0.51, 0.61) | 0.61(0.54, 0.68) | 0.62(0.57, 0.68) | 0.53(0.48, 0.58) | 0.49(0.45, 0.54) | 0.55(0.47, 0.63) | 0.54(0.48, 0.59) | 0.098 | 0.00(-0.10, 0.10) | 0.004 | -0.08(-0.18, 0.02) |
| Eggs, mg | 0.25(0.22, 0.27) | 0.23(0.22, 0.25) | 0.27(0.24, 0.30) | 0.26(0.24, 0.28) | 0.27(0.25, 0.29) | 0.30(0.27, 0.33) | 0.31(0.29, 0.34) | 0.34(0.30, 0.37) | 0.36(0.34, 0.38) | 0.39(0.36, 0.41) | < 0.001 | 0.14(0.10, 0.18) | < 0.001 | 0.10(0.06, 0.15) |
| Oils, mcg | 15.91(13.55, 18.26) | 17.04(14.37, 19.71) | 17.88(16.02, 19.74) | 17.97(15.83, 20.12) | 16.41(14.90, 17.92) | 21.26(19.40, 23.12) | 17.65(14.25, 21.05) | 15.64(13.25, 18.03) | 13.86(11.89, 15.83) | 42.84(38.44, 47.24) | < 0.001 | 26.33(20.82, 31.85) | < 0.001 | 24.16(18.92, 29.41) |
| **Copper** |  |  |  |  |  |  |  |  |  |  |  |  |  |  |
| DS, mg | 0.56(0.51, 0.62) | 0.58(0.53, 0.63) | 0.57(0.51, 0.64) | 0.59(0.54, 0.64) | 0.45(0.40, 0.50) | 0.32(0.29, 0.35) | 0.28(0.25, 0.31) | 0.30(0.27, 0.33) | 0.27(0.24, 0.30) | 0.23(0.20, 0.25) | < 0.001 | -0.38(-0.45, -0.32) | < 0.001 | -0.37(-0.43, -0.31) |
| Meats, mg | 0.22(0.20, 0.24) | 0.26(0.21, 0.30) | 0.22(0.19, 0.25) | 0.26(0.23, 0.28) | 0.24(0.22, 0.25) | 0.23(0.21, 0.25) | 0.21(0.17, 0.26) | 0.18(0.17, 0.19) | 0.20(0.16, 0.23) | 0.19(0.17, 0.20) | < 0.001 | -0.04(-0.07, -0.01) | < 0.001 | -0.04(-0.08, 0) |
| Grains, mg | 0.34(0.33, 0.36) | 0.39(0.37, 0.40) | 0.40(0.38, 0.42) | 0.40(0.39, 0.40) | 0.40(0.38, 0.41) | 0.37(0.36, 0.38) | 0.38(0.38, 0.39) | 0.37(0.36, 0.37) | 0.36(0.35, 0.38) | 0.33(0.32, 0.34) | < 0.001 | -0.02(-0.05, 0.00) | < 0.001 | -0.08(-0.10, -0.06) |
| Milks, mg | 0.07(0.06, 0.08) | 0.08(0.06, 0.09) | 0.07(0.06, 0.07) | 0.08(0.07, 0.09) | 0.07(0.06, 0.07) | 0.08(0.07, 0.09) | 0.06(0.06, 0.06) | 0.06(0.06, 0.06) | 0.06(0.05, 0.06) | 0.04(0.04, 0.04) | < 0.001 | -0.03(-0.05, -0.01) | < 0.001 | -0.03(-0.03, -0.02) |
| Vegetables, mg | 0.21(0.20, 0.22) | 0.18(0.17, 0.20) | 0.18(0.17, 0.19) | 0.18(0.17, 0.19) | 0.18(0.17, 0.18) | 0.17(0.16, 0.18) | 0.15(0.14, 0.16) | 0.14(0.14, 0.15) | 0.14(0.14, 0.15) | 0.14(0.13, 0.15) | < 0.001 | -0.07(-0.09, -0.06) | < 0.001 | -0.04(-0.05, -0.03) |
| Fruits, mg | 0.10(0.08, 0.11) | 0.10(0.09, 0.11) | 0.09(0.08, 0.10) | 0.09(0.08, 0.10) | 0.08(0.08, 0.09) | 0.09(0.09, 0.10) | 0.09(0.08, 0.09) | 0.08(0.08, 0.09) | 0.08(0.07, 0.09) | 0.08(0.07, 0.09) | < 0.001 | -0.02(-0.04, -0.01) | < 0.001 | -0.01(-0.03, 0.00) |
| Beverages, mg | 0.17(0.16, 0.18) | 0.14(0.13, 0.15) | 0.09(0.09, 0.10) | 0.19(0.18, 0.20) | 0.19(0.18, 0.19) | 0.18(0.18, 0.19) | 0.21(0.20, 0.22) | 0.21(0.20, 0.23) | 0.23(0.21, 0.24) | 0.21(0.20, 0.22) | < 0.001 | 0.05(0.03, 0.06) | < 0.001 | 0.12(0.11, 0.13) |
| Nuts, mg | 0.11(0.09, 0.12) | 0.11(0.09, 0.13) | 0.11(0.10, 0.12) | 0.12(0.11, 0.13) | 0.13(0.11, 0.14) | 0.14(0.13, 0.15) | 0.13(0.12, 0.15) | 0.13(0.11, 0.14) | 0.14(0.11, 0.16) | 0.14(0.12, 0.16) | 0.009 | 0.03(0.00, 0.06) | 0.129 | 0.02(0.00, 0.04) |
| Eggs, mcg | 4.93(4.34, 5.52) | 17.07(15.77, 18.37) | 19.43(17.41, 21.45) | 19.38(18.09, 20.66) | 21.83(20.16, 23.50) | 17.63(15.93, 19.32) | 18.99(17.46, 20.52) | 19.51(17.70, 21.32) | 20.33(18.56, 22.10) | 17.57(15.56, 19.57) | < 0.001 | 12.56(9.96, 15.16) | 0.085 | -2.47(-5.59, 0.65) |
| Oils, mcg | 1.89(0.36, 3.42) | 1.39(1.11, 1.68) | 1.29(1.09, 1.49) | 1.72(1.40, 2.03) | 1.43(1.21, 1.64) | 2.37(2.14, 2.61) | 2.47(1.90, 3.04) | 2.07(1.71, 2.43) | 1.75(1.48, 2.02) | 5.64(5.11, 6.17) | < 0.001 | 3.74(1.88, 5.59) | < 0.001 | 4.37(3.68, 5.05) |
| **Selenium** |  |  |  |  |  |  |  |  |  |  |  |  |  |  |
| DS, mg | 15.28(12.68, 17.89) | 18.13(15.20, 21.06) | 17.71(15.52, 19.89) | 18.27(15.59, 20.96) | 14.42(13.02, 15.82) | 16.66(15.64, 17.68) | 15.26(13.51, 17.01) | 16.26(14.35, 18.18) | 12.69(10.29, 15.10) | 12.44(11.05, 13.83) | < 0.001 | -4.48(-7.78, -1.19) | < 0.001 | -5.97(-8.50, -3.45) |
| Meats, mg | 39.85(38.22, 41.49) | 36.65(35.14, 38.15) | 39.20(37.59, 40.81) | 42.79(41.50, 44.07) | 42.20(40.72, 43.68) | 44.71(42.78, 46.65) | 42.36(40.69, 44.03) | 45.25(43.28, 47.23) | 43.69(41.57, 45.81) | 44.01(42.30, 45.73) | < 0.001 | 3.88(0.98, 6.78) | 0.002 | 4.60(2.11, 7.09) |
| Grains, mg | 39.10(37.04, 41.16) | 39.23(37.39, 41.08) | 40.63(38.60, 42.66) | 40.30(39.43, 41.18) | 41.83(40.54, 43.11) | 41.66(40.18, 43.14) | 44.55(43.36, 45.74) | 43.74(42.64, 44.83) | 42.57(40.99, 44.15) | 38.33(37.08, 39.58) | 0.101 | -1.23(-3.78, 1.32) | 0.501 | -2.67(-5.14, -0.19) |
| Milks, mg | 7.55(6.99, 8.11) | 9.12(8.80, 9.43) | 8.42(7.87, 8.98) | 8.65(8.16, 9.14) | 8.18(7.59, 8.76) | 8.50(8.06, 8.94) | 7.20(6.77, 7.63) | 8.51(8.14, 8.89) | 8.92(8.27, 9.57) | 6.79(6.19, 7.39) | 0.005 | -0.82(-1.69, 0.04) | 0.002 | -1.54(-2.29, -0.80) |
| Vegetables, mg | 2.64(2.41, 2.88) | 2.42(2.22, 2.63) | 2.64(2.48, 2.79) | 2.68(2.48, 2.88) | 2.59(2.44, 2.74) | 2.44(2.30, 2.58) | 2.43(2.28, 2.58) | 2.57(2.39, 2.75) | 2.20(2.01, 2.39) | 2.48(2.30, 2.65) | 0.004 | -0.28(-0.61, 0.05) | 0.002 | -0.18(-0.43, 0.08) |
| Fruits, mg | 0.55(0.47, 0.64) | 0.44(0.41, 0.46) | 0.41(0.36, 0.46) | 0.43(0.39, 0.48) | 0.45(0.40, 0.50) | 0.48(0.46, 0.50) | 0.47(0.43, 0.50) | 0.47(0.43, 0.52) | 0.48(0.41, 0.54) | 0.51(0.44, 0.57) | 0.453 | -0.10(-0.21, 0.00) | 0.223 | 0.06(-0.02, 0.14) |
| Beverages, mg | 3.09(2.57, 3.60) | 1.96(1.75, 2.17) | 1.74(1.61, 1.86) | 1.67(1.50, 1.84) | 1.58(1.52, 1.65) | 1.76(1.59, 1.93) | 2.04(1.87, 2.20) | 2.40(2.09, 2.71) | 2.60(2.12, 3.08) | 2.12(1.88, 2.36) | 0.269 | -0.84(-1.42, -0.26) | < 0.001 | 0.41(0.14, 0.69) |
| Nuts, mg | 3.53(2.30, 4.77) | 3.83(2.40, 5.26) | 3.15(2.69, 3.61) | 2.52(2.20, 2.84) | 3.45(2.89, 4.01) | 2.34(2.11, 2.58) | 3.15(2.32, 3.98) | 2.93(2.32, 3.55) | 3.55(2.92, 4.18) | 4.34(3.56, 5.12) | 0.828 | 0.62(-1.22, 2.45) | 0.035 | 0.92(-0.28, 2.12) |
| Eggs, mg | 5.94(5.40, 6.48) | 5.85(5.42, 6.27) | 6.46(5.76, 7.16) | 6.16(5.67, 6.66) | 6.44(6.02, 6.86) | 6.33(5.70, 6.96) | 6.57(6.17, 6.96) | 7.25(6.61, 7.90) | 7.76(7.22, 8.31) | 8.84(8.18, 9.49) | < 0.001 | 2.82(1.77, 3.87) | < 0.001 | 2.06(1.03, 3.09) |
| Oils, mg | 0.18(0.16, 0.21) | 0.18(0.15, 0.21) | 0.18(0.16, 0.20) | 0.18(0.16, 0.20) | 0.19(0.17, 0.21) | 0.20(0.18, 0.23) | 0.21(0.19, 0.24) | 0.22(0.20, 0.24) | 0.20(0.17, 0.24) | 0.46(0.40, 0.51) | < 0.001 | 0.27(0.20, 0.33) | < 0.001 | 0.27(0.21, 0.33) |

Abbreviations: NHANES, National Health and Nutrition Examination Survey; DSs, Dietary supplements; Milks, Milk and milk products; Meats, Meat, poultry, fish and mixtures; Eggs, Eggs; Nuts, Legumes, nuts and seeds; Grains, Grain products; Fruits, Fruits; Vegetables, Vegetables; Oils, Fats, oils and salad dressings; Beverages, Sugar, sweeteners, and beverages.

a Data were adjusted for NHANES survey weights, and were adjusted for total energy intake to 2000 kcal/d using the residual method.

b Results were adjusted for gender, age, race/ethnicity, education level, and family income.

c 1999-2000 vs. 2017-2020, and differences were adjusted for gender, age, race/ethnicity, education level, and family income.

d 2003-2004 vs. 2017-2020, and differences were adjusted for gender, age, race/ethnicity, education level, and family income.

**Supplementary Table 4.** Changes in Estimate Intake of Nine Minerals by Sociodemographic Characteristics by NHANES Survey Cycle, 1999-2020

|  | Survey-Weighted Mean (95% CI) ^a^ | | | 1999-2020 | | 2003-2020 | |
| --- | --- | --- | --- | --- | --- | --- | --- |
|  | 1999-2000(n=3745) | | 2017-2020(n=7332) | FDR for Trend ^b^ | Difference (95% CI) ^c^ | FDR for Trend ^b^ | Difference (95% CI) ^d^ |
| **Gender** |  | |  |  |  |  |  |
| **Male** |  | |  |  |  |  |  |
| **FB ^e^ + DSs** |  | |  |  |  |  |  |
| Calcium, g | 0.85(0.81, 0.90) | | 0.97(0.94, 0.99) | < 0.001 | 0.11(0.06, 0.16) | 0.002 | 0.06(0.01, 0.11) |
| Iron, mg | 18.02(17.03, 19.01) | | 15.46(14.78, 16.13) | < 0.001 | -3.36(-4.64, -2.08) | < 0.001 | -3.31(-4.48, -2.14) |
| Magnesium, mg | 308.98(298.86, 319.10) | | 321.35(311.84, 330.87) | < 0.001 | -1.40(-14.29, 11.49) | 0.008 | 17.95(6.77, 29.14) |
| Phosphorus, g | 1.25(1.22, 1.28) | | 1.32(1.30, 1.34) | < 0.001 | 0.06(0.03, 0.09) | < 0.001 | 0.05(0.01, 0.09) |
| Potassium, g | 2.69(2.63, 2.74) | | 2.50(2.45, 2.55) | < 0.001 | -0.26(-0.33, -0.19) | < 0.001 | -0.12(-0.19, -0.04) |
| Sodium, g | 3.30(3.20, 3.39) | | 3.34(3.29, 3.38) | < 0.001 | 0.01(-0.13, 0.15) | 0.081 | 0.02(-0.07, 0.11) |
| Zinc, mg | 16.06(15.05, 17.07) | | 14.40(13.79, 15.01) | < 0.001 | -2.45(-3.66, -1.25) | < 0.001 | -2.77(-3.77, -1.76) |
| Copper, mg | 1.75(1.65, 1.84) | | 1.35(1.30, 1.40) | < 0.001 | -0.49(-0.60, -0.38) | < 0.001 | -0.49(-0.57, -0.41) |
| Selenium, mcg | 120.42(115.94, 124.90) | | 128.29(125.61, 130.98) | < 0.001 | 3.86(-1.32, 9.05) | 0.075 | 1.08(-3.76, 5.93) |
| **FB ^e^** | | |  |  |  |  |  |
| Calcium, g | 0.76(0.72, 0.79) | | 0.89(0.86, 0.91) | < 0.001 | 0.14(0.10, 0.18) | < 0.001 | 0.11(0.07, 0.16) |
| Iron, mg | 14.57(14.01, 15.14) | | 13.50(13.13, 13.88) | < 0.001 | -1.46(-2.13, -0.80) | < 0.001 | -1.89(-2.53, -1.26) |
| Magnesium, mg | 275.77(269.54, 282.01) | | 290.74(284.72, 296.76) | < 0.001 | 6.75(-1.50, 14.99) | < 0.001 | 24.90(16.03, 33.77) |
| Phosphorus, g | 1.22(1.20, 1.25) | | 1.31(1.30, 1.33) | < 0.001 | 0.08(0.05, 0.11) | < 0.001 | 0.07(0.03, 0.11) |
| Potassium, g | 2.67(2.61, 2.72) | | 2.49(2.44, 2.53) | < 0.001 | -0.25(-0.32, -0.18) | < 0.001 | -0.11(-0.18, -0.03) |
| Sodium, g | 3.30(3.20, 3.39) | | 3.33(3.28, 3.38) | < 0.001 | 0.01(-0.13, 0.15) | 0.080 | 0.02(-0.07, 0.11) |
| Zinc, mg | 11.18(10.85, 11.51) | | 10.48(10.34, 10.62) | < 0.001 | -0.81(-1.19, -0.44) | < 0.001 | -1.30(-1.88, -0.72) |
| Copper, mg | 1.24(1.21, 1.28) | | 1.12(1.08, 1.16) | < 0.001 | -0.15(-0.21, -0.10) | < 0.001 | -0.09(-0.16, -0.02) |
| Selenium, mcg | 106.30(102.73, 109.87) | | 112.39(110.52, 114.26) | < 0.001 | 4.39(-0.11, 8.89) | < 0.001 | 6.24(2.96, 9.52) |
| **Female** |  | |  |  |  |  |  |
| **FB ^e^ + DSs** | | |  |  |  |  |  |
| Calcium, g | 1.01(0.97, 1.05) | | 1.07(1.03, 1.10) | 0.714 | 0.04(-0.02, 0.10) | < 0.001 | -0.07(-0.12, -0.02) |
| Iron, mg | 20.22(19.31, 21.12) | | 17.22(16.64, 17.81) | < 0.001 | -3.27(-4.50, -2.04) | < 0.001 | -2.56(-3.89, -1.24) |
| Magnesium, mg | 307.25(292.12, 322.39) | | 322.31(312.04, 332.58) | 0.126 | 6.51(-12.99, 26) | 0.822 | 12.30(-1.89, 26.50) |
| Phosphorus, g | 1.24(1.21, 1.26) | | 1.29(1.28, 1.30) | < 0.001 | 0.05(0.01, 0.09) | 0.024 | 0.03(0.00, 0.06) |
| Potassium, g | 2.61(2.52, 2.71) | | 2.50(2.45, 2.55) | < 0.001 | -0.16(-0.27, -0.05) | < 0.001 | -0.10(-0.18, -0.03) |
| Sodium, g | 3.19(3.12, 3.26) | | 3.19(3.15, 3.24) | 0.017 | 0.01(-0.10, 0.12) | 0.811 | 0.00(-0.08, 0.08) |
| Zinc, mg | 16.80(15.90, 17.70) | | 14.00(13.53, 14.46) | < 0.001 | -3.07(-4.21, -1.94) | < 0.001 | -3.28(-4.29, -2.28) |
| Copper, mg | 1.83(1.76, 1.90) | | 1.40(1.36, 1.45) | < 0.001 | -0.48(-0.58, -0.39) | < 0.001 | -0.36(-0.47, -0.26) |
| Selenium, mcg | 115.29(111.20, 119.39) | | 113.02(111.25, 114.79) | 0.053 | -3.73(-9.46, 2.00) | 0.012 | -4.96(-9.73, -0.20) |
| **FB ^e^** | |  |  |  |  |  |  |
| Calcium, g | 0.80(0.76, 0.83) | | 0.90(0.88, 0.92) | < 0.001 | 0.11(0.06, 0.15) | 0.037 | 0.06(0.03, 0.09) |
| Iron, mg | 14.43(13.92, 14.93) | | 13.12(12.96, 13.29) | < 0.001 | -1.24(-1.82, -0.66) | < 0.001 | -1.64(-2.06, -1.22) |
| Magnesium, mg | 266.57(255.11, 278.02) | | 289.10(282.45, 295.74) | < 0.001 | 17.80(4.27, 31.33) | 0.002 | 22.21(13.02, 31.39) |
| Phosphorus, g | 1.21(1.18, 1.24) | | 1.28(1.27, 1.30) | < 0.001 | 0.07(0.03, 0.11) | < 0.001 | 0.04(0.02, 0.07) |
| Potassium, g | 2.59(2.50, 2.69) | | 2.49(2.43, 2.54) | 0.001 | -0.15(-0.26, -0.04) | < 0.001 | -0.10(-0.17, -0.02) |
| Sodium, g | 3.19(3.12, 3.26) | | 3.19(3.14, 3.24) | 0.010 | 0.01(-0.10, 0.12) | 0.693 | 0.00(-0.08, 0.08) |
| Zinc, mg | 10.72(10.30, 11.13) | | 10.00(9.87, 10.13) | < 0.001 | -0.60(-1.10, -0.09) | < 0.001 | -1.17(-1.69, -0.66) |
| Copper, mg | 1.21(1.17, 1.25) | | 1.18(1.14, 1.22) | 0.010 | -0.06(-0.11, 0.00) | < 0.001 | -0.02(-0.08, 0.03) |
| Selenium, mcg | 98.95(96.55, 101.36) | | 103.74(102.62, 104.87) | < 0.001 | 4.18(1.33, 7.02) | 0.002 | 1.72(-1.15, 4.59) |
| **Age group, y** |  | |  |  |  |  |  |
| **20-34y** |  | |  |  |  |  |  |
| **FB ^e^ + DSs** | | |  |  |  |  |  |
| Calcium, g | 0.80(0.75, 0.85) | | 0.95(0.93, 0.98) | < 0.001 | 0.16(0.10, 0.22) | 0.356 | 0.04(-0.01, 0.09) |
| Iron, mg | 16.98(15.33, 18.62) | | 15.40(14.85, 15.95) | < 0.001 | -1.46(-3.33, 0.40) | < 0.001 | -2.47(-4.16, -0.77) |
| Magnesium, mg | 255.02(244.95, 265.08) | | 288.76(276.30, 301.23) | < 0.001 | 33.83(17.46, 50.20) | 0.025 | 25.94(7.80, 44.08) |
| Phosphorus, g | 1.17(1.13, 1.20) | | 1.28(1.26, 1.31) | < 0.001 | 0.12(0.07, 0.17) | < 0.001 | 0.05(0.01, 0.10) |
| Potassium, g | 2.29(2.19, 2.39) | | 2.29(2.23, 2.36) | 0.210 | 0.01(-0.12, 0.15) | 0.918 | 0.00(-0.10, 0.10) |
| Sodium, g | 3.21(3.09, 3.32) | | 3.39(3.33, 3.45) | < 0.001 | 0.18(0.01, 0.34) | 0.001 | 0.09(-0.02, 0.21) |
| Zinc, mg | 13.56(12.19, 14.92) | | 11.67(11.20, 12.15) | < 0.001 | -1.82(-3.35, -0.29) | < 0.001 | -2.91(-3.99, -1.82) |
| Copper, mg | 1.43(1.32, 1.54) | | 1.20(1.14, 1.25) | < 0.001 | -0.24(-0.35, -0.13) | < 0.001 | -0.25(-0.36, -0.15) |
| Selenium, mcg | 105.81(99.98, 111.64) | | 116.31(113.16, 119.45) | < 0.001 | 9.82(2.95, 16.70) | 0.055 | 4.10(-2.57, 10.76) |
| **FB ^e^** | | |  |  |  |  |  |
| Calcium, g | 0.73(0.68, 0.78) | | 0.90(0.87, 0.93) | < 0.001 | 0.18(0.12, 0.24) | < 0.001 | 0.09(0.04, 0.13) |
| Iron, mg | 13.35(12.56, 14.14) | | 13.19(12.73, 13.65) | 0.006 | -0.11(-1.00, 0.78) | < 0.001 | -1.18(-2.02, -0.34) |
| Magnesium, mg | 234.63(224.29, 244.96) | | 276.32(265.45, 287.19) | < 0.001 | 41.33(25.17, 57.49) | < 0.001 | 32.90(17.57, 48.24) |
| Phosphorus, g | 1.15(1.12, 1.19) | | 1.28(1.26, 1.31) | < 0.001 | 0.14(0.09, 0.18) | < 0.001 | 0.06(0.02, 0.11) |
| Potassium, g | 2.28(2.18, 2.38) | | 2.29(2.22, 2.35) | 0.155 | 0.02(-0.11, 0.16) | 0.787 | 0.01(-0.09, 0.10) |
| Sodium, g | 3.21(3.09, 3.32) | | 3.39(3.32, 3.45) | < 0.001 | 0.18(0.01, 0.34) | 0.001 | 0.09(-0.03, 0.21) |
| Zinc, mg | 10.16(9.63, 10.69) | | 10.14(9.92, 10.37) | 0.010 | 0.16(-0.43, 0.75) | < 0.001 | -1.05(-1.65, -0.46) |
| Copper, mg | 1.12(1.06, 1.18) | | 1.08(1.03, 1.13) | 0.016 | -0.04(-0.10, 0.03) | 0.006 | -0.03(-0.09, 0.03) |
| Selenium, mcg | 96.75(91.73, 101.76) | | 110.02(107.66, 112.38) | < 0.001 | 12.70(6.79, 18.62) | < 0.001 | 8.69(4.66, 12.73) |
| **35-49y** |  | |  |  |  |  |  |
| **FB ^e^ + DSs** | | |  |  |  |  |  |
| Calcium, g | 0.93(0.87, 1.00) | | 0.99(0.95, 1.04) | < 0.001 | 0.06(-0.02, 0.14) | 0.826 | 0.05(-0.03, 0.12) |
| Iron, mg | 19.66(18.34, 20.98) | | 16.05(15.22, 16.88) | < 0.001 | -4.73(-6.39, -3.07) | < 0.001 | -2.79(-4.36, -1.23) |
| Magnesium, mg | 317.73(298.62, 336.83) | | 312.58(303.17, 321.99) | 0.151 | -15.66(-40.51, 9.18) | 0.150 | 21.74(7.02, 36.45) |
| Phosphorus, g | 1.25(1.21, 1.30) | | 1.30(1.28, 1.33) | < 0.001 | 0.04(-0.01, 0.100) | < 0.001 | 0.08(0.04, 0.13) |
| Potassium, g | 2.64(2.55, 2.74) | | 2.45(2.39, 2.51) | 0.001 | -0.24(-0.35, -0.13) | 0.017 | -0.11(-0.20, -0.01) |
| Sodium, g | 3.19(3.08, 3.31) | | 3.27(3.20, 3.35) | 0.001 | 0.07(-0.09, 0.23) | 0.132 | 0.09(-0.03, 0.20) |
| Zinc, mg | 16.30(14.92, 17.68) | | 12.91(12.23, 13.60) | < 0.001 | -3.87(-5.53, -2.22) | < 0.001 | -2.64(-3.95, -1.33) |
| Copper, mg | 1.83(1.71, 1.96) | | 1.32(1.25, 1.39) | < 0.001 | -0.59(-0.75, -0.43) | < 0.001 | -0.29(-0.41, -0.17) |
| Selenium, mcg | 119.72(110.98, 128.45) | | 118.93(114.68, 123.18) | 0.501 | -3.98(-15.13, 7.18) | 0.826 | 3.13(-3.24, 9.49) |
| **FB ^e^** | | |  |  |  |  |  |
| Calcium, g | 0.79(0.74, 0.84) | | 0.90(0.86, 0.93) | < 0.001 | 0.11(0.06, 0.17) | < 0.001 | 0.12(0.07, 0.18) |
| Iron, mg | 13.98(13.35, 14.61) | | 12.83(12.42, 13.24) | < 0.001 | -1.36(-2.17, -0.55) | < 0.001 | -1.67(-2.50, -0.84) |
| Magnesium, mg | 277.64(266.94, 288.35) | | 288.14(282.11, 294.18) | < 0.001 | 3.90(-9.64, 17.43) | < 0.001 | 28.60(19.21, 37.98) |
| Phosphorus, g | 1.22(1.18, 1.26) | | 1.30(1.27, 1.33) | < 0.001 | 0.07(0.03, 0.11) | < 0.001 | 0.10(0.05, 0.14) |
| Potassium, g | 2.62(2.52, 2.72) | | 2.44(2.38, 2.50) | 0.003 | -0.23(-0.33, -0.12) | 0.020 | -0.10(-0.19, -0.01) |
| Sodium, g | 3.19(3.07, 3.31) | | 3.27(3.20, 3.34) | 0.001 | 0.07(-0.10, 0.23) | 0.093 | 0.09(-0.03, 0.20) |
| Zinc, mg | 10.98(10.42, 11.53) | | 10.29(10.08, 10.50) | 0.004 | -0.75(-1.38, -0.13) | < 0.001 | -0.68(-1.31, -0.05) |
| Copper, mg | 1.26(1.22, 1.31) | | 1.12(1.08, 1.15) | 0.003 | -0.17(-0.25, -0.10) | < 0.001 | -0.04(-0.09, 0.00) |
| Selenium, mcg | 101.66(97.36, 105.96) | | 107.62(105.10, 110.15) | < 0.001 | 4.81(-0.40, 10.03) | 0.002 | 7.42(3.46, 11.37) |
| **50-64y** |  | |  |  |  |  |  |
| **FB ^e^ + DSs** | | |  |  |  |  |  |
| Calcium, g | 1.01(0.94, 1.08) | | 1.01(0.97, 1.06) | 0.792 | 0.02(-0.08, 0.11) | 0.042 | -0.04(-0.13, 0.04) |
| Iron, mg | 20.08(18.48, 21.68) | | 16.03(15.14, 16.92) | < 0.001 | -3.66(-5.96, -1.37) | < 0.001 | -3.01(-5.04, -0.99) |
| Magnesium, mg | 337.47(315.91, 359.02) | | 331.94(314.31, 349.56) | 0.792 | -7.51(-39.70, 24.68) | 0.382 | 0.87(-23.07, 24.80) |
| Phosphorus, g | 1.26(1.25, 1.28) | | 1.31(1.28, 1.34) | < 0.001 | 0.04(0.00, 0.07) | 0.136 | 0.03(-0.02, 0.07) |
| Potassium, g | 2.88(2.79, 2.98) | | 2.59(2.50, 2.67) | < 0.001 | -0.33(-0.47, -0.18) | < 0.001 | -0.16(-0.28, -0.04) |
| Sodium, g | 3.30(3.18, 3.42) | | 3.19(3.11, 3.27) | 0.900 | -0.13(-0.31, 0.05) | 0.136 | -0.12(-0.26, 0.01) |
| Zinc, mg | 18.80(17.12, 20.47) | | 14.30(13.51, 15.09) | < 0.001 | -4.08(-5.89, -2.28) | < 0.001 | -3.87(-5.60, -2.13) |
| Copper, mg | 2.00(1.83, 2.17) | | 1.40(1.34, 1.45) | < 0.001 | -0.61(-0.80, -0.41) | < 0.001 | -0.55(-0.68, -0.43) |
| Selenium, mcg | 127.51(120.75, 134.26) | | 121.87(117.18, 126.55) | 0.352 | -5.62(-15.01, 3.78) | 0.105 | -8.25(-15.99, -0.51) |
| **FB ^e^** | | |  |  |  |  |  |
| Calcium, g | 0.77(0.73, 0.80) | | 0.88(0.85, 0.91) | < 0.001 | 0.12(0.05, 0.18) | 0.002 | 0.10(0.04, 0.16) |
| Iron, mg | 15.13(14.16, 16.10) | | 13.25(12.83, 13.66) | < 0.001 | -1.82(-2.96, -0.68) | < 0.001 | -2.04(-2.86, -1.23) |
| Magnesium, mg | 286.72(275.90, 297.55) | | 297.31(288.35, 306.26) | < 0.001 | 6.94(-9.50, 23.38) | 0.062 | 20.13(7.52, 32.74) |
| Phosphorus, g | 1.23(1.22, 1.25) | | 1.30(1.27, 1.33) | < 0.001 | 0.06(0.02, 0.09) | 0.009 | 0.04(-0.01, 0.09) |
| Potassium, g | 2.86(2.76, 2.95) | | 2.57(2.49, 2.65) | < 0.001 | -0.32(-0.46, -0.17) | < 0.001 | -0.15(-0.27, -0.03) |
| Sodium, g | 3.30(3.18, 3.42) | | 3.19(3.11, 3.27) | 0.880 | -0.13(-0.32, 0.05) | 0.138 | -0.12(-0.26, 0.02) |
| Zinc, mg | 11.44(10.75, 12.13) | | 10.23(9.97, 10.49) | < 0.001 | -1.33(-2.14, -0.51) | < 0.001 | -1.36(-1.93, -0.79) |
| Copper, mg | 1.26(1.19, 1.32) | | 1.18(1.14, 1.22) | 0.001 | -0.09(-0.18, 0.00) | < 0.001 | -0.06(-0.13, 0.02) |
| Selenium, mcg | 109.51(104.43, 114.59) | | 107.95(105.02, 110.88) | 0.039 | -1.90(-8.76, 4.97) | 0.372 | -0.94(-5.70, 3.82) |
| **≥65y** |  | |  |  |  |  |  |
| **FB ^e^ + DSs** | | |  |  |  |  |  |
| Calcium, g | 1.08(1.02, 1.13) | | 1.13(1.09, 1.17) | 0.936 | 0.03(-0.06, 0.12) | < 0.001 | -0.10(-0.16, -0.05) |
| Iron, mg | 20.88(19.39, 22.36) | | 18.33(17.23, 19.43) | < 0.001 | -2.98(-5.37, -0.59) | < 0.001 | -3.53(-5.16, -1.91) |
| Magnesium, mg | 343.98(332.77, 355.18) | | 359.91(344.56, 375.26) | 0.331 | 5.50(-14.22, 25.22) | 0.537 | 14.18(-4.17, 32.53) |
| Phosphorus, g | 1.31(1.29, 1.33) | | 1.32(1.30, 1.34) | 0.326 | -0.01(-0.03, 0.01) | 0.990 | 0.00(-0.03, 0.02) |
| Potassium, g | 2.96(2.89, 3.04) | | 2.70(2.65, 2.76) | < 0.001 | -0.30(-0.40, -0.19) | < 0.001 | -0.17(-0.26, -0.08) |
| Sodium, g | 3.31(3.24, 3.38) | | 3.18(3.13, 3.22) | 0.709 | -0.16(-0.25, -0.06) | 0.990 | -0.04(-0.11, 0.03) |
| Zinc, mg | 18.70(16.95, 20.45) | | 18.48(17.28, 19.68) | 0.028 | -0.67(-2.70, 1.36) | 0.002 | -2.61(-4.65, -0.57) |
| Copper, mg | 2.06(1.90, 2.21) | | 1.64(1.56, 1.71) | < 0.001 | -0.52(-0.72, -0.32) | < 0.001 | -0.70(-0.84, -0.57) |
| Selenium, mcg | 122.27(116.83, 127.71) | | 124.79(119.55, 130.04) | 0.946 | -1.61(-9.28, 6.07) | 0.537 | -7.97(-17.26, 1.31) |
| **FB ^e^** | | |  |  |  |  |  |
| Calcium, g | 0.84(0.81, 0.87) | | 0.89(0.87, 0.92) | < 0.001 | 0.06(0.02, 0.10) | 0.929 | 0.02(-0.01, 0.05) |
| Iron, mg | 16.57(15.86, 17.28) | | 14.03(13.74, 14.32) | < 0.001 | -2.62(-3.79, -1.46) | < 0.001 | -2.43(-3.00, -1.87) |
| Magnesium, mg | 300.63(292.75, 308.52) | | 299.35(292.72, 305.97) | 0.328 | -7.58(-17.26, 2.10) | 0.563 | 10.30(0.58, 20.02) |
| Phosphorus, g | 1.29(1.27, 1.31) | | 1.31(1.30, 1.33) | 0.001 | 0.01(-0.01, 0.03) | 0.178 | 0.02(-0.01, 0.04) |
| Potassium, g | 2.94(2.86, 3.01) | | 2.68(2.62, 2.73) | < 0.001 | -0.30(-0.40, -0.19) | < 0.001 | -0.16(-0.25, -0.07) |
| Sodium, g | 3.31(3.23, 3.38) | | 3.17(3.13, 3.21) | 0.603 | -0.16(-0.25, -0.06) | 0.929 | -0.04(-0.11, 0.04) |
| Zinc, mg | 11.55(11.01, 12.10) | | 10.27(10.10, 10.44) | < 0.001 | -0.93(-1.35, -0.51) | < 0.001 | -2.06(-3.29, -0.83) |
| Copper, mg | 1.31(1.24, 1.37) | | 1.24(1.18, 1.30) | 0.001 | -0.10(-0.20, 0.00) | < 0.001 | -0.12(-0.24, -0.01) |
| Selenium, mcg | 104.85(101.54, 108.16) | | 105.45(103.58, 107.31) | 0.082 | -2.12(-6.32, 2.09) | 0.219 | -0.80(-4.78, 3.17) |
| **Race/ethnicity** |  | |  |  |  |  |  |
| **Hispanic ^f^** |  | |  |  |  |  |  |
| **FB ^e^ + DSs** | | |  |  |  |  |  |
| Calcium, g | - | | 1.00(0.97, 1.03) | 0.534 | -0.01(-0.06, 0.04) | - | - |
| Iron, mg | - | | 15.63(14.91, 16.36) | 0.007 | -1.51(-2.51, -0.51) | - | - |
| Magnesium, mg | - | | 318.44(306.08, 330.80) | 0.205 | 10.74(-6.37, 27.84) | - | - |
| Phosphorus, g | - | | 1.34(1.32, 1.37) | 0.310 | 0.05(0.00, 0.09) | - | - |
| Potassium, g | - | | 2.51(2.45, 2.57) | 0.107 | -0.04(-0.14, 0.06) | - | - |
| Sodium, g | - | | 3.30(3.22, 3.38) | 0.107 | 0.14(0.05, 0.23) | - | - |
| Zinc, mg | - | | 13.28(12.32, 14.25) | 0.113 | -1.06(-2.05, -0.06) | - | - |
| Copper, mg | - | | 1.29(1.24, 1.35) | < 0.001 | -0.21(-0.30, -0.12) | - | - |
| Selenium, mcg | - | | 122.05(118.42, 125.68) | 0.381 | 5.78(1.67, 9.90) | - | - |
| **FB ^e^** | | |  |  |  |  |  |
| Calcium, g | - | | 0.91(0.88, 0.94) | 0.937 | 0.00(-0.04, 0.04) | - | - |
| Iron, mg | - | | 13.49(13.16, 13.81) | < 0.001 | -1.18(-1.66, -0.69) | - | - |
| Magnesium, mg | - | | 298.89(288.64, 309.13) | 0.142 | 10.98(-2.54, 24.49) | - | - |
| Phosphorus, g | - | | 1.34(1.31, 1.36) | 0.180 | 0.05(0.01, 0.10) | - | - |
| Potassium, g | - | | 2.50(2.44, 2.56) | 0.073 | -0.04(-0.14, 0.06) | - | - |
| Sodium, g | - | | 3.30(3.21, 3.38) | 0.073 | 0.14(0.05, 0.23) | - | - |
| Zinc, mg | - | | 10.74(10.42, 11.06) | 0.029 | -0.96(-1.71, -0.21) | - | - |
| Copper, mg | - | | 1.15(1.10, 1.20) | 0.005 | -0.10(-0.16, -0.03) | - | - |
| Selenium, mcg | - | | 114.74(111.48, 118.00) | 0.027 | 5.85(2.14, 9.56) | - | - |
| **Non-Hispanic White** |  | |  |  |  |  |  |
| **FB ^e^ + DSs** | | |  |  |  |  |  |
| Calcium, g | 1.01(0.95, 1.06) | | 1.06(1.03, 1.10) | 0.052 | 0.04(-0.02, 0.10) | 0.002 | -0.05(-0.11, 0.00) |
| Iron, mg | 19.98(18.85, 21.11) | | 16.61(15.83, 17.39) | < 0.001 | -3.87(-5.34, -2.40) | < 0.001 | -3.47(-4.68, -2.27) |
| Magnesium, mg | 321.42(303.03, 339.80) | | 331.70(321.19, 342.21) | 0.115 | -3.67(-20.49, 13.15) | 0.716 | 11.76(-0.54, 24.05) |
| Phosphorus, g | 1.27(1.24, 1.31) | | 1.31(1.29, 1.33) | < 0.001 | 0.03(0.00, 0.06) | 0.007 | 0.03(0.00, 0.06) |
| Potassium, g | 2.72(2.61, 2.83) | | 2.53(2.47, 2.59) | < 0.001 | -0.26(-0.35, -0.16) | < 0.001 | -0.15(-0.23, -0.07) |
| Sodium, g | 3.26(3.17, 3.35) | | 3.24(3.19, 3.29) | 0.029 | -0.03(-0.13, 0.08) | 0.985 | -0.03(-0.10, 0.04) |
| Zinc, mg | 17.41(16.44, 18.37) | | 14.96(14.26, 15.65) | < 0.001 | -3.27(-4.52, -2.01) | < 0.001 | -3.67(-4.78, -2.56) |
| Copper, mg | 1.89(1.78, 2.00) | | 1.41(1.35, 1.46) | < 0.001 | -0.57(-0.69, -0.45) | < 0.001 | -0.52(-0.61, -0.43) |
| Selenium, mcg | 119.74(116.31, 123.17) | | 120.27(118.28, 122.26) | 0.921 | -1.81(-5.99, 2.36) | 0.190 | -4.93(-9.07, -0.80) |
| **FB ^e^** | | |  |  |  |  |  |
| Calcium, g | 0.81(0.77, 0.86) | | 0.91(0.89, 0.94) | < 0.001 | 0.11(0.06, 0.15) | 0.001 | 0.07(0.03, 0.10) |
| Iron, mg | 14.79(14.08, 15.50) | | 13.34(13.04, 13.64) | < 0.001 | -1.69(-2.42, -0.95) | < 0.001 | -2.00(-2.53, -1.47) |
| Magnesium, mg | 278.17(264.59, 291.75) | | 291.75(285.41, 298.08) | < 0.001 | 6.45(-5.39, 18.29) | 0.001 | 20.95(13.04, 28.87) |
| Phosphorus, g | 1.24(1.21, 1.28) | | 1.31(1.29, 1.33) | < 0.001 | 0.05(0.02, 0.09) | < 0.001 | 0.04(0.01, 0.08) |
| Potassium, g | 2.70(2.59, 2.81) | | 2.52(2.46, 2.57) | < 0.001 | -0.25(-0.34, -0.16) | < 0.001 | -0.14(-0.21, -0.06) |
| Sodium, g | 3.26(3.17, 3.35) | | 3.24(3.19, 3.28) | 0.018 | -0.03(-0.13, 0.08) | 0.953 | -0.03(-0.10, 0.04) |
| Zinc, mg | 11.03(10.73, 11.33) | | 10.30(10.15, 10.44) | < 0.001 | -0.78(-1.15, -0.41) | < 0.001 | -1.37(-1.95, -0.79) |
| Copper, mg | 1.23(1.18, 1.29) | | 1.15(1.10, 1.19) | < 0.001 | -0.11(-0.17, -0.06) | < 0.001 | -0.07(-0.13, -0.01) |
| Selenium, mcg | 101.88(99.15, 104.60) | | 105.62(104.26, 106.97) | < 0.001 | 3.56(0.83, 6.28) | < 0.001 | 2.93(0.42, 5.44) |
| **Non-Hispanic Black** |  | |  |  |  |  |  |
| **FB ^e^ + DSs** | | |  |  |  |  |  |
| Calcium, g | 0.67(0.63, 0.72) | | 0.87(0.84, 0.89) | < 0.001 | 0.19(0.14, 0.24) | 0.086 | 0.11(0.05, 0.16) |
| Iron, mg | 16.80(16.00, 17.61) | | 16.27(14.98, 17.55) | 0.392 | -0.83(-2.10, 0.44) | 0.633 | 0.11(-1.41, 1.63) |
| Magnesium, mg | 245.46(233.47, 257.46) | | 268.04(257.32, 278.75) | 0.002 | 13.62(-3.39, 30.62) | 0.086 | 19.13(-1.53, 39.80) |
| Phosphorus, g | 1.09(1.04, 1.14) | | 1.20(1.18, 1.22) | < 0.001 | 0.11(0.06, 0.15) | < 0.001 | 0.09(0.04, 0.14) |
| Potassium, g | 2.28(2.16, 2.41) | | 2.22(2.17, 2.28) | 0.930 | -0.11(-0.25, 0.02) | 0.560 | 0.02(-0.05, 0.08) |
| Sodium, g | 3.13(3.03, 3.24) | | 3.19(3.15, 3.23) | < 0.001 | 0.06(-0.07, 0.18) | 0.049 | 0.06(-0.06, 0.18) |
| Zinc, mg | 13.97(12.90, 15.03) | | 11.63(11.19, 12.08) | < 0.001 | -1.93(-3.05, -0.81) | < 0.001 | -2.25(-3.18, -1.33) |
| Copper, mg | 1.45(1.36, 1.54) | | 1.22(1.16, 1.29) | < 0.001 | -0.28(-0.42, -0.14) | < 0.001 | -0.14(-0.27, -0.02) |
| Selenium, mcg | 114.81(104.74, 124.87) | | 114.35(110.78, 117.91) | 0.075 | -0.74(-12.12, 10.64) | 0.099 | 0.02(-7.60, 7.64) |
| **FB ^e^** | | |  |  |  |  |  |
| Calcium, g | 0.61(0.58, 0.65) | | 0.79(0.78, 0.81) | < 0.001 | 0.19(0.16, 0.22) | < 0.001 | 0.14(0.09, 0.18) |
| Iron, mg | 13.82(13.22, 14.41) | | 12.51(12.14, 12.89) | < 0.001 | -1.16(-2.01, -0.32) | < 0.001 | -1.08(-1.81, -0.36) |
| Magnesium, mg | 228.39(216.13, 240.65) | | 253.04(245.43, 260.64) | < 0.001 | 20.55(5.39, 35.71) | < 0.001 | 29.59(19.16, 40.03) |
| Phosphorus, g | 1.08(1.03, 1.13) | | 1.20(1.18, 1.22) | < 0.001 | 0.11(0.07, 0.16) | < 0.001 | 0.09(0.04, 0.14) |
| Potassium, g | 2.27(2.15, 2.40) | | 2.21(2.16, 2.27) | 0.973 | -0.11(-0.24, 0.03) | 0.408 | 0.02(-0.05, 0.09) |
| Sodium, g | 3.13(3.02, 3.24) | | 3.19(3.15, 3.23) | < 0.001 | 0.06(-0.07, 0.18) | 0.025 | 0.06(-0.06, 0.18) |
| Zinc, mg | 10.85(9.80, 11.90) | | 9.24(9.05, 9.43) | < 0.001 | -0.89(-1.52, -0.26) | < 0.001 | -1.66(-2.60, -0.71) |
| Copper, mg | 1.16(1.08, 1.24) | | 1.05(0.99, 1.10) | 0.011 | -0.10(-0.21, 0.01) | 0.007 | -0.01(-0.10, 0.07) |
| Selenium, mcg | 107.13(97.68, 116.57) | | 105.71(103.48, 107.94) | 0.003 | -0.38(-10, 9.25) | < 0.001 | 3.31(-1.17, 7.79) |
| **Education level** |  | |  |  |  |  |  |
| **Less than high school graduate** | | |  |  |  |  |  |
| **FB ^e^ + DSs** | | |  |  |  |  |  |
| Calcium, g | 0.85(0.79, 0.90) | | 0.97(0.93, 1.00) | < 0.001 | 0.12(0.05, 0.19) | 0.016 | 0.07(0.00, 0.13) |
| Iron, mg | 17.11(16.46, 17.77) | | 15.99(15.35, 16.64) | 0.031 | -1.51(-2.59, -0.43) | 0.040 | -0.93(-2.13, 0.27) |
| Magnesium, mg | 284.78(274.16, 295.40) | | 296.22(282.09, 310.36) | 0.011 | 6.30(-13.04, 25.65) | 0.092 | 21.99(4.18, 39.79) |
| Phosphorus, g | 1.21(1.18, 1.25) | | 1.29(1.26, 1.32) | < 0.001 | 0.06(0.02, 0.11) | 0.006 | 0.05(0.01, 0.09) |
| Potassium, g | 2.57(2.49, 2.65) | | 2.45(2.36, 2.53) | 0.011 | -0.14(-0.29, 0.00) | 0.006 | -0.05(-0.16, 0.05) |
| Sodium, g | 3.15(3.03, 3.26) | | 3.14(3.07, 3.21) | 0.001 | -0.01(-0.15, 0.13) | 0.007 | 0.03(-0.10, 0.16) |
| Zinc, mg | 14.75(13.72, 15.79) | | 12.37(11.57, 13.16) | < 0.001 | -1.85(-3.15, -0.56) | < 0.001 | -1.65(-2.86, -0.44) |
| Copper, mg | 1.58(1.49, 1.67) | | 1.22(1.17, 1.27) | < 0.001 | -0.34(-0.46, -0.22) | < 0.001 | -0.19(-0.28, -0.10) |
| Selenium, mcg | 113.42(109.60, 117.23) | | 112.77(109.27, 116.27) | 0.024 | -0.03(-6.05, 5.98) | 0.016 | 2.61(-3.64, 8.86) |
| **FB ^e^** | | |  |  |  |  |  |
| Calcium, g | 0.73(0.70, 0.76) | | 0.88(0.84, 0.92) | < 0.001 | 0.15(0.09, 0.21) | < 0.001 | 0.11(0.04, 0.18) |
| Iron, mg | 14.13(13.44, 14.82) | | 13.14(12.67, 13.62) | 0.018 | -1.08(-1.93, -0.23) | < 0.001 | -1.56(-2.44, -0.69) |
| Magnesium, mg | 260.56(253.22, 267.90) | | 279.94(267.93, 291.94) | < 0.001 | 14.57(-0.68, 29.81) | 0.010 | 24.10(9.31, 38.90) |
| Phosphorus, g | 1.20(1.17, 1.23) | | 1.29(1.26, 1.31) | < 0.001 | 0.07(0.03, 0.12) | 0.001 | 0.06(0.01, 0.10) |
| Potassium, g | 2.56(2.47, 2.64) | | 2.44(2.36, 2.52) | 0.014 | -0.13(-0.28, 0.01) | 0.005 | -0.05(-0.16, 0.05) |
| Sodium, g | 3.14(3.03, 3.26) | | 3.14(3.07, 3.21) | 0.001 | -0.01(-0.15, 0.13) | 0.006 | 0.03(-0.10, 0.16) |
| Zinc, mg | 11.07(10.40, 11.73) | | 10.05(9.68, 10.41) | < 0.001 | -0.73(-1.39, -0.08) | < 0.001 | -1.09(-1.79, -0.40) |
| Copper, mg | 1.17(1.12, 1.23) | | 1.07(1.01, 1.12) | < 0.001 | -0.09(-0.17, -0.02) | < 0.001 | -0.03(-0.10, 0.04) |
| Selenium, mcg | 102.20(97.89, 106.52) | | 106.19(102.25, 110.14) | 0.001 | 4.37(-1.95, 10.69) | 0.010 | 2.54(-3.67, 8.75) |
| **High school or equivalent** | | |  |  |  |  |  |
| **FB ^e^ + DSs** | | |  |  |  |  |  |
| Calcium, g | 0.89(0.84, 0.94) | | 1.00(0.96, 1.04) | < 0.001 | 0.13(0.06, 0.20) | 0.832 | 0.01(-0.06, 0.08) |
| Iron, mg | 17.73(16.85, 18.62) | | 15.75(15.10, 16.39) | < 0.001 | -2.05(-3.39, -0.72) | < 0.001 | -2.34(-3.56, -1.13) |
| Magnesium, mg | 285.71(272.97, 298.45) | | 290.28(279.81, 300.74) | 0.268 | 1.95(-13.25, 17.16) | 0.398 | 1.25(-12.97, 15.46) |
| Phosphorus, g | 1.20(1.16, 1.23) | | 1.27(1.24, 1.29) | < 0.001 | 0.08(0.03, 0.13) | 0.006 | 0.04(-0.01, 0.08) |
| Potassium, g | 2.63(2.51, 2.75) | | 2.35(2.29, 2.42) | < 0.001 | -0.30(-0.44, -0.16) | < 0.001 | -0.19(-0.28, -0.10) |
| Sodium, g | 3.27(3.13, 3.40) | | 3.24(3.16, 3.31) | 0.038 | 0.00(-0.16, 0.17) | 0.517 | 0.03(-0.08, 0.14) |
| Zinc, mg | 15.21(14.39, 16.04) | | 13.76(12.95, 14.57) | < 0.001 | -1.45(-2.76, -0.15) | < 0.001 | -3.03(-4.07, -1.99) |
| Copper, mg | 1.61(1.53, 1.70) | | 1.24(1.18, 1.29) | < 0.001 | -0.41(-0.53, -0.30) | < 0.001 | -0.47(-0.57, -0.37) |
| Selenium, mcg | 111.93(107.55, 116.30) | | 116.93(113.45, 120.42) | 0.078 | 3.73(-3.11, 10.57) | 0.832 | -1.42(-7.01, 4.16) |
| **FB ^e^** | | |  |  |  |  |  |
| Calcium, g | 0.75(0.71, 0.78) | | 0.88(0.85, 0.91) | < 0.001 | 0.15(0.09, 0.20) | < 0.001 | 0.09(0.03, 0.15) |
| Iron, mg | 14.10(13.55, 14.65) | | 13.30(12.86, 13.74) | < 0.001 | -0.83(-1.69, 0.03) | < 0.001 | -1.28(-2.06, -0.51) |
| Magnesium, mg | 259.00(250.61, 267.39) | | 265.26(259.13, 271.39) | < 0.001 | 3.64(-7.00, 14.27) | 0.047 | 13.89(4.27, 23.51) |
| Phosphorus, g | 1.18(1.14, 1.21) | | 1.26(1.24, 1.28) | < 0.001 | 0.09(0.05, 0.14) | < 0.001 | 0.06(0.01, 0.10) |
| Potassium, g | 2.61(2.49, 2.73) | | 2.34(2.28, 2.41) | < 0.001 | -0.30(-0.43, -0.16) | < 0.001 | -0.18(-0.27, -0.09) |
| Sodium, g | 3.27(3.13, 3.40) | | 3.24(3.16, 3.31) | 0.027 | 0(-0.16, 0.17) | 0.389 | 0.03(-0.08, 0.14) |
| Zinc, mg | 10.75(10.34, 11.15) | | 10.24(9.93, 10.55) | < 0.001 | -0.45(-1.14, 0.23) | < 0.001 | -1.17(-1.75, -0.59) |
| Copper, mg | 1.17(1.14, 1.21) | | 1.04(1.01, 1.08) | < 0.001 | -0.15(-0.21, -0.09) | < 0.001 | -0.09(-0.15, -0.03) |
| Selenium, mcg | 101.20(97.70, 104.70) | | 108.88(105.84, 111.91) | < 0.001 | 7.66(2.21, 13.11) | < 0.001 | 9.56(5.02, 14.10) |
| **College or above** | | |  |  |  |  |  |
| **FB ^e^ + DSs** | | |  |  |  |  |  |
| Calcium, g | 1.00(0.94, 1.06) | | 1.04(1.01, 1.07) | 0.126 | 0.03(-0.03, 0.10) | 0.001 | -0.04(-0.09, 0.02) |
| Iron, mg | 20.93(19.66, 22.19) | | 16.72(15.98, 17.46) | < 0.001 | -4.43(-6.00, -2.85) | < 0.001 | -3.60(-5.12, -2.09) |
| Magnesium, mg | 331.10(316.52, 345.67) | | 339.93(330.02, 349.84) | 0.005 | 1.17(-16.43, 18.78) | 0.110 | 20.09(6.13, 34.05) |
| Phosphorus, g | 1.27(1.24, 1.31) | | 1.32(1.31, 1.34) | < 0.001 | 0.04(0.00, 0.08) | < 0.001 | 0.04(0.01, 0.08) |
| Potassium, g | 2.69(2.61, 2.77) | | 2.57(2.52, 2.62) | < 0.001 | -0.18(-0.27, -0.09) | 0.001 | -0.09(-0.17, 0.00) |
| Sodium, g | 3.27(3.18, 3.35) | | 3.29(3.25, 3.33) | 0.001 | 0.03(-0.08, 0.14) | 0.513 | -0.01(-0.08, 0.07) |
| Zinc, mg | 17.91(16.68, 19.14) | | 14.68(14.14, 15.21) | < 0.001 | -3.72(-5.07, -2.36) | < 0.001 | -3.34(-4.48, -2.20) |
| Copper, mg | 1.98(1.89, 2.08) | | 1.47(1.42, 1.51) | < 0.001 | -0.57(-0.68, -0.47) | < 0.001 | -0.45(-0.55, -0.36) |
| Selenium, mcg | 122.90(118.11, 127.68) | | 123.02(120.78, 125.27) | 0.298 | -1.98(-7.30, 3.34) | 0.499 | -3.41(-8.60, 1.77) |
| **FB ^e^** | | |  |  |  |  |  |
| Calcium, g | 0.81(0.76, 0.86) | | 0.90(0.88, 0.93) | < 0.001 | 0.10(0.05, 0.16) | 0.004 | 0.08(0.04, 0.12) |
| Iron, mg | 14.88(14.33, 15.43) | | 13.33(13.06, 13.60) | < 0.001 | -1.65(-2.32, -0.98) | < 0.001 | -1.99(-2.58, -1.40) |
| Magnesium, mg | 282.23(270.78, 293.69) | | 302.33(296.26, 308.40) | < 0.001 | 15.90(2.65, 29.15) | < 0.001 | 28.14(18.19, 38.10) |
| Phosphorus, g | 1.24(1.21, 1.28) | | 1.32(1.30, 1.33) | < 0.001 | 0.06(0.02, 0.11) | < 0.001 | 0.06(0.02, 0.09) |
| Potassium, g | 2.67(2.59, 2.75) | | 2.56(2.51, 2.61) | 0.001 | -0.17(-0.26, -0.08) | 0.002 | -0.08(-0.16, 0.00) |
| Sodium, g | 3.27(3.18, 3.35) | | 3.29(3.25, 3.33) | 0.001 | 0.03(-0.08, 0.14) | 0.493 | -0.01(-0.08, 0.07) |
| Zinc, mg | 10.97(10.58, 11.36) | | 10.26(10.14, 10.37) | < 0.001 | -0.82(-1.25, -0.39) | < 0.001 | -1.30(-1.85, -0.74) |
| Copper, mg | 1.28(1.24, 1.31) | | 1.21(1.18, 1.25) | 0.004 | -0.08(-0.14, -0.03) | < 0.001 | -0.05(-0.10, 0.01) |
| Selenium, mcg | 103.23(101.26, 105.19) | | 107.70(106.22, 109.17) | < 0.001 | 3.10(0.36, 5.84) | 0.001 | 1.61(-1.06, 4.29) |
| **Ratio of family income to poverty level** | | |  |  |  |  |  |
| **< 1.30** |  | |  |  |  |  |  |
| **FB ^e^ + DSs** | | |  |  |  |  |  |
| Calcium, g | 0.83(0.77, 0.89) | | 0.95(0.93, 0.98) | < 0.001 | 0.13(0.08, 0.18) | 0.005 | 0.06(0.02, 0.10) |
| Iron, mg | 16.09(14.85, 17.34) | | 15.38(14.67, 16.08) | 0.077 | -0.55(-2.08, 0.99) | 0.005 | -1.76(-3.22, -0.31) |
| Magnesium, mg | 273.27(259.58, 286.95) | | 281.53(272.97, 290.09) | 0.003 | 10.71(-3.83, 25.24) | 0.403 | 13.69(-2.22, 29.60) |
| Phosphorus, g | 1.19(1.16, 1.22) | | 1.25(1.24, 1.27) | < 0.001 | 0.06(0.03, 0.09) | < 0.001 | 0.07(0.04, 0.10) |
| Potassium, g | 2.46(2.36, 2.57) | | 2.36(2.30, 2.42) | 0.077 | -0.11(-0.22, 0.00) | 0.006 | -0.07(-0.17, 0.03) |
| Sodium, g | 3.11(2.96, 3.26) | | 3.20(3.14, 3.25) | < 0.001 | 0.03(-0.14, 0.19) | 0.001 | 0.02(-0.10, 0.14) |
| Zinc, mg | 13.62(12.28, 14.96) | | 11.82(11.35, 12.28) | < 0.001 | -1.72(-3.13, -0.32) | < 0.001 | -2.07(-2.97, -1.17) |
| Copper, mg | 1.53(1.40, 1.66) | | 1.18(1.13, 1.24) | < 0.001 | -0.35(-0.48, -0.22) | < 0.001 | -0.28(-0.39, -0.16) |
| Selenium, mcg | 110.32(105.27, 115.37) | | 112.29(109.94, 114.65) | < 0.001 | 1.32(-5.26, 7.91) | 0.009 | 4.28(-3.38, 11.93) |
| **FB ^e^** | | |  |  |  |  |  |
| Calcium, g | 0.72(0.69, 0.76) | | 0.87(0.85, 0.89) | < 0.001 | 0.16(0.11, 0.20) | < 0.001 | 0.12(0.08, 0.15) |
| Iron, mg | 13.51(13.03, 13.98) | | 13.12(12.70, 13.53) | 0.016 | -0.31(-1.02, 0.40) | < 0.001 | -1.16(-1.76, -0.55) |
| Magnesium, mg | 251.23(242.24, 260.22) | | 266.61(259.39, 273.82) | < 0.001 | 16.62(4.92, 28.32) | 0.032 | 20.53(8.38, 32.68) |
| Phosphorus, g | 1.18(1.15, 1.20) | | 1.25(1.24, 1.27) | < 0.001 | 0.08(0.05, 0.11) | < 0.001 | 0.08(0.05, 0.11) |
| Potassium, g | 2.45(2.35, 2.55) | | 2.35(2.29, 2.41) | 0.099 | -0.10(-0.21, 0.01) | 0.007 | -0.07(-0.17, 0.03) |
| Sodium, g | 3.11(2.96, 3.26) | | 3.19(3.14, 3.25) | < 0.001 | 0.03(-0.14, 0.19) | < 0.001 | 0.02(-0.10, 0.14) |
| Zinc, mg | 10.26(9.66, 10.85) | | 9.70(9.51, 9.89) | 0.002 | -0.60(-1.28, 0.08) | < 0.001 | -0.87(-1.36, -0.37) |
| Copper, mg | 1.16(1.10, 1.22) | | 1.04(0.99, 1.10) | < 0.001 | -0.12(-0.22, -0.02) | < 0.001 | -0.09(-0.17, 0.00) |
| Selenium, mcg | 98.35(93.92, 102.78) | | 107.50(104.95, 110.04) | < 0.001 | 8.00(2.26, 13.74) | < 0.001 | 7.49(2.09, 12.89) |
| **1.30-3.49** |  | |  |  |  |  |  |
| **FB ^e^ + DSs** | | |  |  |  |  |  |
| Calcium, g | 0.91(0.84, 0.99) | | 1.02(0.99, 1.05) | < 0.001 | 0.08(-0.01, 0.17) | 0.248 | -0.01(-0.05, 0.04) |
| Iron, mg | 18.95(18.23, 19.66) | | 17.11(15.94, 18.27) | 0.077 | -2.50(-3.95, -1.06) | < 0.001 | -2.05(-3.78, -0.31) |
| Magnesium, mg | 293.42(283.42, 303.41) | | 316.27(301.71, 330.83) | 0.003 | 12.31(-4.40, 29.01) | 0.223 | 20.07(2.11, 38.03) |
| Phosphorus, g | 1.22(1.19, 1.26) | | 1.28(1.27, 1.30) | < 0.001 | 0.05(0.01, 0.09) | 0.006 | 0.03(-0.01, 0.06) |
| Potassium, g | 2.60(2.49, 2.71) | | 2.43(2.38, 2.49) | 0.077 | -0.23(-0.34, -0.11) | < 0.001 | -0.14(-0.21, -0.06) |
| Sodium, g | 3.26(3.14, 3.38) | | 3.25(3.19, 3.31) | < 0.001 | -0.04(-0.18, 0.10) | 0.439 | 0.03(-0.06, 0.12) |
| Zinc, mg | 15.32(14.13, 16.51) | | 14.33(13.54, 15.12) | < 0.001 | -1.60(-3.07, -0.13) | < 0.001 | -2.90(-4.04, -1.76) |
| Copper, mg | 1.66(1.55, 1.77) | | 1.36(1.29, 1.43) | < 0.001 | -0.38(-0.52, -0.25) | < 0.001 | -0.41(-0.54, -0.29) |
| Selenium, mcg | 113.96(109.23, 118.68) | | 118.97(115.49, 122.45) | < 0.001 | 2.54(-3.82, 8.91) | 0.790 | -1.01(-7.21, 5.19) |
| **FB ^e^** | | |  |  |  |  |  |
| Calcium, g | 0.77(0.71, 0.83) | | 0.89(0.87, 0.91) | < 0.001 | 0.11(0.04, 0.18) | < 0.001 | 0.08(0.04, 0.12) |
| Iron, mg | 14.49(13.89, 15.08) | | 13.52(13.18, 13.87) | 0.016 | -1.27(-1.99, -0.54) | < 0.001 | -1.54(-2.10, -0.98) |
| Magnesium, mg | 264.67(255.16, 274.19) | | 279.42(271.83, 287.01) | < 0.001 | 7.71(-4.86, 20.28) | 0.003 | 20.96(10.42, 31.49) |
| Phosphorus, g | 1.20(1.17, 1.24) | | 1.28(1.26, 1.29) | < 0.001 | 0.07(0.03, 0.10) | < 0.001 | 0.04(0.01, 0.08) |
| Potassium, g | 2.58(2.47, 2.69) | | 2.42(2.37, 2.47) | 0.099 | -0.22(-0.34, -0.11) | < 0.001 | -0.13(-0.20, -0.05) |
| Sodium, g | 3.26(3.14, 3.38) | | 3.24(3.18, 3.31) | < 0.001 | -0.04(-0.18, 0.10) | 0.359 | 0.03(-0.06, 0.12) |
| Zinc, mg | 10.55(10.22, 10.88) | | 10.19(10.01, 10.38) | 0.002 | -0.45(-0.88, -0.02) | < 0.001 | -1.25(-1.68, -0.82) |
| Copper, mg | 1.18(1.14, 1.23) | | 1.12(1.06, 1.18) | < 0.001 | -0.12(-0.19, -0.04) | < 0.001 | -0.07(-0.15, 0.01) |
| Selenium, mcg | 100.03(95.89, 104.17) | | 106.14(103.97, 108.31) | < 0.001 | 4.19(-0.60, 8.98) | 0.016 | 2.88(-1.63, 7.40) |
| **≥3.50** |  | |  |  |  |  |  |
| **FB ^e^ + DSs** | | |  |  |  |  |  |
| Calcium, g | 1.01(0.95, 1.07) | | 1.06(1.02, 1.10) | 0.324 | 0.03(-0.04, 0.11) | 0.007 | -0.04(-0.12, 0.03) |
| Iron, mg | 20.98(19.64, 22.31) | | 16.11(15.40, 16.83) | < 0.001 | -5.21(-6.82, -3.59) | < 0.001 | -4.14(-5.38, -2.91) |
| Magnesium, mg | 339.73(321.02, 358.45) | | 342.44(328.20, 356.68) | 0.319 | -9.39(-31.41, 12.63) | 0.775 | 11.40(-6.05, 28.86) |
| Phosphorus, g | 1.28(1.25, 1.31) | | 1.34(1.32, 1.36) | < 0.001 | 0.05(0.01, 0.09) | 0.007 | 0.04(-0.01, 0.09) |
| Potassium, g | 2.78(2.68, 2.89) | | 2.61(2.54, 2.68) | < 0.001 | -0.24(-0.35, -0.13) | 0.007 | -0.11(-0.21, -0.01) |
| Sodium, g | 3.27(3.20, 3.34) | | 3.30(3.24, 3.36) | 0.004 | 0.04(-0.05, 0.14) | 0.851 | -0.01(-0.10, 0.07) |
| Zinc, mg | 18.58(17.37, 19.79) | | 15.15(14.46, 15.84) | < 0.001 | -4.31(-5.68, -2.94) | < 0.001 | -3.66(-4.86, -2.46) |
| Copper, mg | 2.03(1.90, 2.17) | | 1.48(1.43, 1.53) | < 0.001 | -0.63(-0.78, -0.49) | < 0.001 | -0.50(-0.60, -0.41) |
| Selenium, mcg | 125.50(120.41, 130.59) | | 124.78(122.72, 126.84) | 0.960 | -3.06(-8.61, 2.48) | 0.218 | -5.92(-12.73, 0.89) |
| **FB ^e^** | | |  |  |  |  |  |
| Calcium, g | 0.81(0.77, 0.85) | | 0.92(0.89, 0.95) | < 0.001 | 0.11(0.07, 0.16) | 0.022 | 0.08(0.02, 0.13) |
| Iron, mg | 15.00(14.25, 15.75) | | 13.27(12.97, 13.58) | < 0.001 | -1.90(-2.73, -1.07) | < 0.001 | -2.19(-2.93, -1.46) |
| Magnesium, mg | 286.41(273.76, 299.05) | | 306.76(299.05, 314.48) | < 0.001 | 13.99(-0.53, 28.51) | < 0.001 | 27.28(15.89, 38.68) |
| Phosphorus, g | 1.25(1.22, 1.28) | | 1.33(1.31, 1.35) | < 0.001 | 0.08(0.04, 0.11) | < 0.001 | 0.06(0.01, 0.11) |
| Potassium, g | 2.76(2.65, 2.86) | | 2.60(2.53, 2.67) | 0.001 | -0.23(-0.34, -0.11) | 0.015 | -0.10(-0.20, 0.00) |
| Sodium, g | 3.26(3.20, 3.33) | | 3.30(3.24, 3.35) | 0.003 | 0.04(-0.05, 0.14) | 0.850 | -0.01(-0.10, 0.07) |
| Zinc, mg | 11.42(11.02, 11.83) | | 10.45(10.28, 10.63) | < 0.001 | -0.95(-1.43, -0.47) | < 0.001 | -1.38(-2.11, -0.64) |
| Copper, mg | 1.28(1.23, 1.33) | | 1.23(1.19, 1.26) | 0.001 | -0.09(-0.14, -0.03) | < 0.001 | -0.03(-0.09, 0.04) |
| Selenium, mcg | 106.20(103.53, 108.88) | | 109.40(107.77, 111.02) | < 0.001 | 2.73(-0.35, 5.80) | 0.004 | 3.20(-0.14, 6.55) |

Abbreviations: NHANES, National Health and Nutrition Examination Survey; FB, foods and beverages; DSs, Dietary supplements.

a Data were adjusted for NHANES survey weights.

b Results were adjusted for gender, age, race/ethnicity, education level, and family income-to-poverty ratio.

c 1999-2000 vs. 2017-2020, 2007-2008 vs. 2017-2020 for Hispanic, and differences were adjusted for gender, age, race/ethnicity, education level, and family income when appropriate.

d 2003-2004 vs. 2017-2020, and differences were adjusted for gender, age, race/ethnicity, education level, and family income when appropriate.

e Data were adjusted for total energy intake to 2000 kcal/d using the residual method.

f The Hispanic group was defined starting in 2007, so the Hispanic ethnic group was only analytically assessed from 2007 onward.

**Supplementary Figure 1.** Participant flow chart.


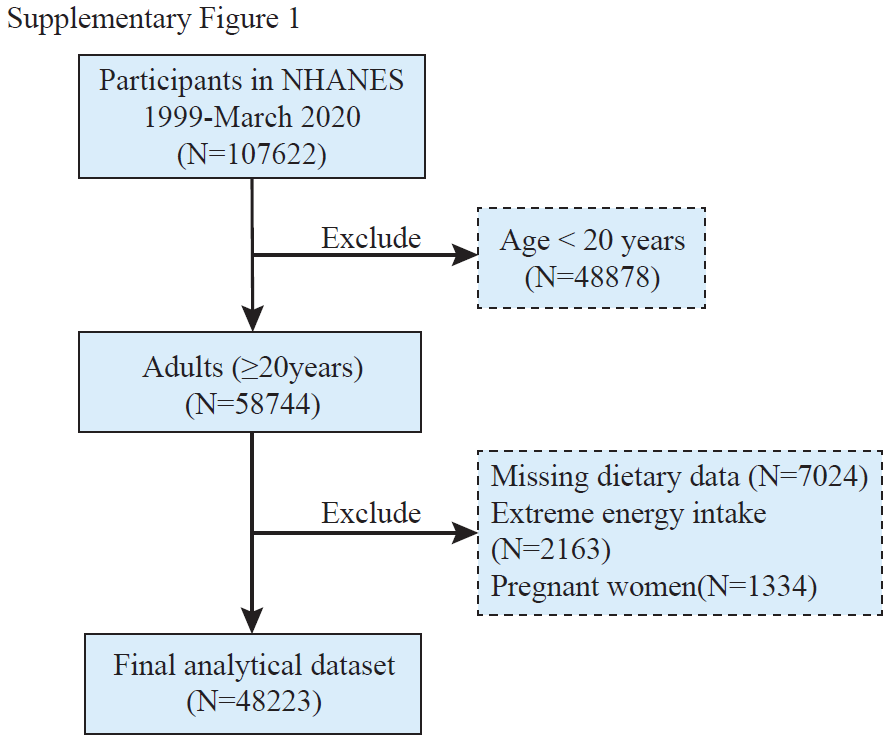


**Supplementary Figure 2. Trends in Age-adjusted Percentage of U.S. Adults Meeting RDAs/AIs by NHANES Survey Cycle, 1999-2020.**

Age-adjusted percentage was determined by direct standardization using civilian noninstitutionalized population in 2017-2020 NHANES cycles as a reference (aged 20-39 years, 40-59 years and ≥60 years). Analyses are based on energy-adjusted values to 2000 kcal/d using the residual method. All estimates were weighted, and error bars indicated 95% CIs. Results were adjusted for gender, age, race/ethnicity, education level, and family income. Abbreviations: AIs, Adequate Intakes; DSs, Dietary supplements; FB, foods and beverages; NHANES, National Health and Nutrition Examination Survey; RDAs, Recommended Dietary Allowances.


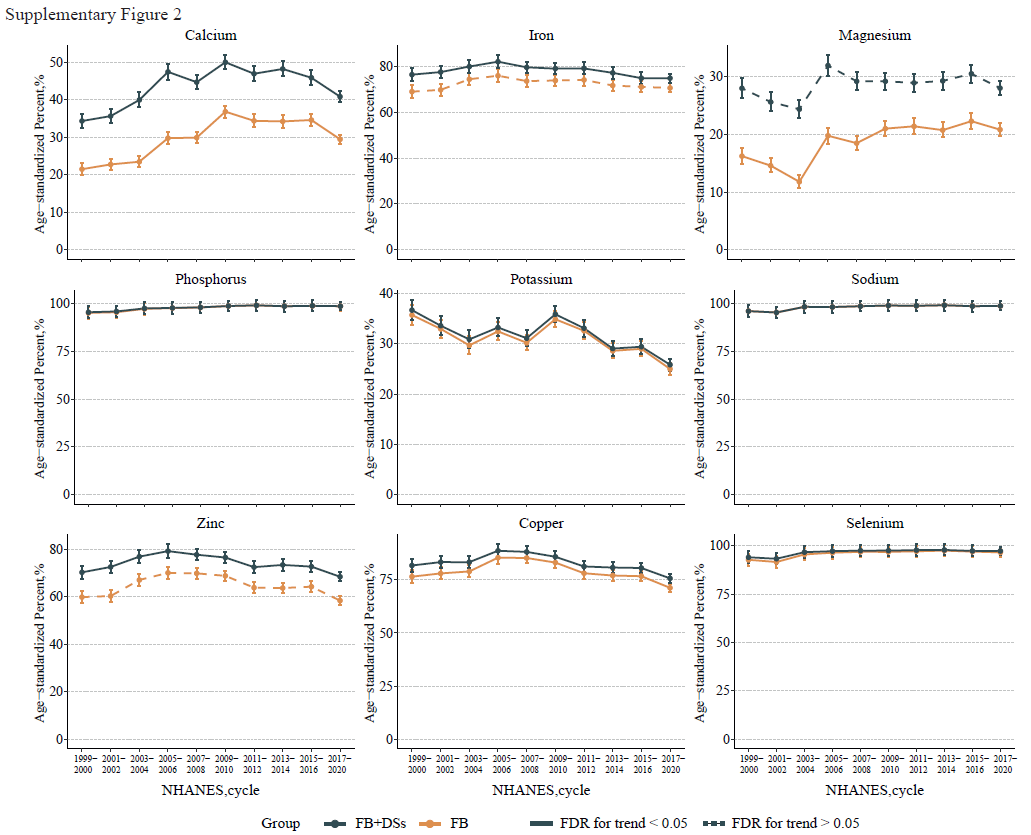


**Supplementary Figure 3. Trends in Estimated Intake of Nine Minerals From FB, DSs, and FB + DSs After Excluding Participants with Diabetes, CVD, and CKD Among U.S. Adults, 1999-2020.**

Data were adjusted for NHANES survey weights to be nationally representative. Analyses are based on energy-adjusted values to 2000 kcal/d using the residual method. All estimates were weighted, and error bars indicated 95% CIs. Results were adjusted for gender, age, race/ethnicity, education level, and family income. Abbreviations: DSs, Dietary supplements; FB, foods and beverages; NHANES, National Health and Nutrition Examination Survey.


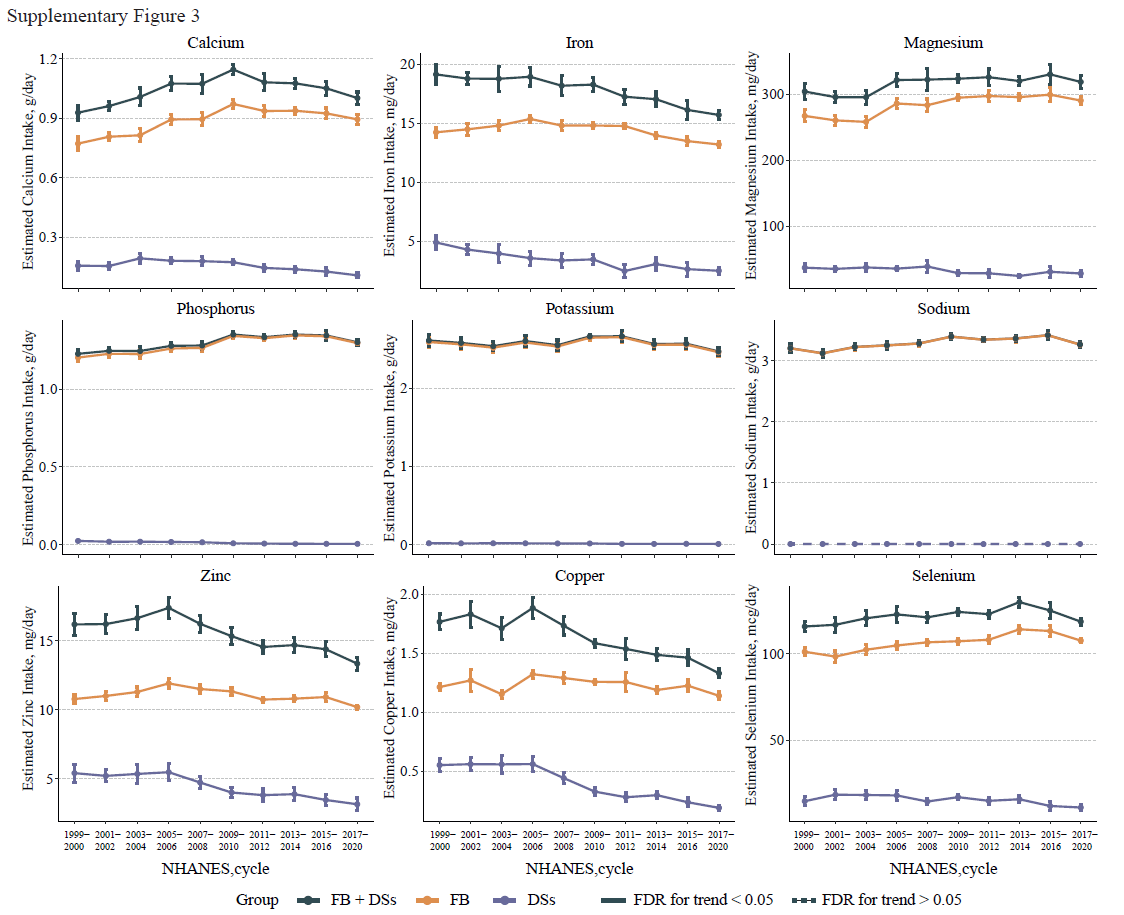


**Supplementary Figure 4. Trends in Age-adjusted Percentage of U.S. Adults Meeting RDAs/AIs After Excluding Participants with Diabetes, CVD, and CKD, 1999-2020.**

Age-adjusted percentage was determined by direct standardization using civilian noninstitutionalized population in 2017-2020 NHANES cycles as a reference (aged 20-39 years, 40-59 years and ≥60 years). Analyses are based on energy-adjusted values to 2000 kcal/d using the residual method. All estimates were weighted, and error bars indicated 95% CIs. Results were adjusted for gender, age, race/ethnicity, education level, and family income. Abbreviations: AIs, Adequate Intakes; CKD, chronic kidney disease; CVD, cardiovascular disease; DSs, Dietary supplements; FB, foods and beverages; NHANES, National Health and Nutrition Examination Survey; RDAs, Recommended Dietary Allowances.


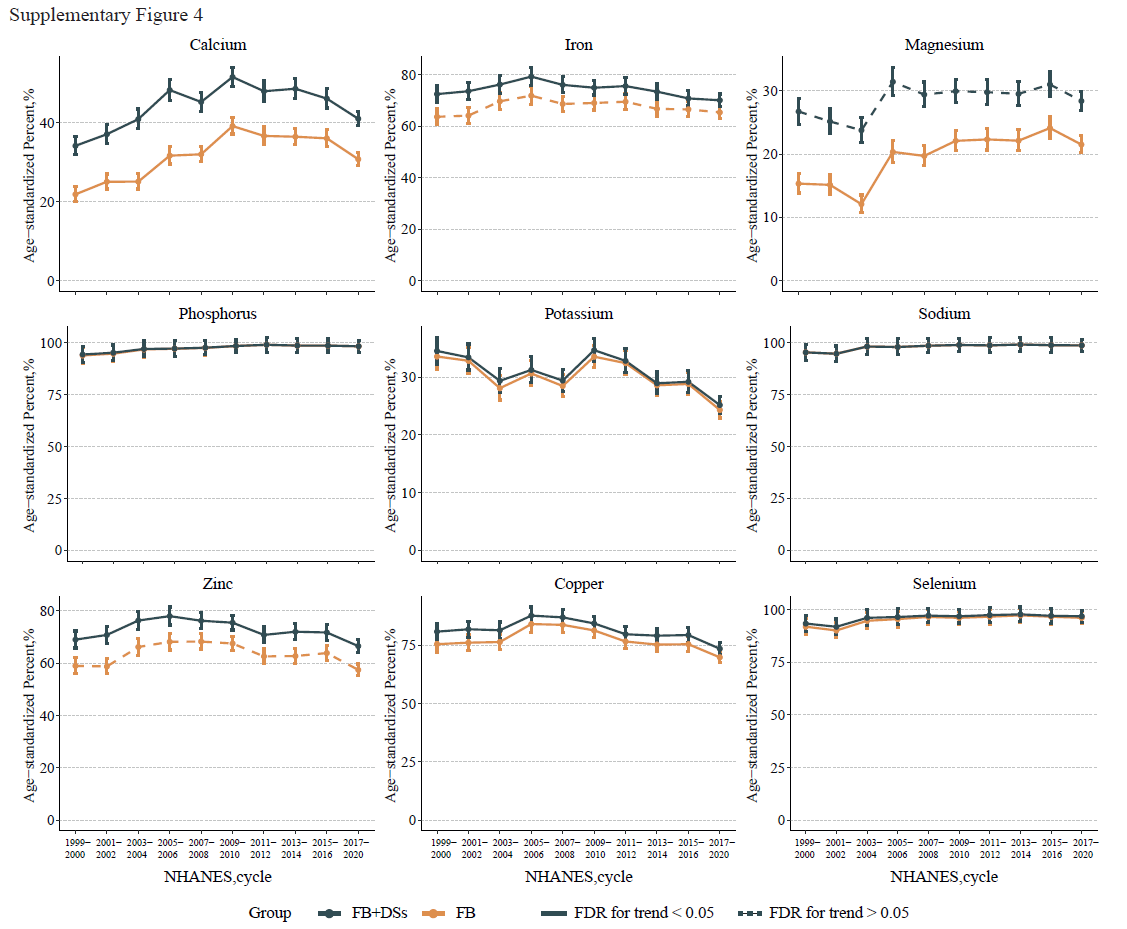


**Supplementary Figure 5. Changes in Estimated Consumption of Dietary Components Among U.S. Adults, 1999-2020.**

All estimates were weighted, and error bars indicated 95% CIs. Results were adjusted for gender, age, race/ethnicity, education level, and family income.


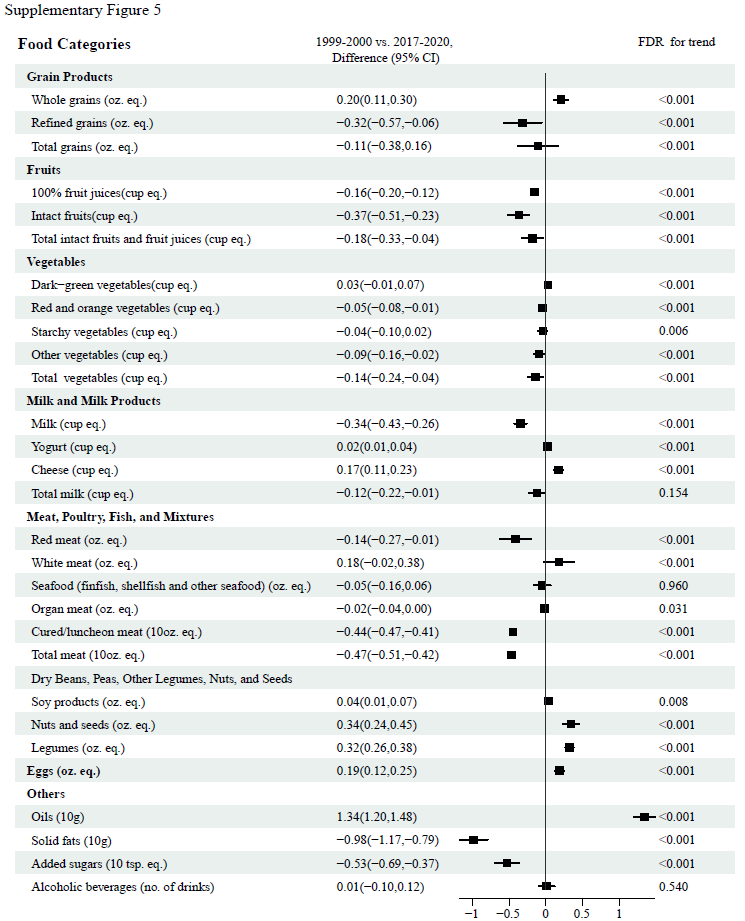


**Supplementary Figure 6. Trends in Estimated Percentage of Nine Minerals (from FB + DSs) From Selected Five Food Sources Among U.S. Adults, 1999-2020.**

Data were adjusted for NHANES survey weights to be nationally representative. Analyses are based on energy-adjusted values to 2000 kcal/d using the residual method. All estimates were weighted, and error bars indicated 95% CIs. Results were adjusted for gender, age, race/ethnicity, education level, and family income. Abbreviations: DSs, Dietary supplements; FB, foods and beverages; NHANES, National Health and Nutrition Examination Survey; Milks, Milk and milk products; Meats, Meat, poultry, fish and mixtures; Eggs, Eggs; Nuts, Legumes, nuts and seeds; Grains, Grain products; Fruits, Fruits; Vegetables, Vegetables; Oils, Fats, oils and salad dressings; Beverages, Sugar, sweeteners and, beverages.


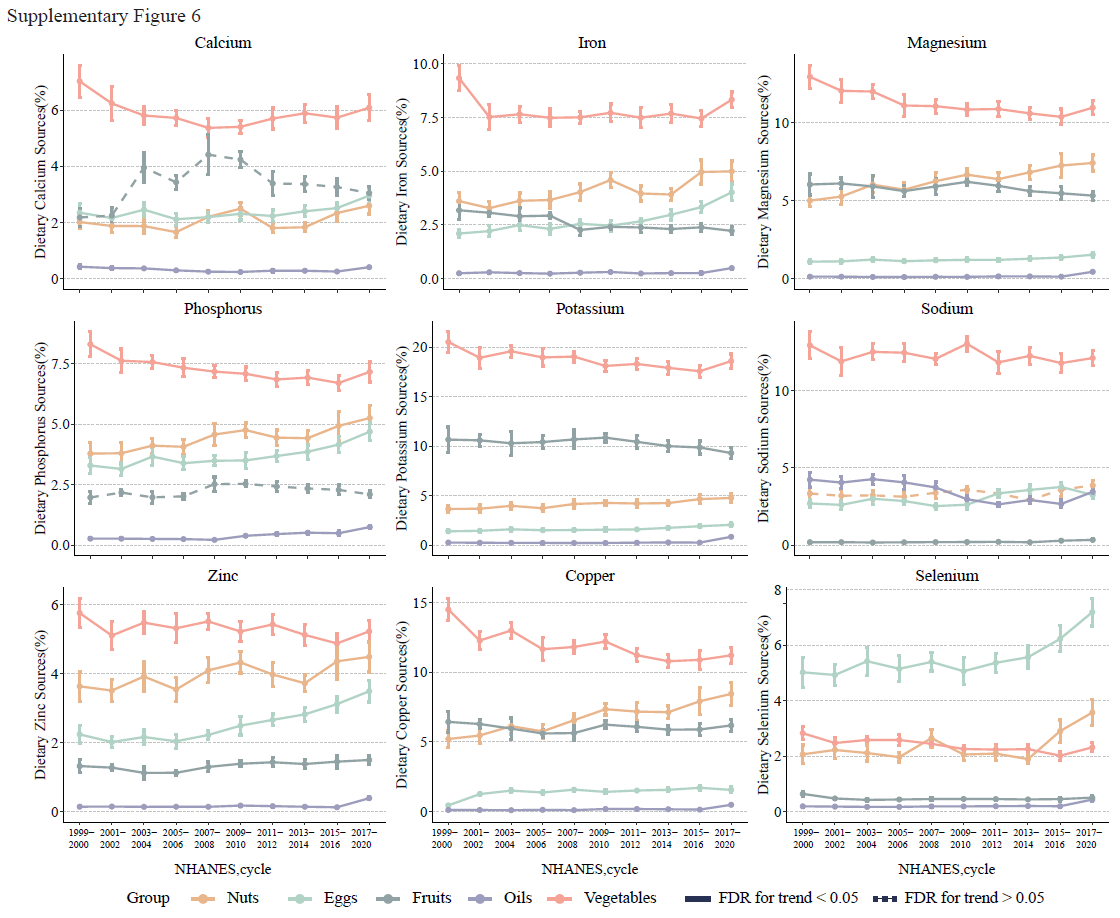


**Supplementary Figure 7. Trends in Estimated Percentage of Nine Minerals (from FB) From Nine Food Sources Among U.S. Adults, 1999-2020.**

Data were adjusted for NHANES survey weights to be nationally representative. Analyses are based on energy-adjusted values to 2000 kcal/d using the residual method. All estimates were weighted, and error bars indicated 95% CIs. Results were adjusted for gender, age, race/ethnicity, education level, and family income. Abbreviations: DSs, Dietary supplements; FB, foods and beverages; NHANES, National Health and Nutrition Examination Survey; Milks, Milk and milk products; Meats, Meat, poultry, fish and mixtures; Eggs, Eggs; Nuts, Legumes, nuts and seeds; Grains, Grain products; Fruits, Fruits; Vegetables, Vegetables; Oils, Fats, oils and salad dressings; Beverages, Sugar, sweeteners and, beverages.


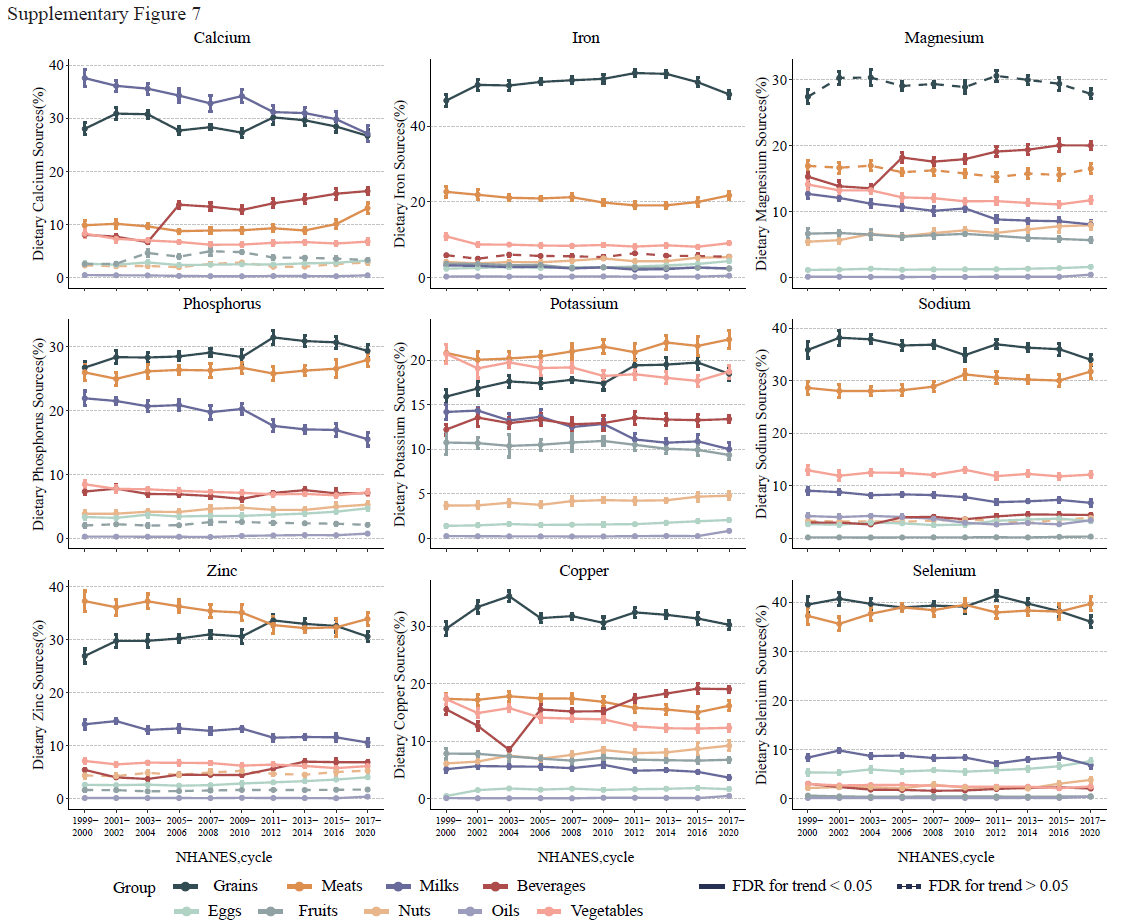


**Supplementary Figure 8. Trends in Estimated Percentage of Nine Minerals (from FB + DSs) From Nine Food Sources and DSs Among U.S. Adults after excluding participants with diabetes, CVD, and CKD, 1999-2020.**

Data were adjusted for NHANES survey weights to be nationally representative. Analyses are based on energy-adjusted values to 2000 kcal/d using the residual method. All estimates were weighted, and error bars indicated 95% CIs. Results were adjusted for gender, age, race/ethnicity, education level and family income. Abbreviations: DSs, Dietary supplements; FB, foods and beverages; NHANES, National Health and Nutrition Examination Survey; Milks, Milk and milk products; Meats, Meat, poultry, fish and mixtures; Eggs, Eggs; Nuts, Legumes, nuts and seeds; Grains, Grain products; Fruits, Fruits; Vegetables, Vegetables; Oils, Fats, oils and salad dressings; Beverages, Sugar, sweeteners and, beverages.


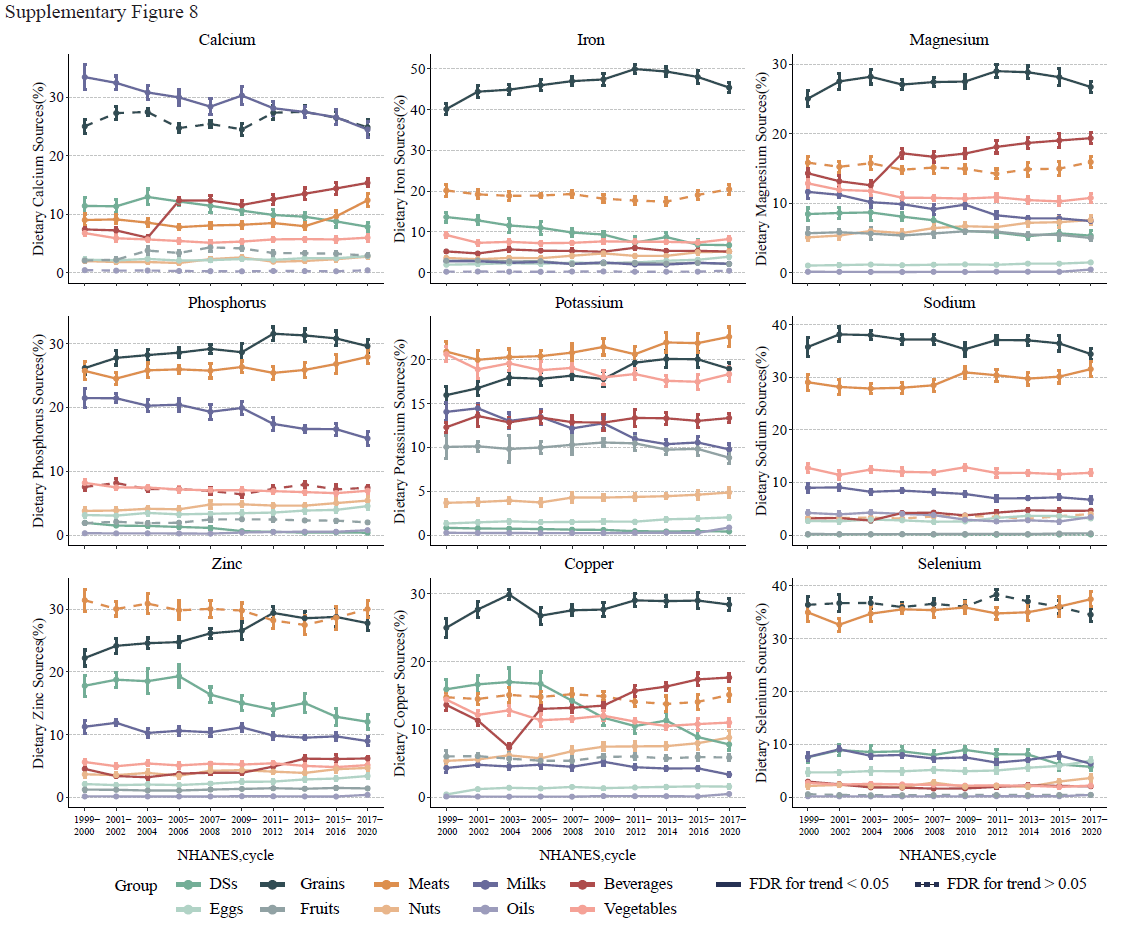


**Supplementary Figure 9. Trends in Estimated Absolute Intake of Nine Minerals from FB + DSs by Gender, 1999-2020.**

Data were adjusted for NHANES survey weights to be nationally representative. Analyses are based on energy-adjusted values to 2000 kcal/d using the residual method. All estimates were weighted, and error bars indicated 95% CIs. Results were adjusted for gender, age, race/ethnicity, education level and family income when appropriate. Abbreviations: DSs, Dietary supplements; FB, foods and beverages; NHANES, National Health and Nutrition Examination Survey.


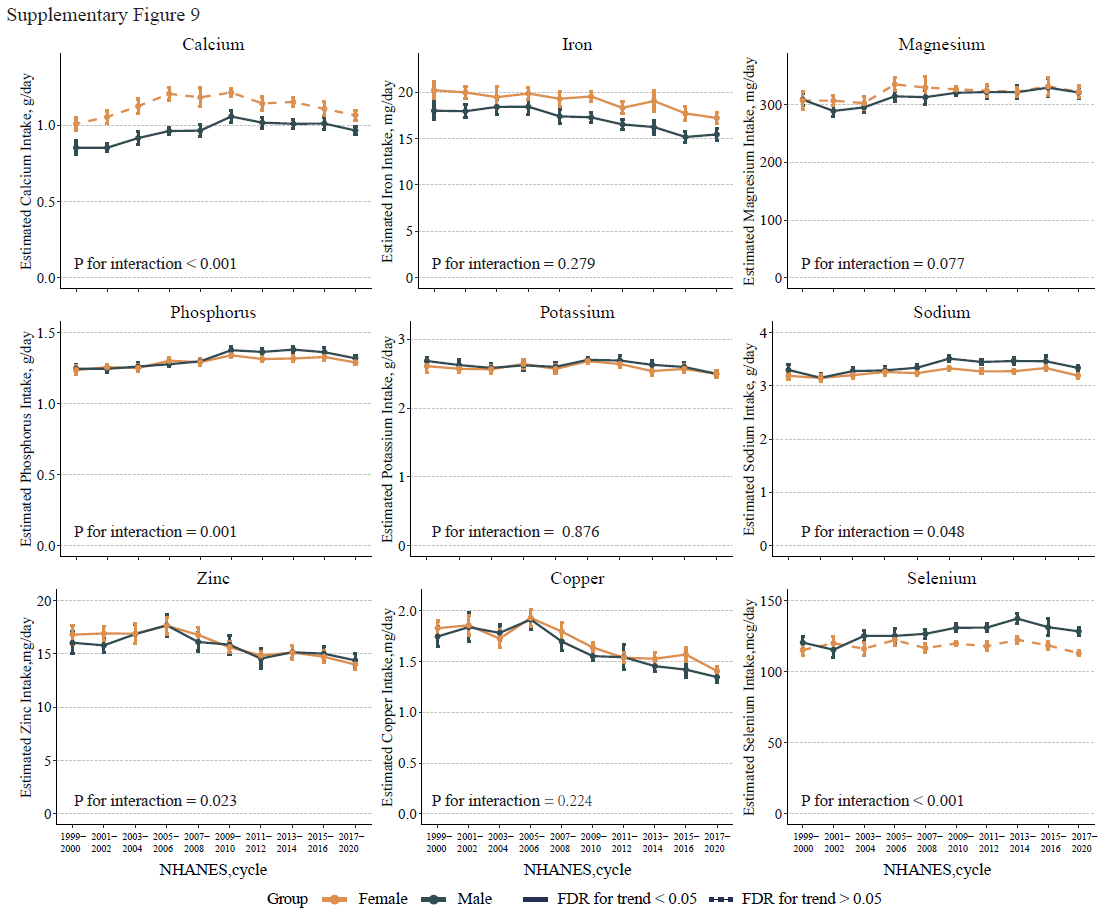


**Supplementary Figure 10. Trends in Estimated Absolute Intake of Nine Minerals from FB + DSs by Age Group, 1999-2020.**

Data were adjusted for NHANES survey weights to be nationally representative. Analyses are based on energy-adjusted values to 2000 kcal/d using the residual method. All estimates were weighted, and error bars indicated 95% CIs. Results were adjusted for gender, age, race/ethnicity, education level and family income when appropriate. Abbreviations: DSs, Dietary supplements; FB, foods and beverages; NHANES, National Health and Nutrition Examination Survey.


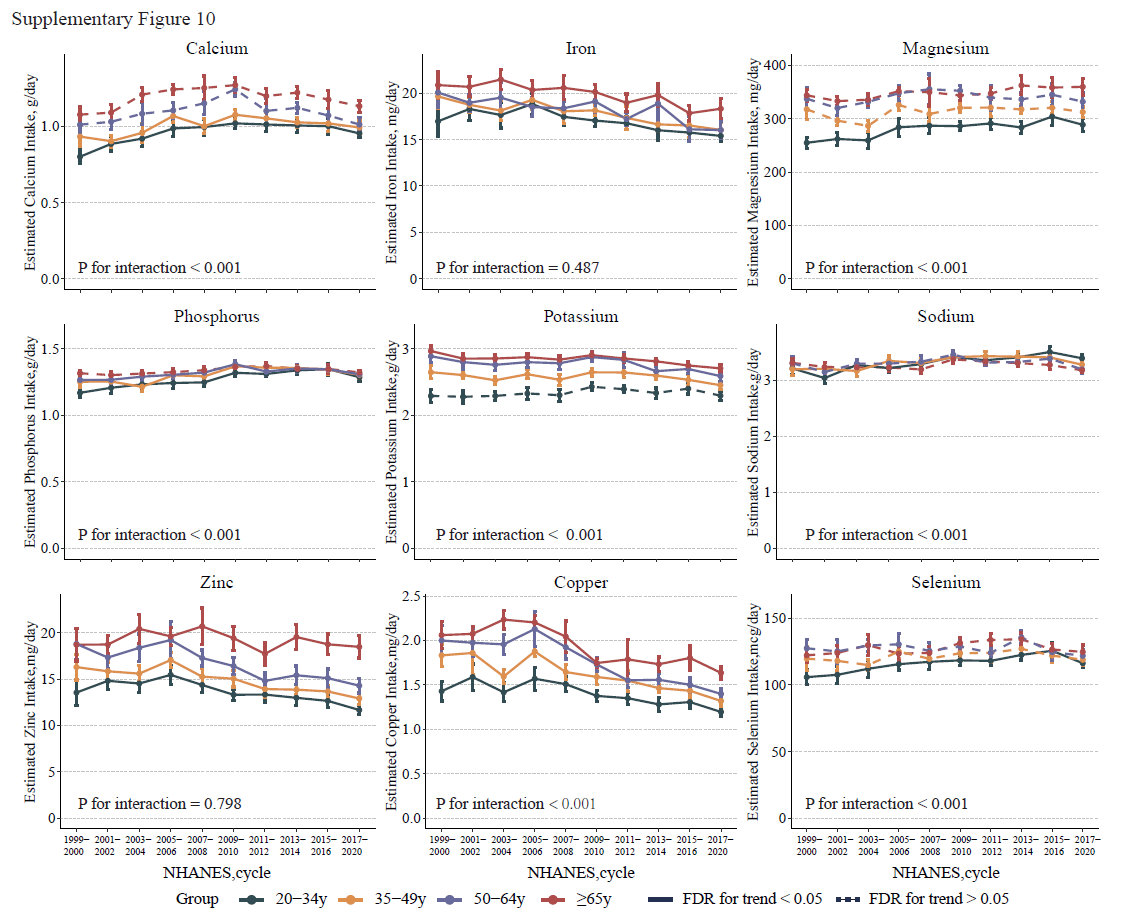


**Supplementary Figure 11. Trends in Estimated Absolute Intake of Nine Minerals from FB + DSs by Race/ethnicity, 1999-2020.**

Data were adjusted for NHANES survey weights to be nationally representative. Analyses are based on energy-adjusted values to 2000 kcal/d using the residual method. All estimates were weighted, and error bars indicated 95% CIs. Results were adjusted for gender, age, race/ethnicity, education level and family income when appropriate. Homogeneity for race/ethnicity subgroups was estimated between White and non-White race/ethnicity groups. Abbreviations: DSs, Dietary supplements; FB, foods and beverages; NHANES, National Health and Nutrition Examination Survey.


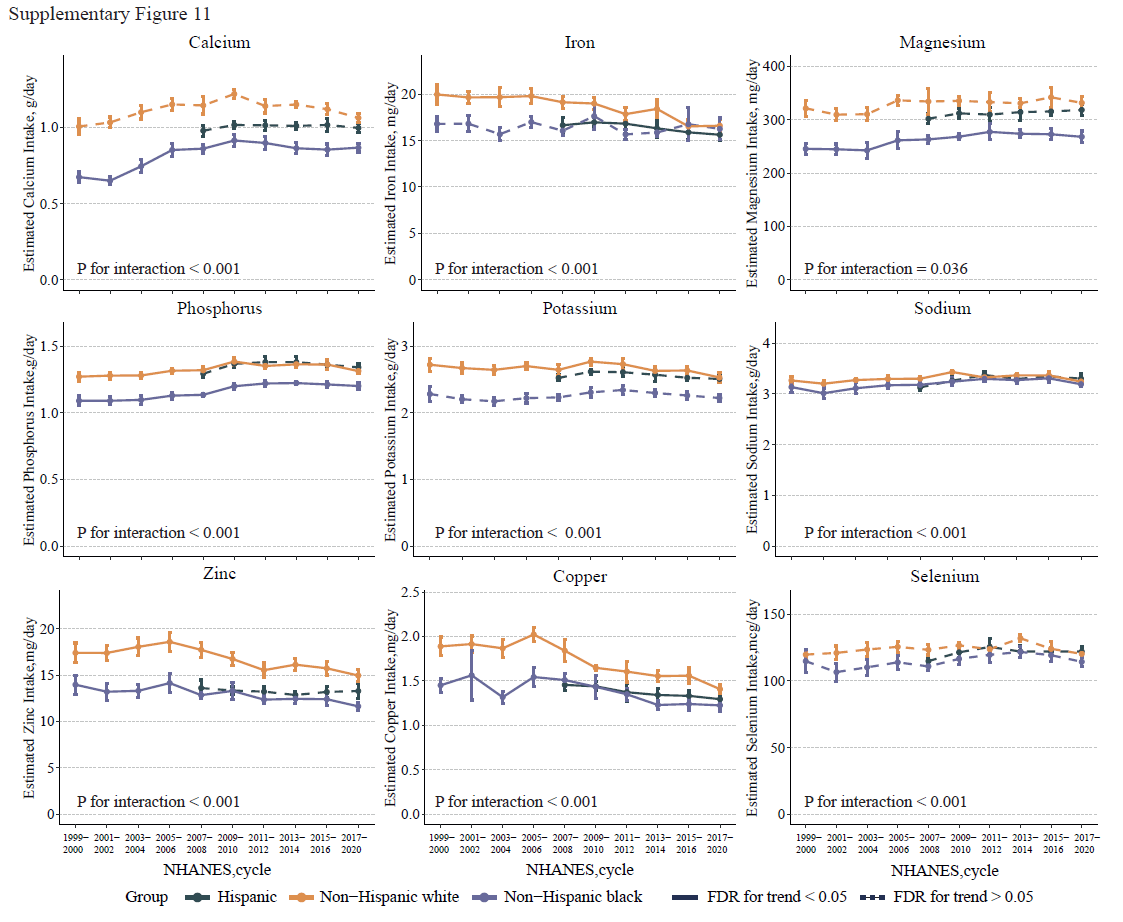


**Supplementary Figure 12. Trends in Estimated Absolute Intake of Nine Minerals from FB + DSs by Education Level, 1999-2020.**

Data were adjusted for NHANES survey weights to be nationally representative. Analyses are based on energy-adjusted values to 2000 kcal/d using the residual method. All estimates were weighted, and error bars indicated 95% CIs. Results were adjusted for gender, age, race/ethnicity, education level and family income when appropriate. Abbreviations: DSs, Dietary supplements; FB, foods and beverages; NHANES, National Health and Nutrition Examination Survey.


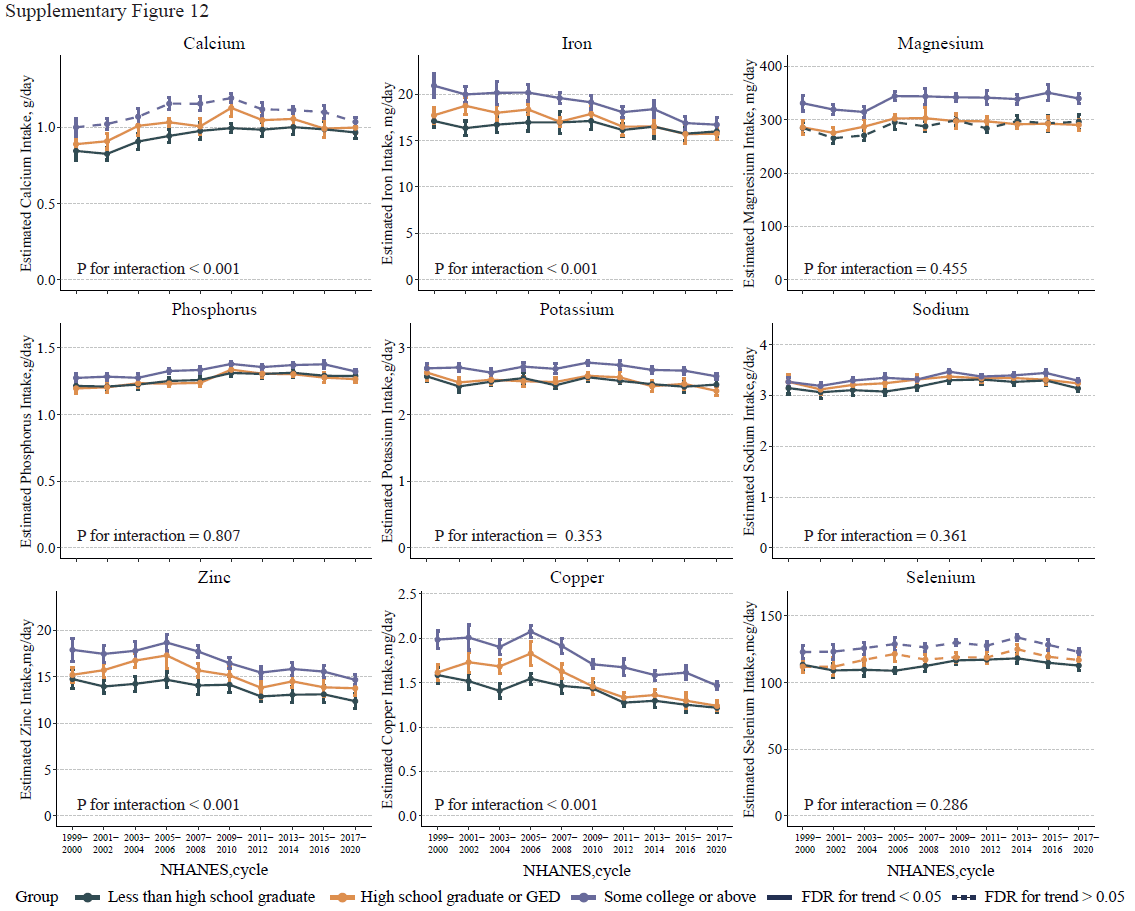


**Supplementary Figure 13. Trends in Estimated Absolute Intake of Nine Minerals from FB + DSs by Family Income, 1999-2020.**

Data were adjusted for NHANES survey weights to be nationally representative. Analyses are based on energy-adjusted values to 2000 kcal/d using the residual method. All estimates were weighted, and error bars indicated 95% CIs. Results were adjusted for gender, age, race/ethnicity, education level and family income when appropriate. Abbreviations: DSs, Dietary supplements; FB, foods and beverages; NHANES, National Health and Nutrition Examination Survey.


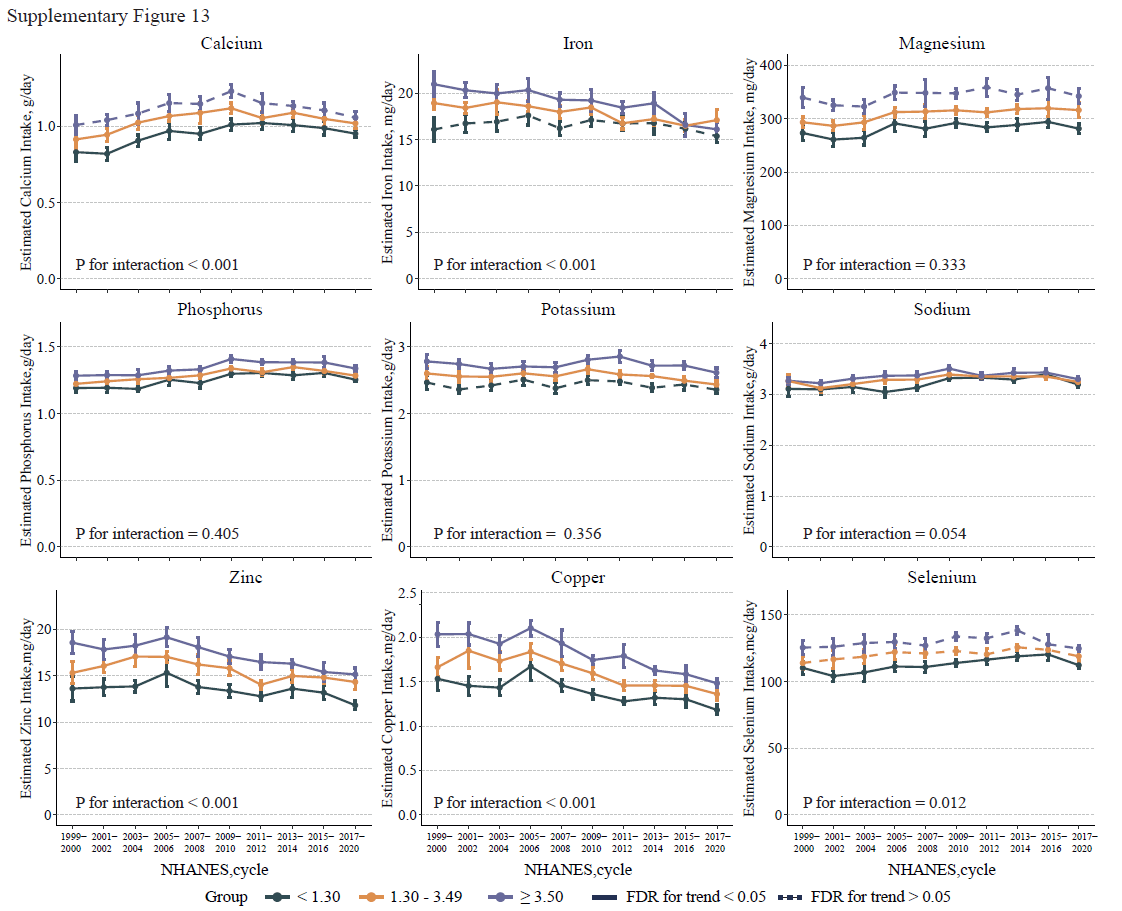

Supplement: Supplementary file 1 — Additional file 1: Supplementary Table 1. Changes in Estimate Intake of Nine Minerals Among U.S. Adults by NHANES Survey Cycle, 1999-2020. Supplementary Table 2. Changes in Estimated Percentage of Nine Minerals Intake from Nine Food Groups and Dietary. Supplements Among U.S. Adults by NHANES Survey Cycle, 1999-2020. Supplementary Table 3. Trends in Estimated Absolute Intake of Nine Minerals from Nine Food Groups and Dietary Supplements by NHANES Survey Cycle, 1999-2020. Supplementary Table 4. Changes in Estimate Intake of Nine Minerals by Sociodemographic Characteristics by NHANES Survey Cycle, 1999-2020. Supplementary Figure 1. Participant flow chart. Supplementary Figure 2. Trends in Age-adjusted Percentage of U.S. Adults Meeting RDAs/AIs by NHANES Survey Cycle, 1999-2020. Supplementary Figure 3. Trends in Estimated Intake of Nine Minerals From FB, DSs, and FB + DSs After Excluding Participants with Diabetes, CVD, and CKD Among U.S. Adults, 1999-2020. Supplementary Figure 4. Trends in Age-adjusted Percentage of U.S. Adults Meeting RDAs/AIs After Excluding Participants with Diabetes, CVD, and CKD, 1999-2020. Supplementary Figure 5. Changes in Estimated Consumption of Dietary Components Among U.S. Adults, 1999-2020. Supplementary Figure 6. Trends in Estimated Percentage of Nine Minerals (from FB + DSs) From Selected Five Food Sources Among U.S. Adults, 1999-2020. Supplementary Figure 7. Trends in Estimated Percentage of Nine Minerals (from FB) From Nine Food Sources Among U.S. Adults, 1999-2020. Supplementary Figure 8. Trends in Estimated Percentage of Nine Minerals (from FB + DSs) From Nine Food Sources and DSs Among U.S. Adults after excluding participants with diabetes, CVD, and CKD, 1999-2020. Supplementary Figure 9. Trends in Estimated Absolute Intake of Nine Minerals from FB + DSs by Gender, 1999-2020. Supplementary Figure 10. Trends in Estimated Absolute Intake of Nine Minerals from FB + DSs by Age Group, 1999-2020. Supplementary Figure 11. Trends in Estimate [file 12937_2024_950_MOESM1_ESM.docx]
